# Natural Acetogenins, Chatenaytrienins-1, -2, -3 and -4, Mitochondrial Potential Uncouplers and Autophagy Inducers—Promising Anticancer Agents

**DOI:** 10.3390/antiox12081528

**Published:** 2023-07-30

**Authors:** Lilya U. Dzhemileva, Regina A. Tuktarova, Usein M. Dzhemilev, Vladimir A. D’yakonov

**Affiliations:** N.D. Zelinsky Institute of Organic Chemistry, Russian Academy of Sciences, Leninsky Prospect 47, Moscow 119991, Russia; regina-tuktarova@yandex.ru (R.A.T.); dzhemilev@anrb.ru (U.M.D.)

**Keywords:** acetogenins, chatenaytrienins, cytotoxicity, autophagy, flow cytometry, apoptosis, cell cycle, Luminex xMAP technology

## Abstract

The present paper details the complete stereoselective synthesis of four natural acetogenins, chatenaytrienins-1, -2, -3 and -4, previously isolated from the roots of fruit trees of the family Annonaceae (*A. nutans* and *A. muricata*), as an inseparable mixture. The novel organometallic reactions, developed by the authors, of Ti-catalyzed cross-cyclomagnesiation of O-containing and aliphatic allenes using available Grignard reagents were applied at the key stage of synthesis. We have studied the biological activity of the synthesized individual chatenaytrienins-1, -2, -3 and -4 in vitro, including their cytotoxicity in a panel of tumor lines and their ability to induce apoptosis, affect the cell cycle and mitochondria, and activate the main apoptotic signaling pathways in the cell, applying modern approaches of flow cytometry and multiplex analysis with Luminex xMAP technology. It has been shown that chatenaytrienins affect mitochondria by uncoupling the processes of mitochondrial respiration, causing the accumulation of ROS ions, followed by the initiation of apoptosis. The most likely mechanism for the death of cortical neurons from the consumption of tea from the seeds of Annona fruit is long-term chronic hypoxia, which leads to the development of an atypical form of Parkinson’s disease that is characteristic of the indigenous inhabitants of Guam and New Caledonia.

## 1. Introduction

Fruit trees of the family Annonaceae have a good record of medicinal use with a wide range of applications in traditional and alternative medicine in some countries, especially in Central and South America. Spanish navigators brought these plants to the Antilles and the Pacific Islands. All parts of the plant (bark, leaves, roots, seeds, and fruit) are used as food in the Caribbean and known as a source of biologically active compounds. More than 40% of the people of Guadeloupe drink herbal tea made from Annonaceae fruit, although this drink is not considered an official medicine. The toxicity of these herbal mixes has not been thoroughly studied yet, and the correlation between the benefits of these teas and the risk of toxic effects from the original components remains uncertain [1,2]. The edible pulp of these plants is an important fruit resource with high palatability in many parts of the world. For example, the cream apple (fruit of *A. cherimola* or *A. reticulata*) is an important fruit widely consumed in Thailand. In addition to the wonderful, sweet taste and excellent aroma, cream apples offer health benefits and a rich set of vitamins (A, B1, B2, B3, B5, B9, C, and E) and minerals (Ca, Cu, Fe, Mg, Mn, P, and Zn) [3]. This fruit tree is widely cultivated in Australia, India, South Africa, Somalia, Eritrea, the Mediterranean (Spain, Israel, Portugal, Italy, Egypt, Libya, and Algeria), the Philippines, the Hawaiian Islands, and Sri Lanka. Annona cherimola is common in Southeast Asia, especially in Vietnam and Cambodia [3].

In 1999, Caparros-Lefebvre et al. published a scientific article on an unexpectedly high rate of atypical parkinsonism in Guadeloupe (French West Indies, French Polynesia) registered during an epidemiological study in 1996. The work reported that progressive supranuclear palsy (PSP) and atypical parkinsonism occurred precisely in those patients in Guadeloupe who used to drink herbal tea or eat the fruit of the so-called custard apple from the family Annonaceae. Initially, the authors suggested that the disease occurred due to chronic poisoning when eating neurotoxins from tropical herbal teas and fruits of *A. muricata*, *A. squamosa*, and *A. reticulata* [4,5].

Annonaceous acetogenins were discovered as a result of studies aimed at the isolation and identification of organic compounds contained in Annona fruit. The main mechanism of action of this relatively new class of bioactive compounds implied the inhibition of NADH-ubiquinone oxidoreductase. Acetogenins are a unique and structurally homogeneous class of polyketides. The fatty acid-derived structure provides high lipophilicity and allows them to freely cross biological membranes. The possible toxicity of different acetogenins has been studied for several years in vitro, proving them to be the most potent inhibitors of tissue respiration processes known so far [6,7,8,9]. 

All in vitro and in vivo studies of the neurotoxicity of Annona products provide evidence of an association between the consumption of the seeds of Annona fruit and the occurrence of atypical Parkinson’s disease. Exactly the same form of atypical course of Parkinson’s disease was found in local residents of Guam and New Caledonia who traditionally consumed fruits and seeds of plants of the family Annonaceae [10,11].

Currently, several research groups around the world are actively engaged in the synthesis of natural acetogenins as well as their precursor, muricadienin, developing interesting and promising synthetic approaches to creating promising anticancer drugs with fundamentally new molecular targets, low toxicity, and minimal side effects [12,13]. The method we developed made it possible to achieve the highest yield of the reaction products (up to 92%) compared to approaches reported earlier [14,15]. Taking into account the great biomedical potential of natural acetogenins as powerful and highly effective inhibitors of the respiratory chain in mitochondria [16], as well as the growing interest of pharmacologists and specialists in the fields of organic and medicinal chemistry in the chemical compounds contained in various parts of plants of the family Annonaceae [17,18] towards creating new approaches for the production of natural compounds containing bis-methylene with separated Z-double bonds in their structure, our group for the first time carried out the complete stereoselective synthesis of all four natural acetogenins, chatenaytrienins-1, -2, -3 and -4, previously isolated by another scientific group from the roots of fruit trees of the family Annonaceae family (*A. nutans* and *A. muricata*), in the form of an inseparable mixture [19]. The present work details the study of the antitumor activity of the synthesized individual chatenaytrienins-1, -2, -3 and -4 (1–4) in vitro; their cytotoxicity in a panel of tumor lines (Jurkat, K562, U937, and HL60), conditionally normal cells (Hek293), as well as normal fibroblasts; their ability to induce apoptosis and influence the cell cycle and mitochondria; their ability to inhibit the most versatile cell viability signaling pathways (phosphorylated and non-phosphorylated fractions of tyrosine kinases CREB, JNK, NFkB, p38, ERK1/2, Akt, p70S6K, STAT3 and STAT5); their genotoxicity (Chk2, Chk1, MDM2, H2A.X, p21, p53, and ATR); and their ability to induce the main apoptotic proteins (BAD, Cas8, Bcl-2, Cas9, JNK, p53, and Akt) responsible for cell proliferation as well as for the initiation of apoptosis.

## 2. Materials and Methods

### 2.1. Apparatus and Chemical Materials

1-Dodecyne, 1-tetradecyne, lithium acetylide, ethylene diamine complex, nickel (II) acetate tetrahydrate (Ni(OAc)2·4H2O), dicyclohexylamine, copper (I) iodide (CuI), bis(cyclopentadienyl)titanium (IV) dichloride (Cp2TiCl2), 4-dimethylaminopyridine (DMAP), N,N′-dicyclohexylcarbodiimide (DCC), sodium cyanoborohydride (NaBH3CN), trifluoromethanesulfonic anhydride (Tf2O), and tris(dibenzylideneacetone)dipalladium(0) (Pd2(dba)3) were obtained from Sigma-Aldrich and Acros Organics. All solvents were dried (1,4-dioxane, tetrahydrofurane, diethyl ether over Na) and freshly distilled before use. All reactions were carried out under an argon atmosphere. 1H and 13C NMR spectra were obtained using a Bruker Ascend 500 spectrometer in CDCl3 operating at 500 MHz for 1H and 125 MHz for 13C and a Bruker AVANCE 400 spectrometer in CDCl3 operating at 400 MHz for 1H and 100 MHz for 13C. IR spectra were recorded on a Bruker VERTEX 70V using KBr discs over the range of 400–4000 cm^−1^. Melting points were recorded on a Stuart SMP3. Mass spectra of MALDI TOF/TOF positive ions (matrix of sinapic acid) were recorded on a mass spectrometer Bruker Autoflex^TM^ III Smartbeam. High-resolution mass spectra (HRMS) were measured on a MaXis Impact instrument (Bruker) using a time-of-flight mass analyzer (TOF) with electrospray ionization (ESI). In experiments on selective collisional activation, the activation energy was set at maximum abundance of fragment peaks. Syringe injection was used for solutions in MeCN (flow rate 5 µL/min). Nitrogen was applied as a dry gas; the interface temperature was set at 180 °C. Individuality and purity of the synthesized compounds were controlled by TLC on Sorbfil plates; anisic aldehyde in acetic acid was used as the developer. Column chromatography was carried out on Acrus silica gel (0.060–0.200 mm). All compounds for which in vitro studies were conducted were >95% pure. The purity of the compounds was confirmed by elemental analysis, high resolution mass-spectrometry, and 1H NMR spectra. 

### 2.2. Chemical Synthesis

The synthesis methods for 1,2,6Z-alkatrienes (15, 16) along with the 1H and 13C NMR data can be found in the literature [20]. The general procedure for cross-cyclomagnesiation of 1,2,6Z-trienes (15, 16) and tetrahydropyran ethers of 1,2-allene alcohols (17–19) by EtMgBr in the presence of Mg metal and the Cp2TiCl2 catalyst, as well as the general procedures for the preparation of 1Z,5Z,9Z-dienoic acids (28–31), the synthesis of trienes (32–35), the synthesis of triflates (36–39), the synthesis of chatenaytrienins-1, -2, -3 and -4 (1–4), and the 1H and 13C NMR data for compounds (24, 28, 32, 36, 1) can be found in the literature [21]. The 1H and 13C NMR data of compounds (25–27), (29–31), (33–35), (37–39), and (2–4) can be found in Supplementary Materials.

### 2.3. Cell Culturing

Cells, including Jurkat E6.1 (human leukemic T cell lymphoblasts, catalogue no. 88042803), K562 (human chronic myelogenous leukemia cells, catalogue no. 89121407), U937 (human Caucasian histiocytic lymphoma cells, catalogue no. 85011440), HL60 (human Caucasian promyelocytic leukemia cells, catalog no. 98070106), HEK293 (human embryonic kidney cells with adenovirus, catalog no. 85120602), and human lung fibroblasts Ce (catalogue no. 90011883), were purchased from the European Collection of Authenticated Cell Cultures (UK Health Security Agency) and cultured according to standard protocols using sterile techniques. The cell lines were shown to be free of viral contamination and mycoplasma. Cells were maintained in RPMI 1640 (Jurkat, K562, U937, and HL60) and DME M (fibroblast and HEK293) (Gibco) supplemented with 4 µM glutamine, 10% FBS (Sigma), and 100 units/mL penicillin-streptomycin (Sigma). All types of cells were grown under an atmosphere of 5% CO_2_ at 37 °C. The cells were subcultured at 2–3-day intervals. Cells were then seeded in 24-well plates at 5 × 10^4^ cells per well and incubated overnight. Jurkat, K562, U937, HL60, HEK293, and fibroblast cells were subcultured at 2-day intervals with a seeding density of 1 × 10^5^ cells per well in 24-well plates in RPMI with 10% FBS.

### 2.4. Cytotoxicity Assay

Viability (live/dead) assessment was performed by staining cells with 7-AAD (7-aminoactinomycin D; Biolegend). After treatment, cells were harvested, washed 1–2 times with phosphate-buffered saline (PBS), and centrifuged at 400× *g* for 5 min. Cell pellets were resuspended in 200 µL of flow cytometry staining buffer (PBS without Ca^2+^ and Mg^2+^, 2.5% FBS) and stained with 1 mM/L of 7-AAD staining solution for 15 min at room temperature in the dark. Samples were assessed using the NovoCyte Penteon Flow Cytometer System (Agilent, 5301 Stevens Creek Blvd., Santa Clara, CA, USA). Detection of 7-AAD emission was collected through a 675/30 nm filter in the FL4 channel.

### 2.5. Detection of Mitodamage

In this work, we demonstrated a cytometric analysis that allowed us to multiparametrically assess three markers of cell health: changes in the mitochondrial potential, expression of phosphatidylserine on the cell surface, and membrane permeabilization. The use of reagents MitoSense Red, annexin V, and 7-AAD in the Millipore FlowCellect™ MitoDamage Kit allowed us to gain information on early, mid, and late apoptosis with one simple assay. Cells were treated with the synthesized compounds at a concentration of 0.5 µM and incubated at 37 °C for 4 h. After this time, the cells were dissociated using an accutase solution, stained, and analyzed using the NovoCyte Penteon Flow Cytometer System (Agilent, 5301 Stevens Creek Blvd., Santa Clara, CA, USA), according to the manufacturer’s protocols for the FlowCellect™ MitoDamage Kit and FlowCellect™ Oxidative Stress Characterization Kit (Merck).

### 2.6. Cell Cycle Analysis

The cell cycle was analyzed by propidium iodide staining (Guava^®^ Cell Cycle Reagent 4500-0220). After treatment and incubation of cells for 24 h, they were collected, washed 1–2 times with phosphate-buffered saline (PBS), and centrifuged at 450× *g* for 5 min. The cell pellet was resuspended in 200 μL of flow cytometry staining buffer (PBS without Ca^2+^ and Mg^2+^, 2.5% FBS). Cells were then seeded in 24-well plates at a density of 15 × 10^5^ cells per well, centrifuged at 450× *g* for 5 min, then fixed with ice-cold 70% ethanol for 24 h at 0 °C. Before staining with propidium iodide, cells were washed with PBS and incubated with 250 µL of cell cycle detection reagent (Millipore) for 40 min at 22 °C in the dark. Samples were analyzed using the NovoCyte Penteon Flow Cytometer System (Agilent, 5301 Stevens Creek Blvd., Santa Clara, CA, USA).

### 2.7. Assessment of Cytochrome C Loss

Quantification of cytochrome c release from mitochondria in apoptotic cells was presented to detect the mitochondrial pathway of apoptosis in cells using flow cytometry with the FlowCellect™ Cytochrome c Kit (FCCH100110). For this, cells were treated with FITC-conjugated antibodies against cytochrome c and control anti-IgG1-FITC, together with the use of an optimized fixation procedure, permeabilization, and blocking buffer (Luminex^®^, USA). Higher levels of cytochrome c fluorescence were observed in living cells, while lower levels were characteristic of apoptotic cells in which cytochrome c was released from mitochondria into the cytoplasm. Jurkat cells were incubated for 4 h with the synthesized substances at a concentration corresponding to their 24-h CC50 in a culture plate and assessed using the NovoCyte Penteon Flow Cytometer System (Agilent, 5301 Stevens Creek Blvd., Santa Clara, CA, USA). The obtained data were processed using NovoExpress^®^ software (ACEA).

### 2.8. Assessment of Mitochondrial Potential

Changes in the mitochondrial membrane potential (ΔΨ) in Jurkat cells induced by treatment with the synthesized compounds were detected using MitoSOX Red, a positively charged probe that accumulates rapidly in mitochondria and as such can be used to detect superoxide/ROS production within mitochondria using fluorometry, microscopy, or flow cytometry. Live cells induced a low level of MitoSOX Red fluorescence, while cells with scattered mitochondrial membrane potential caused much higher MitoSOX Red fluorescence. Annexin V is a dye commonly used to detect early signs of apoptosis. Control cells showed no fluorescence, while apoptotic cells showed positive green fluorescence due to phosphatidylserine externalization and positive MitoSOX Red fluorescence due to mitochondrial potential dissipation and ROS accumulation. Thus, using two different dyes, MitoSOX Red and annexin V (CF488A), in the FlowCellect™ MitoStress Kit (FCCH100109), we analyzed the mitochondrial membrane potential dissipation and associated apoptosis in Jurkat cells in response to the synthesized chatenaytrienins.

### 2.9. Assessment of Autophagy

Autophagy is the pathway by which cytoplasmic materials, including macromolecules, organelles, and pathogens, are delivered to lysosomes for degradation, and this process is associated with the reuse of intracellular organelles.

Autophagy is involved in various physiological processes and its changes are associated with the pathogenesis of diseases such as neurodegenerative diseases, cardiovascular complications, cancer, and infectious diseases, as well as with physiological processes such as innate and specific immunity, pathogen clearance, lymphocyte selection, antigen presentation, and production of immunoglobulins. Therefore, the determination of LC3 protein inside autophagosomes without the cytosolic form of LC3-I at the stage of selective cell permeability was measured using flow cytometry. Thus, using the FlowCellect™ LC3-GFP Reporter Autophagy Assay Kit (FCCH100170 and FCCH100181), flow cytometry allowed for a rapid assay that could be combined with other phenotypic and functional markers to characterize different cell populations.

### 2.10. Analysis of Genotoxity and Early Apoptosis

Multiplex analysis was performed according to a previously published method [20]. MILLIPLEX^®^MAP technology is used to determine the total protein or phosphorylated protein levels of various biological analytes, such as kinases or signaling proteins, including CREB, ERK/MAP, p70 S6, p21, p38, PI3K/AKT/mTOR, JNK (A), p53, MDM2, Bcl-2/Bax, STAT3 and STAT5, Nf-kB, Caspase 3-8-9, JNK, ATM, Chk1, Chk2, and H2AX, in cell lysates using the Luminex^®^ system ((MILLIPLEX Multi-Pathway Magnetic Bead 9-Plex—Cell Signaling Multiplex Assay kit, Merk, Germany). This MILLIPLEX^®^MAP technology provides an alternative to Western blotting and immunoprecipitation and has several important advantages, such as more accurate measurement of small amounts of protein and the elimination of analyte loss during sample preparation. The multiplex immunoassay (MILLIPLEX 48-680MAG; Merck Millipore, Germany) was performed in Millipore 96-well plates to detect changes in concentrations of phosphorylated and unphosphorylated proteins p53, MDM2, p21, p38, PI3K/AKT/mTOR, Bcl-2/Bax, STAT3 and STAT5, Nf-kB, Caspase 3 -8-9, CREB, ERK/MAP, p70 S6, JNK, ATM, Chk1, Chk2, and H2AX in Jurkat cell lysates.

### 2.11. Statistics

Data are expressed as mean ± SD or mean ± SE where indicated of at least triplicate determinations. Statistical comparisons between groups were performed by using the Student’s *t*-test. Differences were considered significant at *p* < 0.05.

## 3. Results 

### 3.1. Chemistry

**Figure 1:**
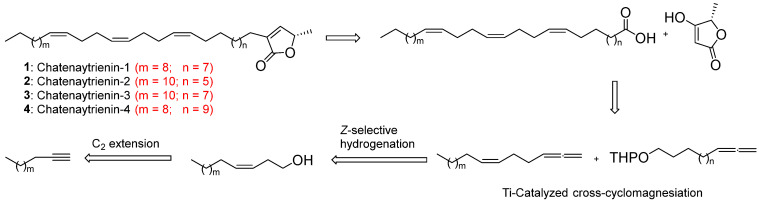
Retrosynthetic analysis of chatenaytrienins-1, -2, -3 and -4 (1–4).

Earlier, our group was the first to carry out a low-step complete synthesis of natural acetogenins, that is, chatenaytrienin-1 and muricadienin [21,22,23]. The reported synthesis scheme was based on our reaction of Ti-catalyzed intermolecular cross-cyclomagnesiation of aliphatic and O-containing 1,2-dienes [24,25,26]. A major asset of the suggested approach was the possibility of extending this technique for the rapid synthesis of any homologs of chatenaytrienin-1 by varying the number of methylene units in the initial 1,2-dienes. Therefore, to synthesize and subsequently produce the above-mentioned individual chatenaytrienins-1, -2, -3 and -4 (**1**–**4**), a scheme for the retrosynthesis of acetogenins was developed (Figure 1).

According to the above scheme, proceeding to the synthesis of chatenaytrienins-1, -2, -3 and -4 (1–4), 1,2,6Z-alkatrienes were initially synthesized, which, as a result of the cross-cyclomagnesiation reaction with tetrahydropyran esters of various O-containing 1,2-dienes, led to bis-methylene-separated trienoic acids. At the final stage of the synthesis involving the Fries rearrangement, a terminal α-substituted fragment of butenolide was formed (Figure 1).

**Figure 2:**
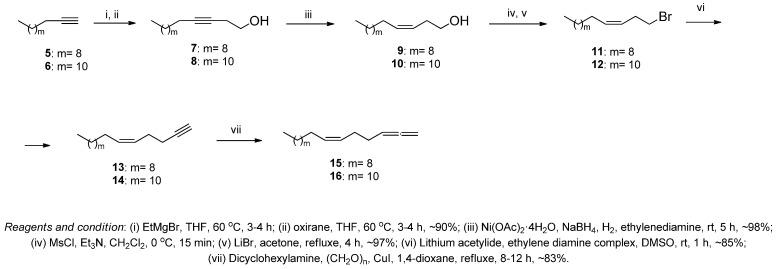
Chemoselective synthesis of 1,2,6Z-trienes (15, 16).

The starting 1,2,6Z-alkatrienes (15, 16) were synthesized in 5 steps, proceeding from commercially available terminal alkynes for the subsequent preparation of bis-methylene-separated trienoic acids. At the first stage, dodecin-1 (5) and tetradecin-1 (6) were treated with EtMgBr in THF under heating, and the resulting intermediate organomagnesium compounds were reacted with ethylene oxide (Figure 2). Subsequent stereoselective hydrogenation of the resulting alcohols (7, 8) with molecular hydrogen in the presence of a Brown’s P2–Ni catalyst [27] gave unsaturated alcohols with the Z-configuration of the double bond (9, 10) in ~98% yield. At the next stage, successive reactions of alcohols (9, 10) with mesyl chloride and treatment of mesylates with lithium bromide gave bromides (11, 12), the ethynylation of which with lithium acetylenide produced alkynes (13, 14) in ~85% yield. Key allenes, (6Z)-heptadeca-1,2,6-triene (15) and (6Z)-nonadeca-1,2,6-triene (16), were synthesized based on alkynes (13, 14) using the Crabbé method [28] by boiling with formaldehyde, dicyclohexylamine, and copper iodide (Figure 2).

**Figure 3:**
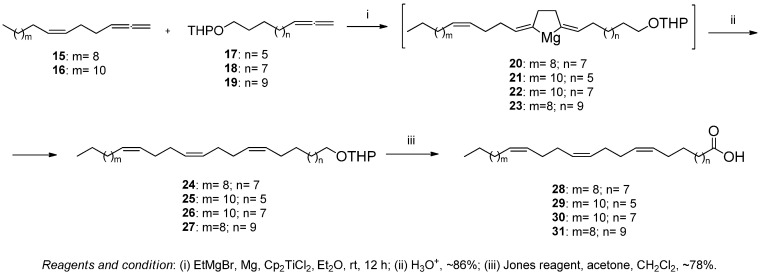
Ti-Catalyzed cross-cyclomagnesiation of 1,2-dienes.

The cross-cyclomagnesiation reaction of Z-alkenyl allenes (15, 16) and the corresponding tetrahydropyran esters of allene alcohols 2-(undeca-9,10-dien-1-yloxy)tetrahydro-2H-pyran (17), 2-(trideca-11,12-dien-1-yloxy)tetrahydro-2H-pyran (18), and 2-(pentadeca-13,14-dien-1-yloxy)tetrahydro-2H-pyran (19) were synthesized according to the approach reported earlier [21], using EtMgBr in the presence of Mg (powder) and the Cp2TiCl2 catalyst (10 mol.%) at room temperature (Figure 3). The reactions proceeded through the formation of intermediate magnesacyclopentanes (20–23), the hydrolysis of which led to the formation of (Z,Z,Z)-trien-1-ols tetrahydropyran esters (24–27) in ~85% yield. At the final stage, the oxidation of tetrahydropyran esters (24–27) with Jones reagent produced the target Z,Z,Z-trienoic acids (28–31).

**Figure 4:**
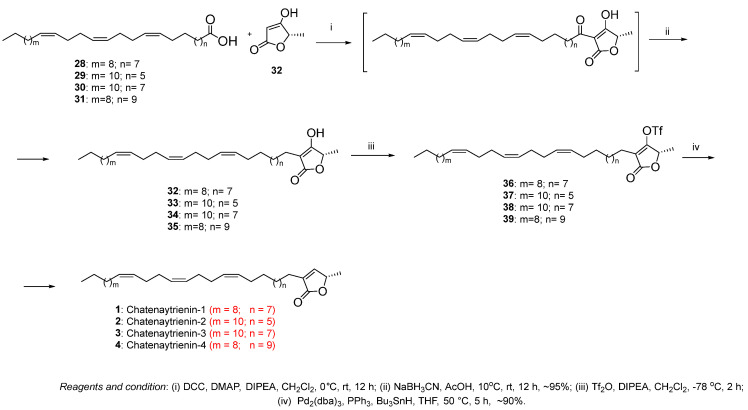
Fries rearrangement: introduction of the terminal α-substituted butenolide.

The final step in the synthesis of acetogenins implied the formation of a terminal butenolide fragment, carried out according to a well-established approach based on the application of the Fries rearrangement catalyzed by DMAP (Figure 4). Therefore, O-acylation of a cyclic β-keto ester (32) performed using a well-known procedure in two stages from (S)-ethyl lactate [29,30] with acids (28–31) followed by the Fries rearrangement initiated by dimethylaminopyridine (DMAP), produced intermediate reaction products, the reduction of which with NaBH3CN in acetic acid gave α-alkylated butenolides (32–35) in high yields of ~95%.

Elimination of the hydroxy group at the C-3 position of butenolides (32–35) was carried out by sequential synthesis of triflates (36–39) and their reduction with Bu3SnH catalyzed by Pd2(dba)3 to give the target chatenaytrienins-1, -2, -3 and -4 (1–4) in ~90% yield.

Thus, we have presented an original stereoselective 10-step method for the synthesis of natural chatenaytrienins-1, -2, -3 and -4 (1–4) involving a Ti-catalyzed reaction of cross-cyclomagnesiation of aliphatic and O-containing 1,2-dienes with a Grignard reagent. The proposed method offers huge synthetic potential as a convenient means for the stereoselective preparation of 1Z,5Z,9Z-triene systems.

### 3.2. Cytotoxicity Assay

The biological activity of acetogenins is of great interest to researchers working in the field of natural compounds. The reported data indicate that acetogenins are among the best dissipators of mitochondrial potential and modulate ATP production in cells [31,32]. Further, a mixture of compounds in extracts from the leaves of plants of the family Annonaceae was found to inhibit NADH-ubiquinone oxidoreductase, disrupting the oxidation of mitochondrial NADH by ubiquinone while disturbing the vectorial transfer of protons through the conjugating mitochondrial membrane [33]. Although acetogenins are inhibitors of tissue respiration through binding to complex I proteins, the detailed mechanisms of action of this class of compounds in mitochondria still need to be thoroughly studied. All currently known inhibitors of the respiratory chain in mitochondria, such as rotenone [34], pyericidin A [35], oligomycin [36], atractyloside [37], 2,4 dinitrophenol [38], CCCP (carbonyl cyanide-m-phenylhydrazone) [39], valinomycin [40], etc., have different mechanisms of action.

Recently, there have been many reports on the possible antitumor activity of acetogenins isolated as mixtures in aqueous and alcoholic extracts [41,42,43,44,45,46], but the biological activity of the pure compounds has not been specified yet. The main reason is labor intensity, the nontriviality of some types of synthesis of acetogenins, as well as unusually small amounts of the compounds obtained in multistage synthesis, which are insufficient for studying the biological activity [21]. In view of the above, we considered it necessary to synthesize and thoroughly study the biological activity of each of the four chatenaytrienins-1, -2, -3 and -4 (1–4) we synthesized by analyzing most of the signaling pathways for cell growth and proliferation. We also studied the genotoxicity, apoptosis induction, production of ROS ions in mitochondria, state of tissue respiration, as well as autophagy processes in order to detail the supposed mechanisms of action of acetogenins in living cells.

Chatenaytrienin-1 (1) was found to exhibit the greatest activity according to the cytotoxicity of these four compounds using six cell lines of different embryonic origin (Table 1).

Meanwhile, the sufficiently large scatter of the cytotoxicity values depending on the cell line indicated a certain selectivity of the compounds in relation to certain tumor cultures. Here, a pronounced difference in cytotoxicity between tumor culture lines, normal fibroblasts, and conditionally normal HEK293 cells was noteworthy. It should be taken into account that cytotoxicity depends on many factors, such as the properties of the cell culture, its embryonic origin, the methodology applied in determining the toxicity of a compound, and the detection methods used [47,48,49,50,51]. Chatenaytrienins-1, 2, 3 and -4 (1–4) were characterized by selective toxicity, i.e., for normal fibroblasts and conditionally normal cell line HEK293, the CC50 was almost five or six times greater than that for Jurkat or K562 cells (Table 1). This could be explained by the selective action of these compounds on tumor cultures with high division potential.

In the development of these studies and analysis of the literature containing data on acetogenins as mitochondrial agents, we studied the ability of these compounds to uncouple the processes of oxidation and phosphorylation, induce the formation of ROS ions, and initiate apoptosis through damage to mitochondria, i.e., initiate the mitochondrial pathway of cell death.

Mitochondria are important cellular organelles that provide the energy balance of the cell. They trigger processes such as apoptosis, necroptosis, and autophagy when destroyed or disrupted since they contain key regulators of cell death processes. Mitochondria are also key organelles in the production of ROS ions and predominate in the initiation of oxidative stress in cells. Thus, the functional significance of mitochondria is difficult to overestimate since these organelles are highly sensitive indicators of cell viability. The energy produced via the functioning of the respiratory chain accumulates in the form of an electrochemical gradient that determines the direction of movement of ions through the mitochondrial membrane, creating a mitochondrial transmembrane potential (ΔΨm) that enables the cell to control ATP synthesis [52]. Loss of the mitochondrial internal transmembrane potential often occurs [53,54], but not always [55], in connection with the phenomena of early apoptosis in the cell. The collapse of this potential has proven to be directly associated with the opening of the pores of the mitochondrial membrane. This promotes permeability, resulting in the release of cytochrome c into the cytosol, then triggering subsequent events in the apoptotic cascade. Potential changes in the mitochondrial membrane are involved in apoptosis, necrotic cell death, and caspase-independent cell death processes. Consequently, the processes of mitochondrial potential dissipation are a reliable indicator of mitochondrial dysfunction and cellular health that are essential in the study of various pathological conditions mediated by mitochondrial death [56].

Detection of mitochondrial potential dissipation using MitoSense Red, annexin V, and 7-AAD provided a simultaneous measurement of three important parameters of the cell state within the same cell sample: the state of the mitochondrial potential (Δψ), detected by the membrane-penetrating MitoSense Red dye; the expression of phosphatidylserine on the cell surface of apoptotic cells, assessed by the binding of annexin V to externalized phosphatidylserine; and the binding of 7-AAD to DNA in cells in the late stages of apoptosis. Multiparametric evaluation of these indicators of cell viability made it possible to determine the correlation and relationship between oxidation dissipation and phosphorylation with apoptosis.

**Figure 5:**
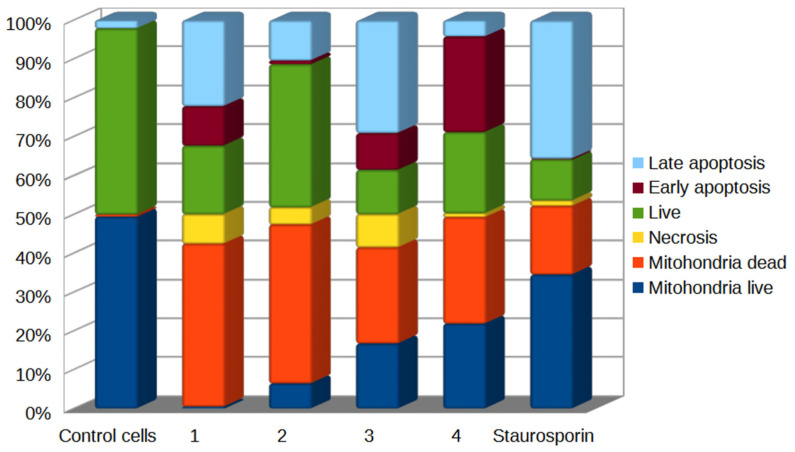
Detection of changes in the mitochondrial membrane potential (ΔΨ) and apoptosis in Jurkat cells treated with chatenaytrienins-1, -2, -3 and -4 (1–4), taken at a concentration of CC50. The cells were stained with MitoSense Red, annexin V (with CF488A), and 7-AAD (FlowCellect^®^ MitoDamage Kit). Incubation time was 4 h. Cytofluorometric analysis of the data from three independent experiments. Flow cytometry data in the form of rafts and histograms are presented in Supplementary Materials. The confidence intervals are given in Table S1 in Supplementary Materials.

**Figure 6:**
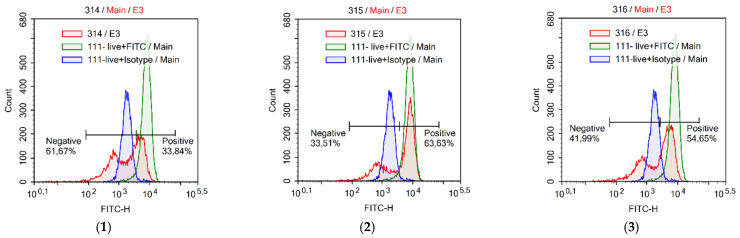

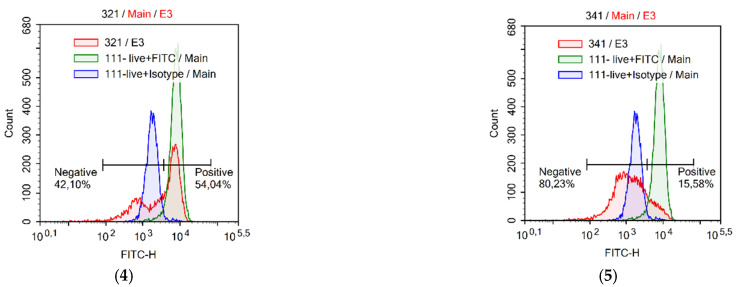
Detection of cytochrome c loss in Jurkat tumor cell line treated with chatenaytrienins-1, -2, -3 and -4 (1–4) taken at a concentration of CC50: (**1**) chatenaytrienin-1 (1); (**2**) chatenaytrienin-2 (2); (**3**) chatenaytrienin-3 (3); (**4**) chatenaytrienin-4 (4); and (**5**) staurosporine. Anti-cytochrome c-FITC antibody and Anti-IgG-FITC isotype control stain (FlowCellect^®^ Cytochrome c Kit). Incubation time was 3 h.

**Figure 7:**
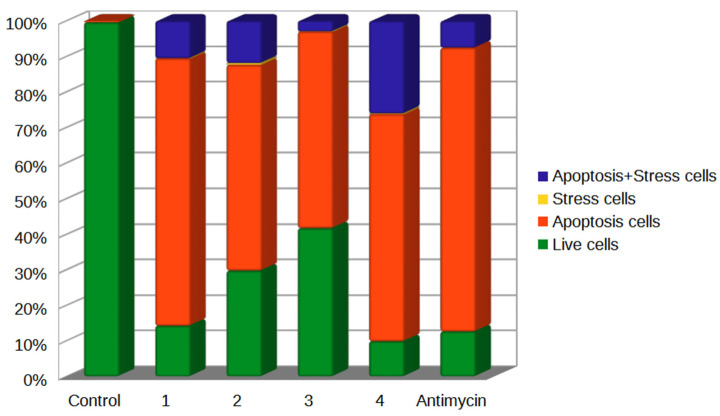
Detection of oxidative stress and early apoptosis events in Jurkat cells treated with chatenaytrienins-1, -2, -3 and -4 (1–4) taken at a concentration CC50 and concentration of antimycin is 100 µM. MitoSOX Red and annexin V stain, CF647 (FlowCellect™ MitoStress Kit). Incubation time was 4 h. Cytofluorometric analysis of the data from three independent experiments. Flow cytometry data in the form of rafts and histograms are in Supporting materials. The confidence intervals are given in Table S2 in Supplementary Materials.

When assessing the level of mitochondrial membrane potential (Δψ), a significant increase in the abundance of Jurkat tumor cells with the Δψ dissipation phenomenon was observed in the samples treated with chatenaytrienin-4 (4) (53.51%), chatenaytrienin-3 (3) (62.42%), chatenaytrienin-2 (2) (67.76%), and chatenaytrienin-1 (1) (83.69%) (Figure 5). The most pronounced decrease in the mitochondrial potential was caused by chatenaytrienin-1; the effect was dose dependent and exceeded that of the known inhibitor of most protein kinases and inducer of apoptosis in the cell, staurosporine (Figure 5). The percentages of cells in late apoptosis in the samples treated with chatenaitrienins-1, -2, -3 and -4 (1–4) were 15.11%, 8.44%, 17.01%, and 1.88%, respectively (Figure 5), while the number of cells stained with 7-AAD in the sample treated with staurosporine was 2.48%, which was comparable to the action of chatenaytrienin-4 (4). All other acetogenins under study turned out to be more active than staurosporine. Thus, chatenaytrienin-1 caused the highest percentage of mitochondrial damage, which was probably due to its chemical structure, namely the relative position of the triene system and the lactone cycle in the molecule. Obviously, further studies aimed at studying the structure–activity relationship as well as the precise definition of the molecular target will help to better understand the mechanisms of action of this class of acetogenins. Therefore, in order to obtain more reliable evidence of the mitochondrial mechanism of action of chatenaytrienins-1, -2, -3 and -4 (1–4), we studied the loss of cytochrome c (Figure 6) and activation of autophagy in cells (Figure 7) treated with the studied acetogenins.

The release of cytochrome c from the mitochondrial intermembrane space into the cytosol initiates allosteric activation and hepta-oligomerization of apoptosis protease activating factor 1 (Apaf-1), which generates a protein complex, the apoptosome. Each apoptosome recruits seven caspase-9 dimers and contributes to their activation, followed by proteolytic self-processing [57]. These processes are tightly regulated by several heat shock proteins (HSPs) and mediate catalytic maturation of caspase-3 and other caspases that ultimately mediate the biochemical and morphological features of apoptosis [58,59]. Once mitochondria become irreversibly permeable, cell death is believed to occur independently of caspase activity, although it can be delayed if caspases are not activated. This caspase-independent cell death may result from the loss of essential mitochondrial functions and/or the apoptogenic function of additional molecules that migrate from the IMS to the cytosol, namely apoptosis-inducing flavoprotein factor and endonuclease G. Once in the cytosol, both proteins are able to translocate to the nucleus, where they promote DNA fragmentation and apoptotic cell death in a caspase-independent manner [58]. Consequently, the release of cytochrome c into the cytoplasm and its detection can serve as a reliable marker of mitochondrial damage and activation of mitochondrial cell death. The cytometric plots in Figure 6 demonstrate the level of cytochrome c released into the cytoplasm in Jurkat cells after treatment with the compounds under study. Chatenaytrienin-1 (1) exhibited the highest percentage of cells with cytochrome c released into the cytoplasm (61.67%). This result was consistent with the effect of staurosporine (80.23%) (Figure 6). The remaining chatenaytrienins-2, -3 and -4 (2–4) had somewhat weaker effects (33.51%, 41.99%, and 42.10%, respectively). Thus, it is likely that chatenaytrienin-1 not only initiates apoptosis of the mitochondrial type due to damage to the mitochondrial membrane, but also promotes DNA fragmentation, which we will discuss in more detail later in this work.

To confirm the initiation of oxidative stress in cell mitochondria under treatment with chatenaytrienins-1, -2, -3 and -4 (1–4), we studied the level of formation of ROS ions in mitochondria under the action of MitoSOX™ Red dye, providing visualization of oxidative stress in mitochondria by flow cytometry. Superoxide formation occurs during oxidative phosphorylation and cellular respiration during the reduction of molecular oxygen in the electron transport chain [60]. Electron leakage taking place during adverse events in mitochondria induces the formation of superoxide. The superoxide anion is an incompletely reduced, very short-lived, but extremely reactive oxygen molecule [61]. The resulting superoxide anion can initiate a cascade of reactions that produce other ROS, including peroxynitrite, hydrogen peroxide, and the hydroxy radical. This vicious circle of oxidative stress and damage to cellular structures results in cell death, apoptosis, or a decrease in cell energy processes and aging [62]. In general, detection of ROS ions and oxidative stress in mitochondria is a rather time consuming and difficult issue [63,64]. The use of fluorescent probes (such as MitoSOX Red) as well as a fairly large pool of cells (up to 100,000 events) can be analyzed by flow cytometry, ensuring an accurate record of oxidative stress events in the cells under study. Figure 7 shows histograms of oxidative stress detection in Jurkat tumor cells treated with the synthesized acetogenins.

Mitochondrial regulation of apoptosis and oxidative stress are closely related, as repeatedly reported in the literature [65,66,67], and the correlation between oxidative stress and apoptosis has been demonstrated. Therefore, simultaneous detection of mitochondrial superoxide generation (detected by MitoSOX Red membrane-penetrating dye) and expression of phosphatidylserine on the cell surface of apoptotic cells (assessed by binding of annexin V) in the same cell sample provides an accurate evaluation of the ability of the test compound to initiate oxidative stress in cell culture and induce apoptosis.

Under the influence of chatenaytrienins-1, -2, -3 and -4 (1–4), the processes of activation of oxidative processes in mitochondria, as well as the entry of cells into apoptosis, were observed. For example, chatenaytrienin-1 (1) worked most rapidly and actively (75% of cells were in early apoptosis, and 10.53% demonstrated oxidative stress along with the marker of early apoptosis, annexin). This action was comparable to antimycin (Figure 7). Chatenaytrienin-3 (3) left the smallest percentage (only 9.9%) of intact (live) cells in the sample, while antimycin left 12.65%. The percentages of cells simultaneously demonstrating markers of stress and apoptosis in the samples were also noteworthy. The samples treated with chatenaytrienins-2 (2) and -4 (4) after 4 h of incubation exhibited the highest percentages of such cells, that is, 11.86% and 26.8%, respectively.

Thus, we can assert that chatenaytrienins-1, -2, -3 and -4 (1–4) are able to easily penetrate the mitochondrial membrane, uncouple oxidation and phosphorylation, cause intramitochondrial stress, and initiate apoptosis via the mitochondrial pathway. This conclusion was confirmed by an early study of the biological activity of acetogenins [68]. Londerhausen et al. initially observed that the toxicity induced by annonaceous acetogenins resulted in lethargy in insects and reduced their mobility before death; acetogenin-treated insects had significantly lower levels of adenosine triphosphate (ATP), similar to the effect of antimycin A, a known inhibitor of the mitochondrial electron transport system (ETS). When testing respiratory depression in mitochondria through the inhibition of mitochondrial enzymes, the mixture of acetogenins turned out to be 2.5–5 times more effective than rotenone in inhibiting complex I (NADH: ubiquinone oxidoreductase) [69].

In addition to apoptosis, it was of interest for our group to determine whether treatment of cells with chatenaytrienins induced autophagy. Autophagy is an intracellular catabolic pathway responsible for the turnover of cellular proteins and organelles and is closely associated with various pathological processes, such as Alzheimer’s disease, cancer, aging, and autoimmune diseases. It is a highly regulated process that plays a key role in growth, development, and cellular homeostasis. The main function of autophagy as a housekeeping mechanism is to get rid of senescent and/or dysfunctional proteins and organelles through sequestration and preparation of such proteins for lysosomal degradation. The research data in recent years have confirmed that not only apoptosis but also autophagy can contribute to the death of cell populations and greatly affect the lifespan and general mechanisms of cells [70]. On the one hand, autophagy can induce a certain autophage-type cell death after damage and stress, and, on the other hand, it can be a mechanism for restoring their viability. This means that autophagy can be cytoprotective or a death mechanism depending on the conditions [71].

Canonical autophagy is associated with the conversion of LC3 protein (an autophagosome marker) from the cytoplasmic form of LC3I to the lipid-bound form of LC3II, which is associated with the membranes of maturing autophagosomes [72]. The autophagy process can be conveniently classified into several stages. First, induction and translocation of the cytoplasmic LC3 protein occur due to the influence of external stimuli (adverse environmental influences)—the process is initiated by external/internal stimuli (for example, nutrient depletion or hypoxia). Then, the formation of autophagosomes begins, which are double-membrane vesicles that include the LC3 protein. This protein regulates the fusion of autophagosomes and lysosomes. It exists in two fractions—cytoplasmic LC3-I and LC3-II, isolated in the autophagosomal membrane. The LC3-II protein fraction was specified in our work, as its detection is a reliable indicator of the processes of autophagy in the cell [72,73].

**Figure 8:**
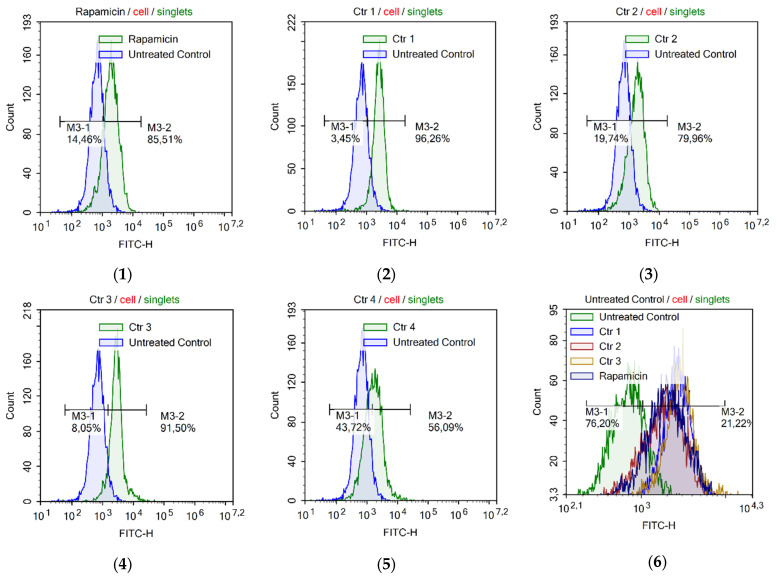
Induction of autophagy in Jurkat cells upon incubation with (**1**) rapamycin (0.8 µM); (**2**) chatenaytrienin-1 (1) at a concentration of 0.5 CC_50_; (**3**) chatenaytrienin-2 (2) at a concentration of 0.5 CC_50_; (**4**) chatenaytrienin-3 (3) at a concentration of 0.5 CC_50_; (**5**) chatenaytrienin-4 (4) at a concentration of 0.5 CC_50_; (**6**) chatenaytrienins-1, -2, -3, and -4 (1–4) at a concentration of 0.5 CC_50_ and rapamycin (0.8 µM) compared to normal cells. Anti-LC3/FITC antibody stain (Autophagy LC3 Antibody-based Assay Kit). Incubation was 48 h.

The analysis of the actions of rapamycin and chatenaytrienins-1, -2, -3 and -4 (1–4) (Figure 8) revealed that the action of chatenaytrienins was very similar to that of rapamycin, an mTOR kinase inhibitor and autophagy activator [74,75,76]. Each of the six histograms represents an intact control and cells treated with the test compounds. The highest numbers of cells with autophagy were observed in the samples treated with chatenaytrienins-1 (1) and -3 (3) (96.26% and 91.50%, respectively). Chatenaytrienins-2 (2) (79.96%) and -4 (4) (56.09%) induced lesser degrees of autophagy. It is rather difficult to explain the rate of autophagy in the cells due to the effect of the compounds studied or the peculiarities of their structures. Autophagy is a process that exists in the cell under both normal and pathological conditions, and possibly, in the case of exposure to chatenaytrienins, it was activated in response to hypoxic stress. This is a very likely but approximate mechanism of action of the compounds studied. In order to understand more deeply how acetogenins act in the cell, it will probably be necessary to analyze the proteome and try to determine the exact molecular target. Evidently, one of the neuronal damaging mechanisms subsequently leading to progressive supranuclear palsy (PSP) and atypical parkinsonism found in the local residents of certain areas of Central and South America [77] is the long-term, chronic, damaging exposure of respiratory processes in mitochondria to acetogenins and increased levels of autophagy in nervous tissue. This eventually results in atypical forms of neurodegenerative diseases related to the consumption of various parts of plants of the family Annonaceae.

Some research papers have mentioned the possible genotoxicity of acetogenins. Still, the study of pure acetogenins has been reported in few works to date [78] and not their mixtures [79].

**Figure 9:**
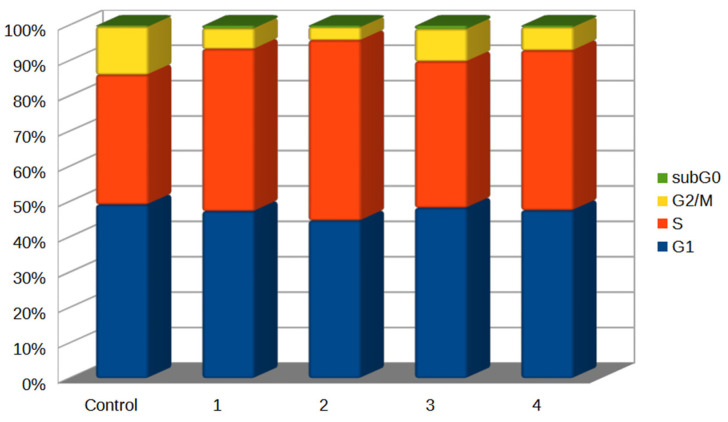
Histograms of cytofluorometric analysis of cell cycle phases in Jurkat cells under the influence of the studied acetogenins. The cells were treated with chatenaytrienins-1, -2, -3 and -4 (1–4) at CC_50_. Guava^®^ Cell Cycle Reagent stain. The time of incubation of compounds with cells was 48 h. All results of cytofluorometric analysis were obtained from at least three independent experiments. Flow cytometry data in the form of rafts and histograms are presented in Supplementary Materials. The confidence intervals are given in Table S3 in Supplementary Materials.

A number of scientific sources indicated the high antitumor activity of various acetogenins by influencing the cell cycle. Many acetogenins supposedly regulate the cell cycle to the G1/S transition checkpoint by inhibiting cyclin D1 expression in human hepatocellular carcinoma cells [80,81]. In one of our experiments, a traditionally used extract of *A. muricata* arrested the cell cycle in the G1 phase and reduced the number of cells in the S phase in a concentration-dependent manner by reducing the expression of cyclin D1, an important regulatory protein of the cell cycle [82]. A similar result was observed for squamocin, which blocked cells in the G1 phase in T24 bladder cancer cells [83]. Despite the relevance of a detailed study of the mechanism of cell cycle inhibition by acetogenins, this issue has been poorly covered in the literature so far. In our work, we tested the effect of all the synthesized chatenaytrienins-1, -2, -3 and -4 (1–4) on the cell cycle (Figure 9).

The cell cycle parameters in the control sample showed a significant predominance of cells in the G0-G1 phase and a balance between the processes of synthesis (S-phase) and apoptosis (sub-G0-G1 interval) (Figure 9).

After 48 h of exposure to the compounds under study, cell processes in the form of a slightly increased ability of cells to synthesize DNA (S-phase) dominated in almost all samples (Figure 9). Meanwhile, the sub-population of sub-G0-G1 cells was insignificantly expressed in all of the tested samples and practically did not differ when comparing the control sample and samples with exposure to chatenaytrienins-1, -2, -3 and -4 (1–4). Typically, the sub-G0-G1 population characterizes cells that have undergone apoptosis and contain fragmented DNA that differs sharply from the diploid state of the genome of whole living cells in the G1-G0 phase. If apoptosis proceeds along the mitochondrial pathway without affecting DNA and causing pronounced chromatin degradation, then it is obvious that the cell population in the sub-G0-G1 phase will be minimal.

The highest percentage of cells in the S-phase (~47%) was registered in the samples treated with chatenaytrienin-2 (2) (Figure 9). For comparison, the percentage of cells in the S-phase did not exceed 34.2% in the control sample. Meanwhile, almost all of the samples exhibited an equal number of cells in the G0-G1 phase, along with an increase in the proliferation block and a decrease in the proliferation index due to a decrease in the number of cells in the G2+M phase. All of the above results obviously indicated the cytotoxicity of chatenaytrienins-1, -2, -3 and -4 (1–4) towards T cell leukemia cells due to the ability of this group of substances to induce apoptosis via the mitochondrial pathway. In this regard, the issue of the genotoxicity of these compounds remains a challenging issue, since there are a lot of reported data on the effect of acetogenins on the genome of neurons and their neurotoxicity. However, almost all of the studies of this kind involved mixtures or plant extracts with various acetogenins [84,85,86,87].

For the first time, our group tested this individual class of compounds of acetogenin for genotoxicity and studied the activation of a number of signaling pathways for cell growth and viability by determining the level of phosphorylated intracellular signaling kinases, including p53, MDM2, p21, p38, PI3K/AKT/mTOR, Bcl-2/Bax, STAT3 and STAT5, Nf-kB, Caspase 3-8-9, CREB, ERK/MAP, p70 S6, JNK, ATM, Chk1, Chk2, and H2AX. The changes in the expression and activation of many of these proteins were typical of the tumor process, since some of the kinases under study are responsible for a variety of processes in the body, such as inflammation, growth, proliferation, and tumor transformation, and are also markers of genotoxicity, initiating repair processes in the cell in response to adverse environmental influences [88,89,90,91,92]. Phosphorylation is one of the main mechanisms of signal transduction and regulation of various processes essential for cell life, namely differentiation, growth, proliferation, apoptosis, etc. [93]. Changes in the operation of a signaling or regulatory cascade, accompanied by changes in the level of protein phosphorylation, can result in cell death, growth arrest, development of cancer or neurodegenerative diseases, and also serve to transmit signals in response to various extracellular stimuli [94,95,96]. The study of the profile of phosphorylated proteins often provides relevant information on the molecular mechanisms involved in the development of various diseases [97,98]. Another acetogenin synthesized and studied by our group earlier, muricadienin, was included in the experiment to achieve a more accurate and deeper understanding of the mechanisms of action of acetogenins in the cell [23]. Chatenaytrienin-1 (1) was chosen for the analysis of protein signaling because it had the highest cytotoxicity.

The levels of phosphorylated and non-phosphorylated proteins were analyzed using MILLIPLEX^®^ map technology to study the analytes in the same sample within a fairly short time. Various deviations in protein concentration due to external factors or detection errors that could affect the results of the experiment were thereby reduced to zero.

The expression of p53, MDM2, p21, p38, PI3K/AKT/mTOR, Bcl-2/Bax, STAT3 and STAT5, Nf-kB, Caspase 3-8-9, CREB, ERK/MAP, p70 S6, JNK, ATM, Chk1, Chk2, and H2AX proteins in lysate samples of Jurkat tumor cells were studied using Luminex technology with the MILLIPLEX^®^ MAP 9-Plex Multi-Pathway 9-plex Magnetic Bead Kit. This bead-based assay involved color-coded fluorescent particles pre-coated with specific antibodies targeting 9 major kinases in signaling pathways.

**Figure 10:**
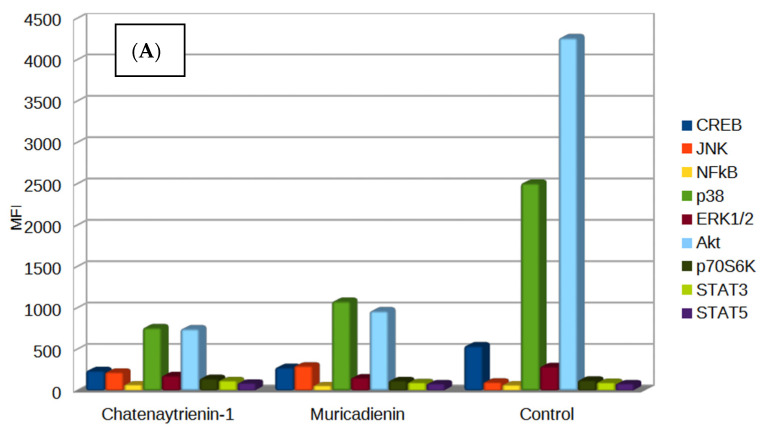

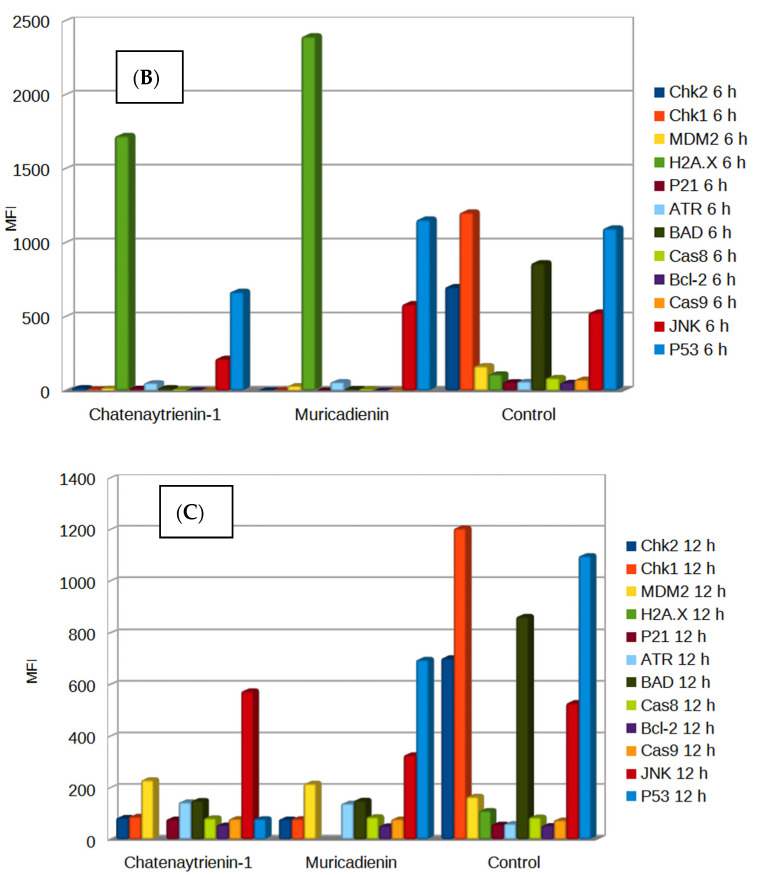
Histograms of the main signaling pathways in Jurkat tumor line cells treated with muricadienin (Mu5_2) and chatenaytrienin-1 (1) (MT1_1) and in intact cells. (**A**) Phosphorylated proteins CREB, ERK/MAP, p70 S6, p21, p38, PI3K/AKT/mTOR, and JNK; (**B**,**C**) p53, MDM2, Bcl-2/Bax, STAT3 and STAT5, Nf-kB, Caspase 3-8- 9, JNK, ATM, Chk1, Chk2, and H2AX. Incubation times were (**A**) 4 h, (**B**) 6 h, and (**C**) 12 h. The data on the Y-axis are given in MFI units. The data represent values obtained in triplicate. The 95% confidence intervals are provided in parentheses in Supplementary Materials.

In general, the comparison of phosphorylated and unphosphorylated fractions of CREB, JNK, NFkB, p38, ERK1/2, Akt, p70S6K, STAT3, and STAT5 proteins revealed that the main changes in the ratios of these two fractions were expressed for all of these nine signaling pathways (Figure 10). Signaling via RTKs (receptor tyrosine kinases) often increases metabolism and cell growth. Moreover, receptor tyrosine kinases are known not only as key regulators of normal cellular processes but also for playing critical roles in the development and progression of many types of malignant tumors [99]. ERK/MAP and Akt are two key families of Ser/Thr kinases activated via tyrosine kinase receptors, inducing activation of p70S6, Msk1, STAT3 (Ser727), and CREB kinases, which in turn increase the activation of other intermediates. Signaling pathways induced by stress or the FAS receptor (death receptor) cause activation of p38, JNK, and NF-κB. Chatenaytrienin-1 (1) at a concentration of CC_50_ (MT1_1) as well as muricadienin (CC_50_) most pronouncedly reduced the levels of all types of kinase proteins in the tumor cell. Under the influence of chatenaytrienin-1 (1), levels of Akt, p38, and CREB were most pronouncedly suppressed. Akt is an intracellular enzyme, one of three members of the protein kinase B family. Akt kinase is a key enzyme of the PI3K/AKT signaling pathway involved in the regulation of cell proliferation, growth, and survival [100]. Currently, considerable attention is paid to the study of the functions of this enzyme since it acts as an oncogene in many malignant diseases [101].

P38 refers to MAPK kinases (mitogen-activated protein kinases), which are serine-threonine protein kinases. They are activated in response to numerous external influences and transmit signals from the cell surface to the cell nucleus. MAPK kinases are a central component of the Ras/ERK/MAPK signaling cascade responsible for cell growth and differentiation. Proteins of the Ras and Raf families are important prognostic markers of tumor diseases and targets for therapeutic effect [102].

CREBs are widely expressed transcription factor binding cis-regulatory elements (CREs) found in the genomic regulatory regions of many neurotransmitter and growth factor responsive genes. CREB activity is regulated by the phosphorylation of serine residue Ser133. Phosphorylation of CREB under cell stimulation is one of the key determinants of whether a stimulus can activate the transcription of CREB-mediated genes. After Ser133 phosphorylation, CREB attracts the co-activator CBP (CREB-binding protein) and p300, which has histone acetyl transferase activity. After their recruitment to the promoter, CBP and p300 provide transcription initiation by direct interaction with the components of the mechanism of basic transcription factors, as well as through histone acetylation. Histone acetylation decondenses chromatin and provides access to major transcription factors in the core of the promoter region. Owing to phosphorylation, CREB is a key regulator of the processes driven by stimulus-dependent gene expression.

Hence, inhibition of all of the kinases of the nine signaling pathways under study by synthesized chatenaytrienin-1 (1) had an obvious negative effect on the growth and proliferative activity of tumor cells, thereby proving it to be an effective antitumor agent and a promising candidate for further in vivo studies as an antitumor chemotherapeutic agent.

In some human tumors, MDM2 has been shown to be abnormally upregulated due to gene amplification and increased transcription and translation [103], resulting in further degradation and decreased activity of p53. Therefore, the MDM2-p53 protein complex offers a promising therapeutic strategy for p53 reactivation during oncological transformation in tissues [104].

The cellular response to DNA damage is mainly coordinated by two different kinase signaling pathways, ATM-Chk2 and ATR-Chk1, activated by double-stranded and single-stranded DNA breaks, respectively. These pathways were previously considered to operate in parallel with overlapping functions. However, not long ago it became apparent that their relationship was more complex. In response to double-stranded DNA damage, ATM is required both to activate ATR-Chk1 and to initiate DNA repair through homologous recombination (HRR), promoting the formation of single-stranded DNA at damage sites via nucleolytic resection. It is noteworthy that cells and organisms survive with mutations in ATM or other components required for HRR, such as BRCA1 and BRCA2, but at the expense of genomic instability and cancer predisposition. In contrast, the ATR-Chk1 pathway is a major direct effector of DNA damage and replication checkpoints required for the survival of many but not all cell types. HRR deficiency in BRCA1- and BRCA2-deficient tumors remarkably confers sensitivity to cisplatin and inhibitors of poly(ADP-ribose)polymerase (PARP), an enzyme required to repair damaged DNA. Furthermore, suppression of DNA damage and replication checkpoint responses by Chk1 inhibition may enhance tumor cell killing by various genotoxic agents [105].

Figure 10 demonstrates that, in comparison with the protein profile of the control sample, almost all major proteins were suppressed in the first 6 h of incubation, with the exception of the phosphorylated fraction of H2AX histone. An increase in the H2AX level in the samples treated with the test compounds was observed with increasing incubation time, indicating the presence of double-strand breaks in DNA and the subsequent genotoxicity of the test substances. The DNA changes were either secondary and occurred due to the processes of apoptosis and disruption of the functioning of the respiratory chain, initiated by the studied acetogenins in cells, or these two processes, mitochondrial type apoptosis and accumulation of double-strand breaks in the cell genome, occurred in parallel and independently. Still, due to the low activity of p53 in the Jurkat cell line, apoptosis initiated by DNA double-strand breaks occurred much later than the destruction of mitochondria.

Many chemotherapeutic agents, such as etoposide and camptothecin as well as their derivatives, damage tumor cells by inducing DNA double-strand breaks [106]. The H2A.X protein is a member of the H2A histone family. Moreover, the level of γ-H2A.X detected by flow cytometry was shown to correlate with the number of DNA strand breaks and tumor cell death. The process of serine phosphorylation at position 139 of the H2A.X protein is a reliable indicator of DNA damage and obvious genotoxicity of the compound [107,108,109]. As the level of DNA damage increases, the level of phosphorylated H2A.X increases, accumulating specifically at the sites of damage to the DNA molecule. It is the accumulation of phosphorylated H2A.X that is often used as a marker of the level of DNA damage inside the cell as it plays an important role in DNA repair processes [110,111].

Cellular apoptosis is a complex biological process associated with the activation of numerous signaling pathways, and certain proteins are activated in every case, which can be targeted markers of some intracellular processes; their imbalance brings about the activation of programmed cell death. The activation of cysteine proteases, caspases in particular, is a key intracellular regulator of cell apoptosis, while caspases are involved in both internal and external pathways of apoptosis [112]. Caspase-3 is an important mediator of apoptosis [113], promoted by various activators classified into two main signaling pathways: the death receptor-mediated pathway, involving caspases-8 and -10, and the mitochondria-mediated pathway, involving caspase-9 [114]. Caspase-3 is the main protease activated by both FAS receptor ligands and cellular apoptosis induced by mitochondrial dysfunction [115]. The tumor suppressor protein p53 is a positive regulator of the pro-apoptotic proteins Bax, Bad, and Bak to prevent Bcl-2 uptake. Free Bax, Bad, and Bak subsequently bind to the mitochondrial membrane, bringing about mitochondrial damage and cell apoptosis [116]. Previous studies have shown that p53 promotes the transcription of Bax and Bad, which regulate the release of cytochrome c from mitochondria and lead to cell apoptosis by activating the cleavage of caspases-3 and -9 [117]. At present, Bcl-2 and Bax proteins are known to be in a state of constant dynamic equilibrium, forming homo- and heterodimers. The latter do not have proapoptogenic activity. When the production of proapoptotic Bax protein is dominant, this balance is disturbed and shifts towards the formation of a large number of homodimers with high proapoptogenic activity [118]. In our project, we were primarily interested in the molecular mechanisms of action of acetogenins in the cell, as well as their possible genotoxicity and ability to induce apoptosis. In this study, quite similar molecular profile patterns of chatenaytrienins and muricadienin were found throughout the entire incubation time. It should be noted that the concentration of p53 was significantly increased in the sample treated with muricadienin after 12 h of incubation, while the levels of this protein in all of the other samples were almost similar to that in the control. Treatment with muricadienin as well as with chatenaytrienin-1 (**1**) did not significantly affect the expression of caspase-8, but it promoted the accumulation of cleaved caspase-9. This was especially evident in the study of cell samples after 12 h of incubation, where the level of caspase-9 in the samples treated with muricadienin and chatenaytrienin-1 (**1**) exceeded the value of this protein in the control sample, supporting the fact that chatenaytrienins, like other acetogenins, selectively induce apoptosis in cells through a mitochondria-mediated pathway. This fact was confirmed by the study of the biogenesis of mitochondrial potential by means of flow cytometry carried out in this study. Further, we found that the level of BAD protein decreased under the influence of acetogenins (muricadienin and chatenaytrienin-1 (**1**)) in the first 6 h, and then within 12 h of incubation, the level of BAD protein again began to increase but did not reach the control value. In contrast, Bcl-2 remained downregulated in Jurkat cells after treatment with acetogenins. These results suggest that acetogenins can activate p53 through a downstream target protein that causes mitochondrial dysfunction by promoting cytochrome c release, thereby inducing cell apoptosis via the intrinsic mitochondrial pathway. 

## 4. Conclusions

Thus, the present research paper details the molecular mechanisms of action of the synthesized chatenaytrienins in the tumor cell, including comparison of the protein profiles of two different acetogenins, muricadienin and chatenaytrienin-1. Both acetogenins exhibited pronounced antitumor activity, effectively suppressing the protein pathways responsible for cell growth and proliferation, while the main mechanism of action of acetogenins is not only their influence on mitochondria but also a pronounced genotoxic effect due to the accumulation of DNA double-strand breaks. Meanwhile, the most likely mechanism of DNA damage, evidenced by a high level of phosphorylated H2A.X histones, is a high level of ROS ions in the cell in response to the action of acetogenins. Moreover, we managed to show that concentration of ROS ions in the cell began to increase literally in the first hours after contact with chatenaytrienins. Still, there are a number of issues concerning some aspects of the interaction between acetogenins and the respiratory chain, namely which protein component is the main target of acetogenins and whether these compounds can be considered terratogens.

## Figures and Tables

**Table 1:** Cytotoxic Activity of Synthesized Natural Chatenaytrienins-1, -2, -3 and -4 (1–4) in Tumor Cell Lines (Jurkat, K562, U937, HL60), a Conditionally Normal Line (Hek293), and Normal Fibroblasts (CC_50_, µM).

	Jurkat	K562	U937	HL60	Hek293	Fibroblasts (PCS-201-018)
**1**	0.09 ± 0.02 (0.08–0.99)	0.10 ± 0.03 (0.09-0.11)	0.09 ± 0.02 (0.08–0.11)	0.15 ± 0.01 (0.14–0.16)	0.53 ± 0.02 (0.51–0.54)	0.69 ± 0.04 (0.67–0.81)
**2**	0.12 ± 0.06 (0.11–0.13)	0.18 ± 0.05 (0.17–0.19)	0.12 ± 0.01 (0.11–0.13)	0.13 ± 0.02 (0.12–0.14)	0.63 ± 0.03 (0.61–0.64)	0.79 ± 0.02 (0.77–0.82)
**3**	0.15 ± 0.04 (0.14–0.16)	0.09 ± 0.01 (0.08–0.1)	0.18 ± 0.02 (0.17–0.19)	0.14 ± 0.04 (0.13–0.15)	0.78 ± 0.04 (0.76–0.79)	0.83 ± 0.05 (0.81–0.84)
**4**	0.16 ± 0.01 (0.15–0.17)	0.13 ± 0.04 (0.12–0.14)	0.13 ± 0.05 (0.12–0.14)	0.19 ± 0.02 (0.18–0.21)	0.53 ± 0.01 (0.52–0.54)	0.98 ± 0.04 (0.96–0.99)

95% confidence intervals are provided in parenthesis. A standard error of the mean is provided. CC50, half-maximal cytotoxicity concentration.

## Data Availability

The data presented in this study are available in this article.

## Supplementary Materials

The following supporting information can be downloaded at: https://www.mdpi.com/article/10.3390/antiox12081528/s1.
