# Supplementary material for: Natural Acetogenins, Chatenaytrienins-1, -2, -3 and -4, Mitochondrial Potential Uncouplers and Autophagy Inducers—Promising Anticancer Agents
Source: Antioxidants (Basel). 2023 Jul 30;12(8):1528. doi: 10.3390/antiox12081528 (PMC10451668; doi:10.3390/antiox12081528)
Supplement: Supplementary file 1 [file antioxidants-12-01528-s001.zip › antioxidants-2497744-supplementary.pdf]

Supporting information for

Natural acetogenins, chatenaitrienins-1, -2, -3 and -4 - mito-chondrial potential uncouplers and autophagy inducers - promising anticancer agents

Lilya U. Dzhemileva *, Regina A. Tuktarova, Usein M. Dzhemilev, and Vladimir A. D'yakonov *

N.D. Zelinsky Institute of Organic Chemistry, Russian Academy of Sciences, Leninsky Prospect 47, Moscow 119991, Russia; regina-tuktarova@yandex.ru (R.A.T.); dzhemilev@anrb.ru (U.M.D.)

** Correspondence: dzhemileval@ioc.ac.ru (L.U.D.); dyakonovva@ioc.ac.ru (V.A.D.)*

Table of contents

1. ^{1}H and ^{13}C NMR Spectra of the compounds **25–27**, **29–31**, **33–35**, **37–39** S3

2. Figures for spectrum of the compounds **25–27**, **29–31**, **33–35**, **37–39** S10

^{1}H and ^{13}C NMR Spectra of the compounds 25–27, 29–31, 33–35, 37–39

2-[(9Z,13Z,17Z)-Triaconta-9,13,17-trien-1-yloxy]tetrahydro-2*H*-pyran (25)

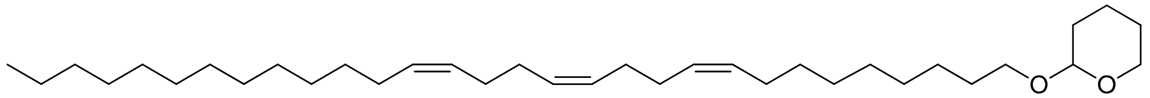

Pale yellow oily liquid; ^{1}H NMR (500 MHz, $CDCl_3$): 5.44–5.35 (m, 6H), 4.59 (m, 1H), 3.88 (m, 1H), 3.75 (m, 1H), 3.50 (m, 1H), 3.40 (m, 1H), 2.12–2.01 (m, 12H), 1.86 (m, 1H), 1.73 (m, 1H), 1.63–1.52 (m, 6H), 1.40–1.25 (m, 30H), 0.89 (t, J = 6.5 Hz, 3H); ^{13}C NMR (125 MHz, $CDCl_3$): 130.4, 130.3, 129.6 (signals of 2C), 129.1 (signals of 2C), 98.8, 67.7, 62.3, 31.9, 30.8, 29.8–29.3 (signals of 13C), 27.5 (signals of 2C), 27.4 (signals of 2C), 27.3 (signals of 2C), 26.3, 25.5, 22.7, 19.7, 14.1; IR (film): 3008, 2956, 2924, 2853, 1619, 1465, 1352, 1273, 1201, 1137, 1120, 1079, 1034, 970, 908, 869, 815, 722 cm^{-1}; Analysis calculated for $C_{35}H_{64}O_2$: C, 81.33; H, 12.48. Found: C, 81.21; H, 12.45. MALDI TOF: m/z 539.509 ([M+Na]$^+$, calcd 539.491).

2-[(11Z,15Z,19Z)-Dotriaconta-11,15,19-trien-1-yloxy]tetrahydro-2*H*-pyran (26)

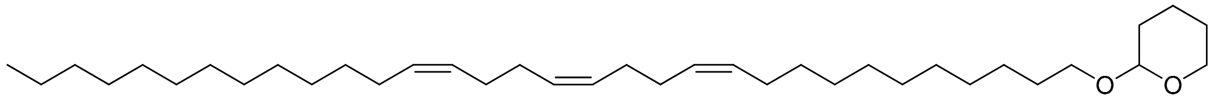

Pale yellow oily liquid; ^{1}H NMR (500 MHz, $CDCl_3$): 5.44–5.35 (m, 6H), 4.59 (m, 1H), 3.88 (m, 1H), 3.74 (m, 1H), 3.51 (m, 1H), 3.39 (m, 1H), 2.13–2.00 (m, 12H), 1.85 (m, 1H), 1.72 (m, 1H), 1.63–1.52 (m, 6H), 1.40–1.25 (m, 34H), 0.89 (t, J = 6.5 Hz, 3H); ^{13}C NMR (125 MHz, $CDCl_3$): 130.4 (signals of 2C), 129.6 (signals of 2C), 129.1 (signals of 2C), 98.8, 67.7, 62.3, 31.9, 30.8, 29.8–29.4 (signals of 15C), 27.5 (signals of 2C), 27.4 (signals of 2C), 27.3 (signals of 2C), 26.3, 25.5, 22.7, 19.7, 14.1; IR (film): 3007, 2925, 2854, 1621, 1464, 1377, 1353, 1271, 1136, 1122, 1035, 970, 907, 869, 815, 721 cm^{-1}; Analysis calculated for $C_{37}H_{68}O_2$: C, 81.55; H, 12.58. Found: C, 81.38; H, 12.55. MALDI TOF: m/z 567.479 ([M+Na]$^+$, calcd 567.512), 583.503 ([M+K]$^+$, calcd 583.486).

2-[(13Z,17Z,21Z)-Dotriaconta-13,17,21-trien-1-yloxy]tetrahydro-2*H*-pyran (27)

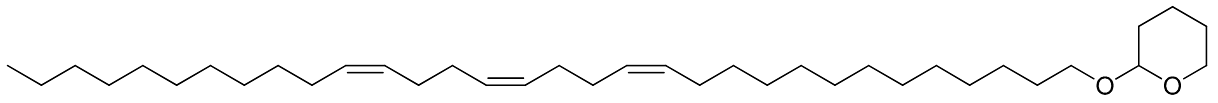

Pale yellow oily liquid; ^{1}H NMR (500 MHz, $CDCl_3$): 5.44–5.34 (m, 6H), 4.59 (m, 1H), 3.88 (m, 1H), 3.76 (m, 1H), 3.52 (m, 1H), 3.39 (m, 1H), 2.13–1.98 (m, 12H), 1.86 (m, 1H), 1.73 (m, 1H), 1.64–1.52 (m, 6H), 1.40–1.23 (m, 34H), 0.91 (t, J = 6.5 Hz, 3H); ^{13}C NMR (125 MHz, $CDCl_3$): 130.4 (signals of 2C), 129.6 (signals of 2C), 129.1 (signals of 2C), 98.8, 67.7, 62.3, 31.9, 30.8, 29.8–29.3 (signals of 15C), 27.5 (signals of 2C), 27.4 (signals of 2C), 27.3 (signals of 2C), 26.3, 25.5, 22.7, 19.7, 14.1; IR (film): 3007, 2925, 2854, 1621, 1464, 1377, 1353, 1271, 1136, 1122, 1035, 970, 907, 869, 815, 721 cm^{-1}; Analysis calculated for $C_{37}H_{68}O_2$: C, 81.55; H, 12.58. Found: C, 81.37; H, 12.56. MALDI TOF: m/z 567.466 ([M+Na]$^+$, calcd 567.512).

(9Z,13Z,17Z)-Triaconta-9,13,17-trienoic acid (29)

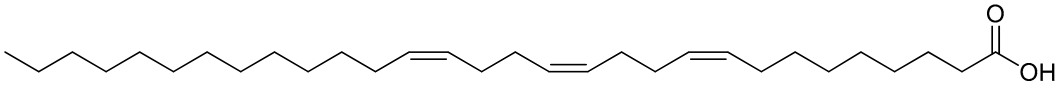

Colorless oil; ^{1}H NMR (400 MHz, $CDCl_3$): 5.45–5.35 (m, 6H), 2.37 (t, J = 7.6 Hz, 2H), 2.13–2.02 (m, 12H), 1.65 (m, 2H), 1.40–1.25 (m, 28H), 0.91 (t, J = 6.4 Hz, 3H); ^{13}C NMR (100 MHz, $CDCl_3$): 179.9, 130.4, 130.3, 129.7, 129.6, 129.2, 129.1, 34.0, 31.9, 29.8–29.0 (signals of 12C), 27.4 (signals of 2C), 27.3 (signals of 2C), 27.2 (signals of 2C), 24.7, 22.7, 14.1; IR (film): 3006, 2924, 2854, 1711, 1464, 1378, 1285, 1248, 1087, 966, 940, 723 cm^{-1}; Analysis calculated for $C_{30}H_{54}O_2$: C, 80.65; H, 12.18. Found: C, 80.49; H, 12.16. MALDI TOF: m/z 469.435 ([M+Na]$^+$, calcd 469.402), 485.408 ([M+K]$^+$, calcd 485.376).

(11Z,15Z,19Z)-Triaconta-11,15,19-trienoic acid (30)

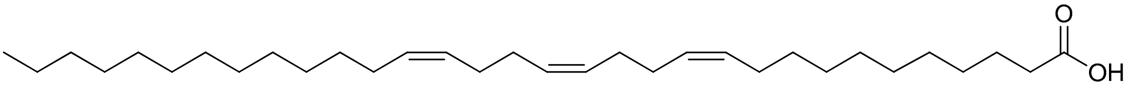

Colorless oil; ^{1}H NMR (400 MHz, $CDCl_3$): 5.45–5.35 (m, 6H), 2.37 (t, J = 7.2 Hz, 2H), 2.13–2.02 (m, 12H), 1.65 (m, 2H), 1.40–1.26 (m, 32H), 0.91 (t, J = 6.4 Hz, 3H); ^{13}C NMR (100 MHz, $CDCl_3$): 180.2, 130.4, 130.3, 129.6 (signals of 2C), 129.1 (signals of 2C), 34.1, 31.9, 29.7–29.1 (signals of 14C), 27.5 (signals of 2C), 27.4 (signals of 2C), 27.3 (signals of 2C), 24.7, 22.7, 14.1; IR (film): 3006, 2925, 2854, 1712, 1465, 1377, 1285, 1243, 1086, 963, 941, 722 cm^{-1}; Analysis calculated for $C_{32}H_{58}O_2$: C, 80.95; H, 12.31. Found: C, 80.87; H, 12.29. MALDI TOF: m/z 497.512 ([M+Na]$^+$, calcd 497.433).

(13Z,17Z,21Z)-Triaconta-13,17,21-trienoic acid (31)

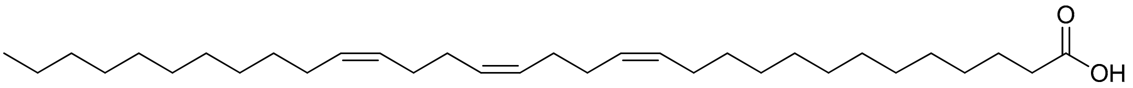

Colorless oil; ^{1}H NMR (400 MHz, $CDCl_3$): 5.46–5.35 (m, 6H), 2.37 (t, J = 7.2 Hz, 2H), 2.13–2.02 (m, 12H), 1.66 (m, 2H), 1.40–1.26 (m, 32H), 0.91 (t, J = 6.4 Hz, 3H); ^{13}C NMR (100 MHz, $CDCl_3$): 180.2, 130.4 (signals of 2C), 129.6 (signals of 2C), 129.1 (signals of 2C), 34.1, 31.9, 29.8–29.1 (signals of 14C), 27.5 (signals of 2C), 27.4 (signals of 2C), 27.3 (signals of 2C), 24.7, 22.7, 14.1; IR (film): 3006, 2924, 2854, 1713, 1463, 1377, 1285, 1242, 1063, 965, 941, 721 cm^{-1}; Analysis calculated for $C_{32}H_{58}O_2$: C, 80.95; H, 12.31. Found: C, 80.88; H, 12.29. MALDI TOF: m/z 497.504 ([M+Na]$^+$, calcd 497.433), 513.489 ([M+K]$^+$, calcd 513.407).

(5*S*)-4-Hydroxy-5-methyl-3-[(9*Z*,13*Z*,17*Z*)-triaconta-9,13,17-trien-1-yl]furan-2(5*H*)-one (33)

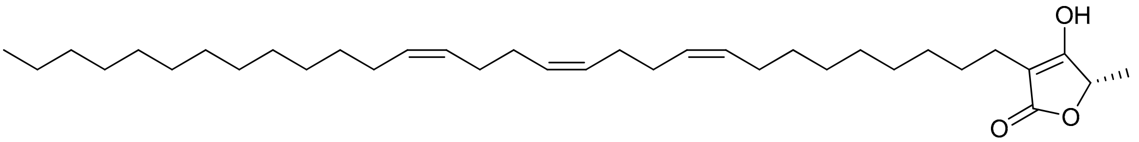

Colourless solid; $[\alpha]_D^{19}$ = – 3.8 (c 0.74, $CHCl_3$); ^{1}H NMR (400 MHz, $CDCl_3$): 5.43–5.36 (m, 6H), 4.85 (q, J = 6.8 Hz, 1H), 2.22 (t, J = 7.2 Hz, 2H), 2.12–2.00 (m, 12H), 1.52 (d, J = 6.8 Hz, 3H), 1.49 (m, 2H), 1.38–1.25 (m, 30H), 0.90 (t, J = 6.8 Hz, 3H); ^{13}C NMR (100 MHz, $CDCl_3$): 177.8, 177.6, 130.4, 130.3, 129.6 (signals of 2C), 129.1 (signals of 2C), 100.9, 75.4, 31.9, 29.7–29.4 (signals of 13C), 28.1, 27.4 (signals of 2C), 27.3 (signals of 4C), 22.7, 21.1, 17.8, 14.1; IR (film): 3005, 2924, 2854, 1722, 1652, 1543, 1456, 1399, 1375, 1342, 1283, 1076, 968, 722 cm^{-1}; Analysis calculated for $C_{35}H_{60}O_3$: C, 79.49; H, 11.44. Found: C, 79.39; H, 11.41. MALDI TOF: m/z 551.524 ([M+Na]$^+$, calcd 551.444), 567.502 ([M+K]$^+$, calcd 567.418).

(5*S*)-4-Hydroxy-5-methyl-3-[(11*Z*,15*Z*,19*Z*)-dotriaconta-11,15,19-trien-1-yl]furan-2(5*H*)-one (34)

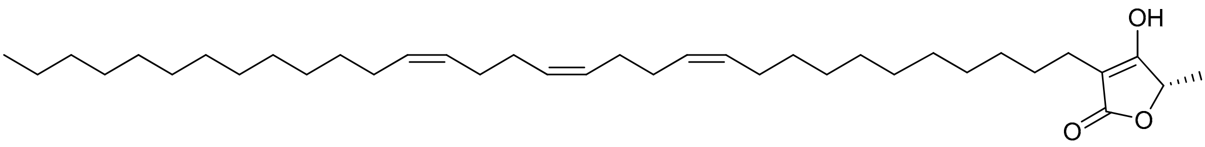

Colourless solid; $[\alpha]_D^{22}$ = – 5.2 (*c* 0.63, $CHCl_3$); ^1H NMR (400 MHz, $CDCl_3$): 5.43–5.36 (m, 6H), 4.85 (q, *J* = 6.8 Hz, 1H), 2.22 (t, *J* = 7.2 Hz, 2H), 2.13–2.00 (m, 12H), 1.52 (d, *J* = 6.8 Hz, 3H), 1.49 (m, 2H), 1.38–1.24 (m, 34H), 0.90 (t, *J* = 6.4 Hz, 3H); ^{13}C NMR (100 MHz, $CDCl_3$): 177.8, 177.6, 130.4, 130.3, 129.6 (signals of 2C), 129.1 (signals of 2C), 100.9, 75.4, 31.9, 29.7–29.3 (signals of 15C), 28.1, 27.5 (signals of 2C), 27.4 (signals of 2C), 27.3 (signals of 2C), 22.7, 21.1, 17.8, 14.1; IR (film): 3005, 2924, 2853, 1732, 1653, 1558, 1542, 1457, 1399, 1076, 968, 722 cm^{-1}; Analysis calculated for $C_{37}H_{64}O_3$: C, 79.80; H, 11.58. Found: C, 79.69; H, 11.55. MALDI TOF: m/z 579.501 ($[M+Na]^+$, calcd 579.475).

(5*S*)-4-Hydroxy-5-methyl-3-[(13*Z*,17*Z*,21*Z*)-dotriaconta-13,17,21-trien-1-yl]furan-2(5*H*)-one (35)

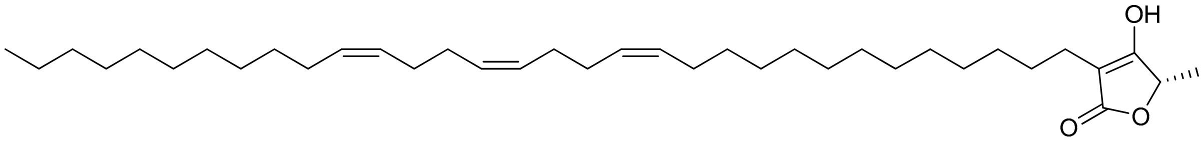

Colourless solid; $[\alpha]_D^{21}$ = – 1.5 (*c* 0.85, $CHCl_3$); ^1H NMR (400 MHz, $CDCl_3$): 5.43–5.35 (m, 6H), 4.82 (q, *J* = 6.4 Hz, 1H), 2.21 (t, *J* = 7.6 Hz, 2H), 2.13–2.00 (m, 12H), 1.50 (d, *J* = 6.8 Hz, 3H), 1.49 (m, 2H), 1.38–1.24 (m, 34H), 0.89 (t, *J* = 6.4 Hz, 3H); ^{13}C NMR (100 MHz, $CDCl_3$): 177.7, 177.5, 130.4 (signals of 2C), 129.6 (signals of 2C), 129.1 (signals of 2C), 101.0, 75.0, 31.9, 29.7–29.4 (signals of 15C), 28.1, 27.5 (signals of 2C), 27.4 (signals of 2C), 27.3 (signals of 2C), 22.7, 21.1, 17.9, 14.1; IR (film): 3005, 2924, 2854, 1752, 1649, 1459, 1400, 1377, 1076, 968, 722 cm^{-1}; Analysis calculated for $C_{37}H_{64}O_3$: C, 79.80; H, 11.58. Found: C, 79.67; H, 11.56. MALDI TOF: m/z 579.426 ($[M+Na]^+$, calcd 579.475), 579.376 ($[M+Na]^+$, calcd 595.449).

(2S)-2-Methyl-5-oxo-4-[(9*Z*,13*Z*,17*Z*)-triaconta-9,13,17-trien-1-yl]-2,5-dihydrofuran-3-yl trifluoromethanesulfonate (37)

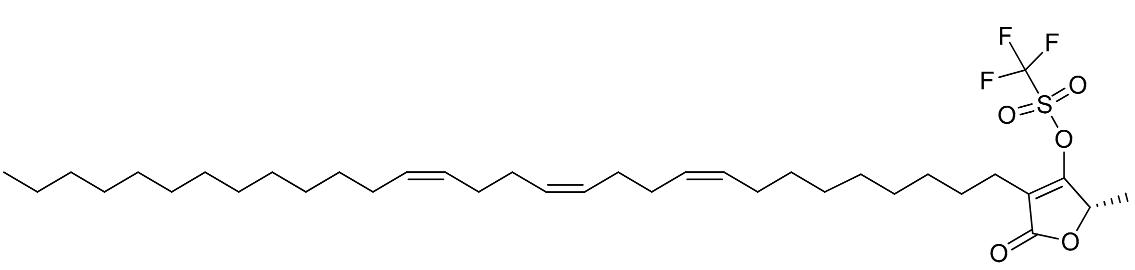

Pale yellow oil; $[\alpha]_D^{19}$ = + 30.9 (*c* 0.73, $CHCl_3$); ^{1}H NMR (500 MHz, $CDCl_3$): 5.44–5.37 (m, 6H), 5.13 (q, *J* = 6.4 Hz, 1H), 2.33 (m, 2H), 2.13–2.00 (m, 12H), 1.60 (m, 2H), 1.56 (d, *J* = 6.4 Hz, 3H), 1.37–1.26 (m, 30H), 0.89 (t, *J* = 6.4 Hz, 3H); ^{13}C NMR (125 MHz, $CDCl_3$): 169.0, 163.4, 130.4, 130.3, 129.6 (signals of 2C), 129.1 (signals of 2C), 121.9, 118.4 (*J* = 319 Hz), 74.4, 31.9, 29.7–29.1 (signals of 13C), 27.5, 27.4 (signals of 3C), 27.3, 27.2, 26.7, 22.7 (signals of 2C), 17.7, 14.1; IR (film): 3006, 2925, 2854, 1782, 1699, 1436, 1379, 1341, 1222, 1138, 1104, 1068, 944, 808, 764, 723, 605 cm^{-1}; Analysis calculated for $C_{36}H_{59}F_3O_5S$: C, 65.42; H, 9.00. Found: C, 65.35; H, 8.98. MALDI TOF: m/z 683.503 ([M+Na]$^+$, calcd 683.404).

(2S)-2-Methyl-5-oxo-4-[(11*Z*,15*Z*,19*Z*)-triaconta-11,15,19-trien-1-yl]-2,5-dihydrofuran-3-yl trifluoromethanesulfonate (38)

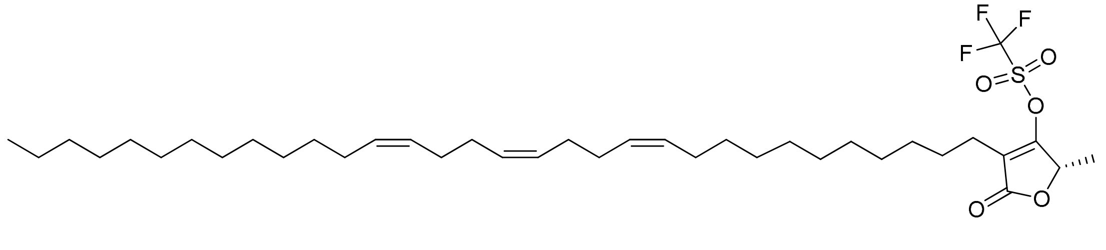

Pale yellow oil; $[\alpha]_D^{22}$ = + 17.4 (*c* 0.84, $CHCl_3$); ^{1}H NMR (400 MHz, $CDCl_3$): 5.44–5.37 (m, 6H), 5.13 (q, *J* = 6.4 Hz, 1H), 2.33 (m, 2H), 2.13–2.00 (m, 12H), 1.60 (m, 2H), 1.56 (d, *J* = 6.4 Hz, 3H), 1.37–1.25 (m, 34H), 0.90 (t, *J* = 6.8 Hz, 3H); ^{13}C NMR (100 MHz, $CDCl_3$): 169.1, 163.4, 130.4 (signals of 2C), 129.6 (signals of 2C), 129.1 (signals of 2C), 121.9, 118.4 (*J* = 319 Hz), 74.4, 31.9, 29.7–29.1 (signals of 15C), 27.5 (signals of 2C), 27.4 (signals of 2C), 27.3 (signals of 2C), 26.7, 22.7 (signals of 2C), 17.7, 14.1; IR (film): 3006, 2925, 2854, 1782, 1699, 1436, 1378, 1340, 1222, 1139, 1103, 1068, 940, 808, 764, 723, 605 cm^{-1}; Analysis calculated for $C_{38}H_{63}F_3O_5S$: C, 66.25; H, 9.22. Found: C, 66.13; H, 9.19. MALDI TOF: m/z 711.513 ([M+Na]$^+$, calcd 711.425), 727.417 ([M+Na]$^+$, calcd 727.399).

(2S)-2-Methyl-5-oxo-4-[(13*Z*,17*Z*,21*Z*)-triaconta-13,17,21-trien-1-yl]-2,5-dihydrofuran-3-yl trifluoromethanesulfonate (39)

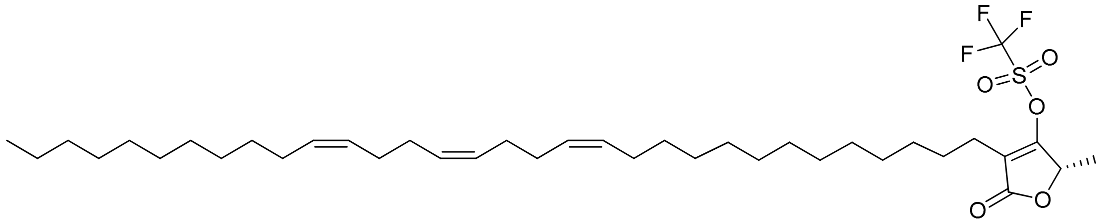

Pale yellow oil; $[\alpha]_D^{19}$ = + 22. (*c* 0.85, $CHCl_3$); ^{1}H NMR (500 MHz, $CDCl_3$): 5.45–5.35 (m, 6H), 5.13 (q, *J* = 6.4 Hz, 1H), 2.33 (m, 2H), 2.13–2.01 (m, 12H), 1.61 (m, 2H), 1.56 (d, *J* = 6.8 Hz, 3H), 1.38–1.25 (m, 34H), 0.89 (t, *J* = 6.8 Hz, 3H); ^{13}C NMR (125 MHz, $CDCl_3$): 169.0, 163.4, 130.4 (signals of 2C), 129.6 (signals of 2C), 129.1 (signals of 2C), 121.9, 118.4 (*J* = 319 Hz), 74.4, 31.9, 29.8–29.1 (signals of 15C), 27.5 (signals of 2C), 27.4 (signals of 2C), 27.3 (signals of 2C), 26.7, 22.7 (signals of 2C), 17.7, 14.1; IR (film): 3006, 2926, 2854, 1782, 1699, 1436, 1341, 1323, 1223, 1138, 1102, 1068, 939, 808, 764, 723, 605 cm^{-1}; Analysis calculated for $C_{38}H_{63}F_3O_5S$: C, 66.25; H, 9.22. Found: C, 66.11; H, 9.20. MALDI TOF: m/z 711.282 ($[M+Na]^+$, calcd 711.425).

(5S)-5-Methyl-3-[(9*Z*,13*Z*,17*Z*)-triaconta-9,13,17-trien-1-yl]furan-2(5*H*)-one (2) (Chatenaytrienin-2)

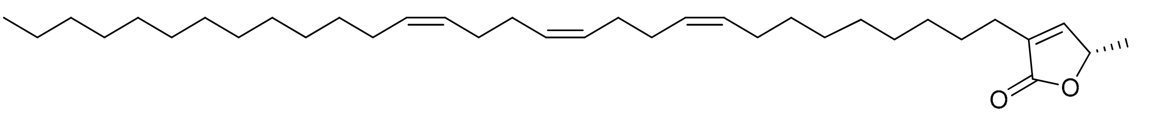

Colourless waxy solid; $[\alpha]_D^{21}$ = + 12.2 (*c* 0.76, $CHCl_3$); ^{1}H NMR (500 MHz, $CDCl_3$): 6.99 (d, ^3J = 1.2 Hz, 1H), 5.45–5.34 (m, 6H), 5.00 (qd, ^3J = 6.8, ^3J = 1.6 Hz, 1H), 2.28 (t, *J* = 7.6 Hz, 2H), 2.13–2.00 (m, 12H), 1.57 (m, 2H), 1.42 (d, *J* = 6.8 Hz, 3H), 1.38–1.26 (m, 30H), 0.89 (t, *J* = 6.8 Hz, 3H); ^{13}C NMR (125 MHz, $CDCl_3$): 173.9, 148.8, 134.3, 130.4, 130.3, 129.6 (signals of 2C), 129.2, 129.1, 77.3, 31.9, 29.8–29.2 (signals of 13C), 27.5, 27.4 (signals of 4C), 27.3 (signals of 2C), 25.2, 22.7, 19.2, 14.1; IR (film): 3005, 2925, 2853, 1759, 1616, 1456, 1374, 1318, 1198, 1072, 1027, 843, 742, 699 cm^{-1}; Analysis calculated for $C_{35}H_{60}O_2$: C, 81.97; H, 11.79. Found: C, 81.83; H, 11.75. MALDI TOF: m/z 535.481 ($[M+Na]^+$, calcd 535.449), 551.529 ($[M+K]^+$, calcd 551.423). HRMS (ESI-TOF): calcd for $C_{35}H_{60}O_2Na$ $[M+Na]^+$ 535.4491, found 535.4437.

(5S)-5-Methyl-3-[(11*Z*,15*Z*,19*Z*)-triaconta-11,15,19-trien-1-yl]furan-2(5*H*)-one (3) (Chatenaytrienin-3)

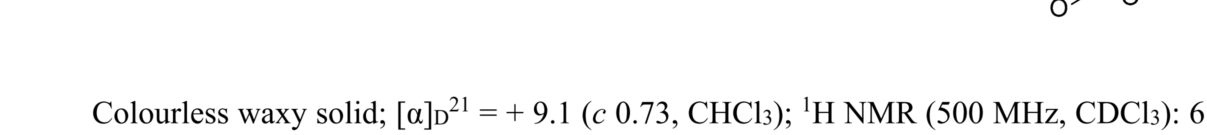

Colourless waxy solid; $[\alpha]_D^{21}$ = + 9.1 (*c* 0.73, $CHCl_3$); ^{1}H NMR (500 MHz, $CDCl_3$): 6.99 (d, ^3J = 1.2 Hz, 1H), 5.45–5.34 (m, 6H), 5.00 (qd, ^3J = 6.8, ^3J = 1.6 Hz, 1H), 2.28 (t, *J* = 7.8 Hz, 2H), 2.13–2.00 (m, 12H), 1.56 (m, 2H), 1.42 (d, *J* = 6.4 Hz, 3H), 1.38–1.26 (m, 34H), 0.90 (t, *J* =

6.8 Hz, 3H); ^{13}C NMR (125 MHz, $CDCl_3$): 173.9, 148.8, 134.3, 130.4 (signals of 2C), 129.6 (signals of 2C), 129.1 (signals of 2C), 77.3, 31.9, 29.8–29.2 (signals of 15C), 27.5 (signals of 2C), 27.4 (signals of 2C), 27.3 (signals of 3C), 25.2, 22.7, 19.2, 14.1; IR (film): 3006, 2924, 2853, 1760, 1618, 1464, 1374, 1318, 1180, 1118, 1077, 1027, 857, 798, 722 cm^{-1}; Analysis calculated for $C_{37}H_{64}O_2$: C, 82.16; H, 11.93. Found: C, 82.01; H, 11.93. MALDI TOF: m/z 563.453 ([M+Na]$^+$, calcd 563.480), 579.509 ([M+K]$^+$, calcd 579.454). HRMS (ESI-TOF): calcd for $C_{37}H_{64}O_2Na$ [M+Na]$^+$ 563.4804, found 563.4626.

(5S)-5-Methyl-3-[(13*Z*,17*Z*,21*Z*)-triaconta-13,17,21-trien-1-yl]furan-2(5*H*)-one (4) (Chatenaytrienin-4)

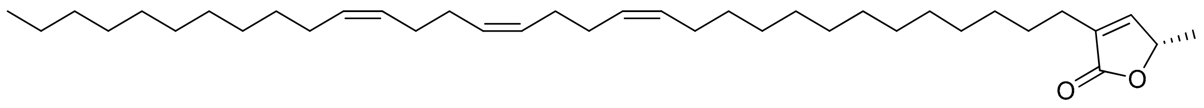

Colourless waxy solid; $[\alpha]_D^{17}$ = + 29.0 (*c* 0.74, $CHCl_3$); ^{1}H NMR (500 MHz, $CDCl_3$): 7.00 (d, ^3J = 1.2 Hz, 1H), 5.45–5.34 (m, 6H), 5.01 (qd, ^3J = 6.8, ^3J = 1.6 Hz, 1H), 2.28 (t, J = 7.6 Hz, 2H), 2.13–1.99 (m, 12H), 1.55 (m, 2H), 1.42 (d, J = 6.4 Hz, 3H), 1.38–1.26 (m, 34H), 0.90 (t, J = 6.5 Hz, 3H); ^{13}C NMR (125 MHz, $CDCl_3$): 173.9, 148.8, 134.4, 130.4 (signals of 2C), 129.6 (signals of 2C), 129.1 (signals of 2C), 77.4, 31.9, 29.8–29.2 (signals of 15C), 27.5 (signals of 2C), 27.4 (signals of 2C), 27.3 (signals of 3C), 25.2, 22.7, 19.2, 14.1; IR (film): 3006, 2924, 2853, 1759, 1619, 1464, 1376, 1318, 1261, 1195, 1076, 1028, 857, 799, 723 cm^{-1}; Analysis calculated for $C_{37}H_{64}O_2$: C, 82.16; H, 11.93. Found: C, 82.00; H, 11.92. MALDI TOF: m/z 563.418 ([M+Na]$^+$, calcd 563.480), 579.375 ([M+K]$^+$, calcd 579.454). HRMS (ESI-TOF): calcd for $C_{37}H_{64}O_2Na$ [M+Na]$^+$ 563.4804, found 563.4709.

Figures for spectrum of the compounds 25–27, 29–31, 33–35, 37–39

Figure S1. ^{13}C NMR Spectrum of compound **25** (125 MHz, $CDCl_3$)

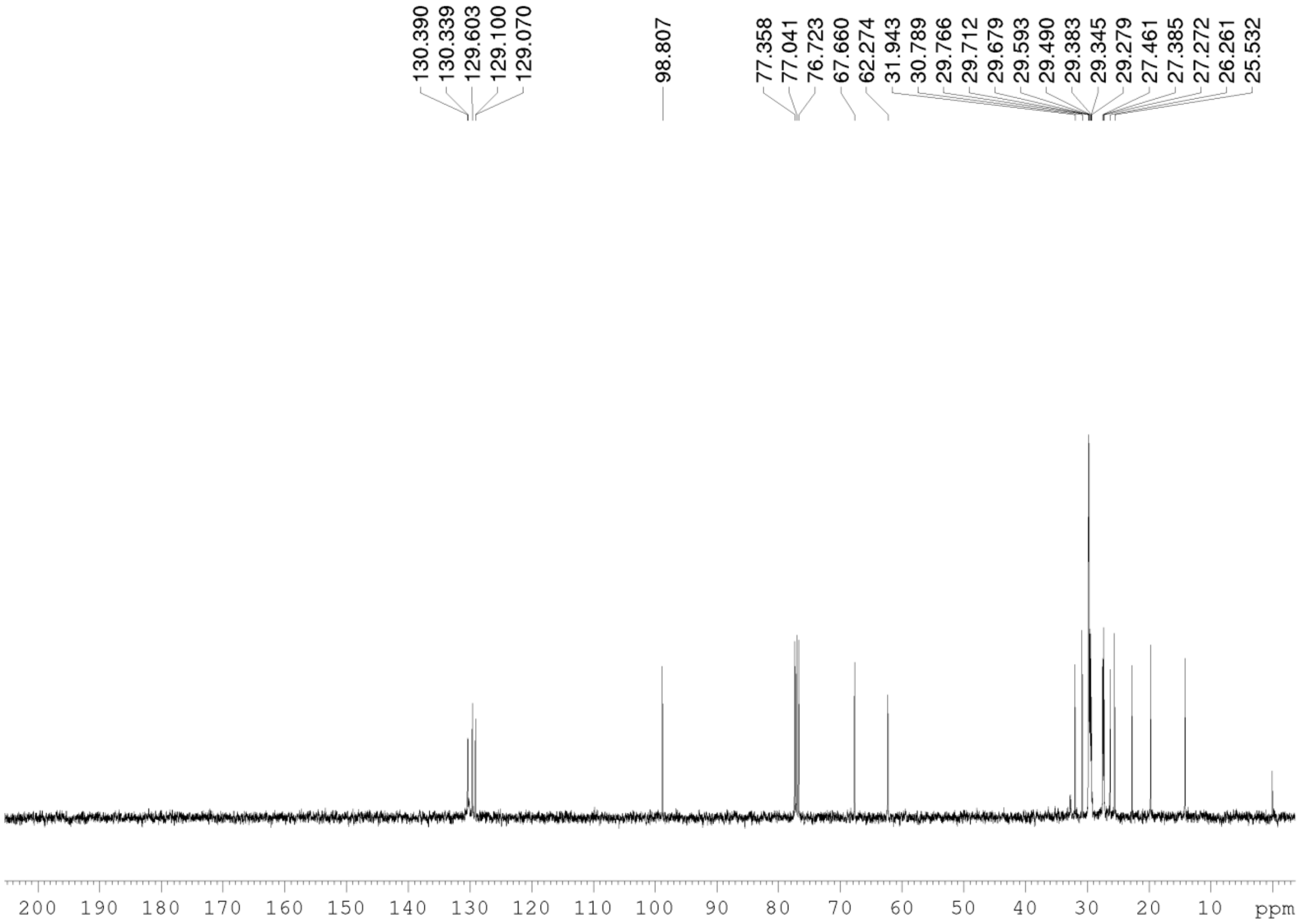

Figure S2. ^{1}H NMR Spectrum of compound **25** (500 MHz, $CDCl_3$)

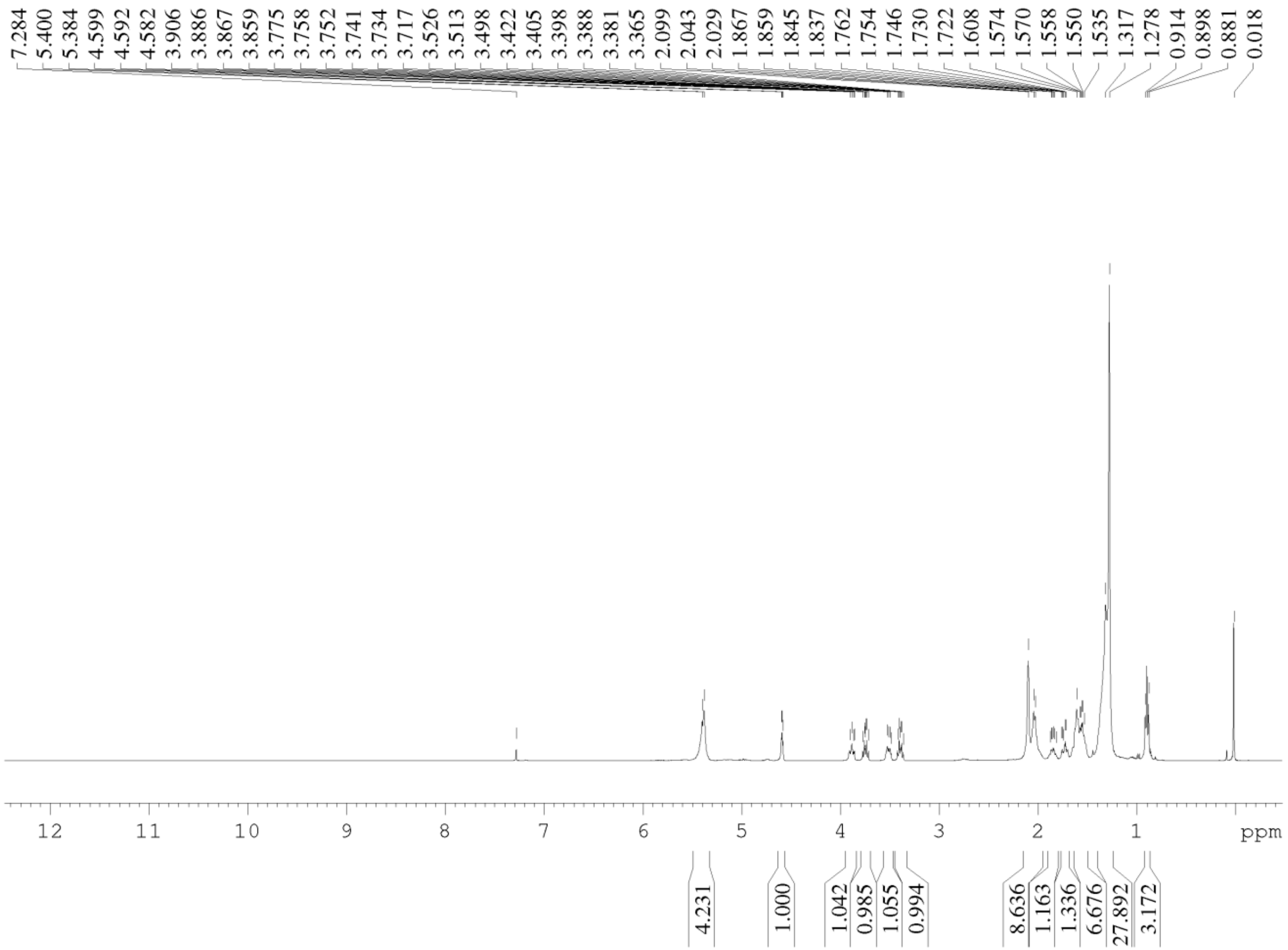

Figure S3. ^{13}C NMR Spectrum of compound **26** (125 MHz, $CDCl_3$)

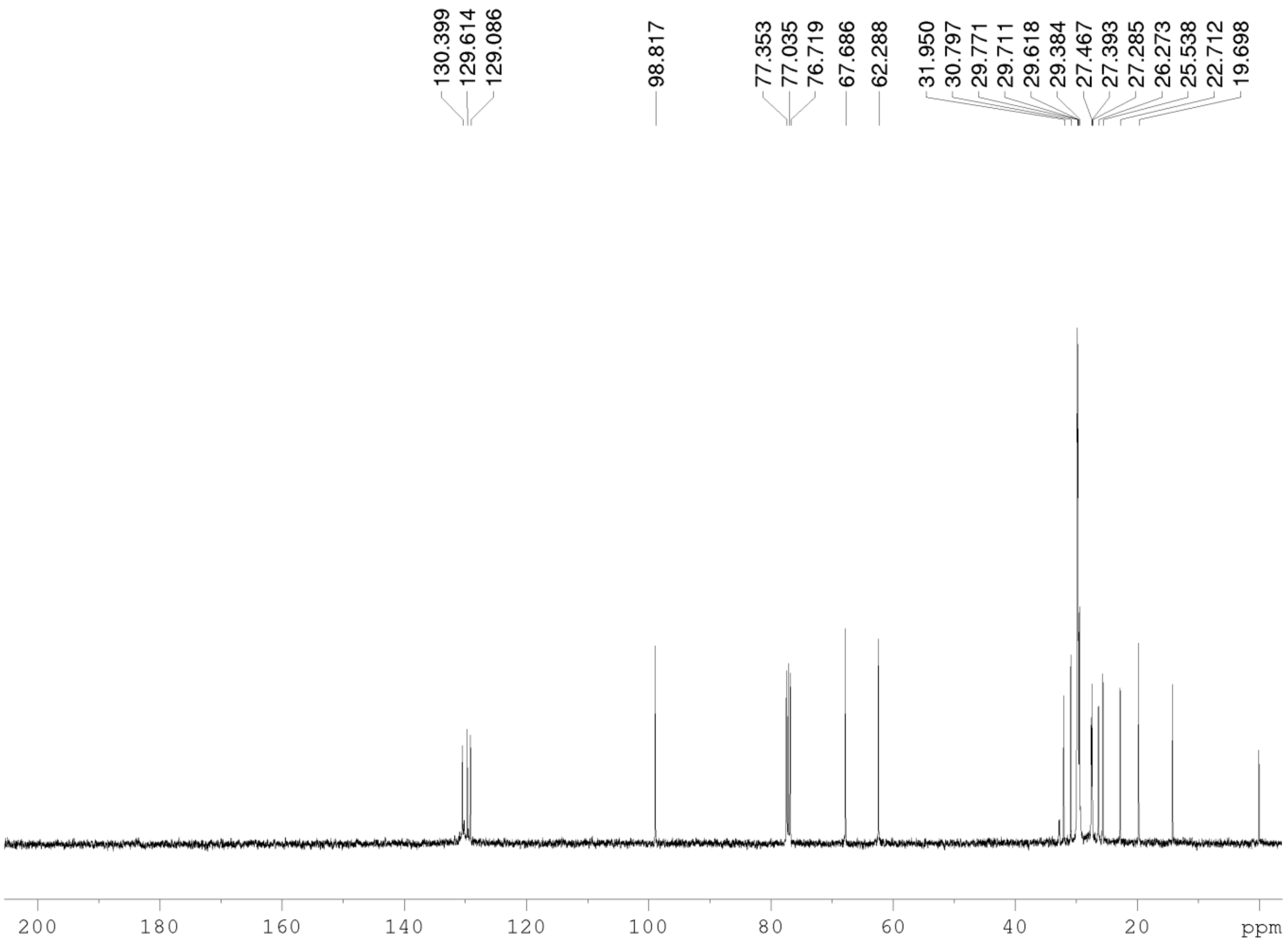

Figure S4. ^{1}H NMR Spectrum of compound **26** (500 MHz, $CDCl_3$)

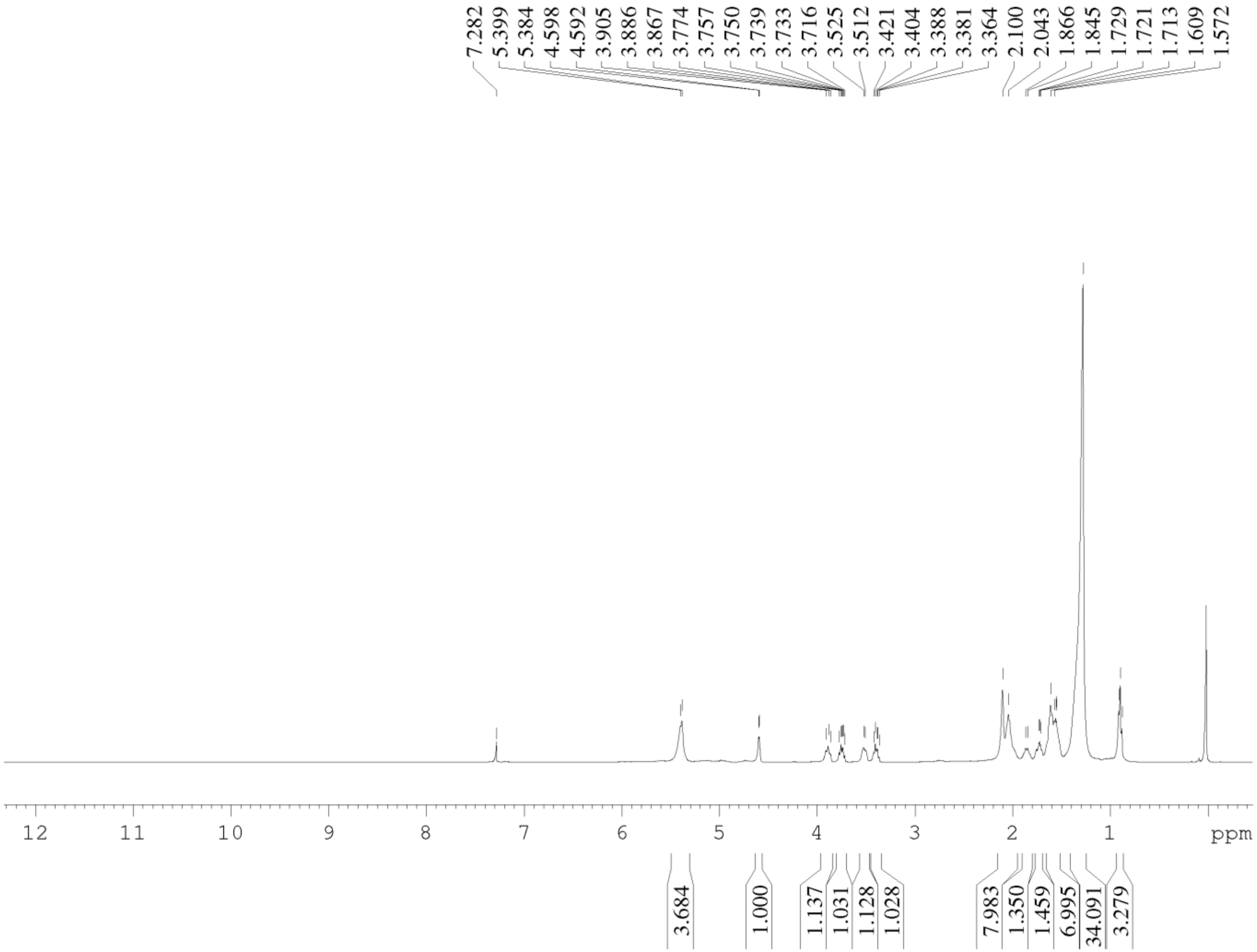

Figure S5. ^{13}C NMR Spectrum of compound **27** (125 MHz, $CDCl_3$)

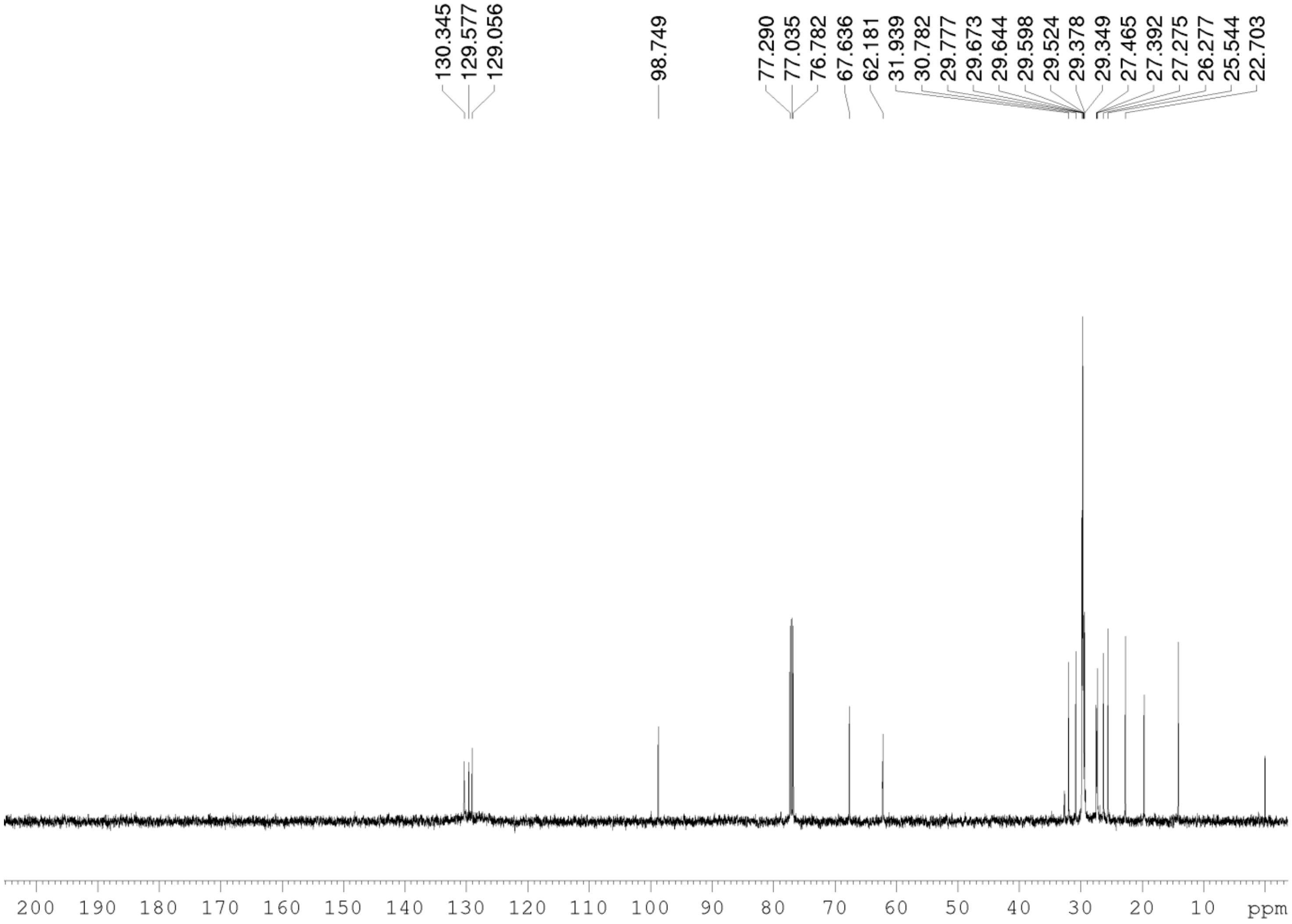

Figure S6. ^{1}H NMR Spectrum of compound **27** (500 MHz, $CDCl_3$)

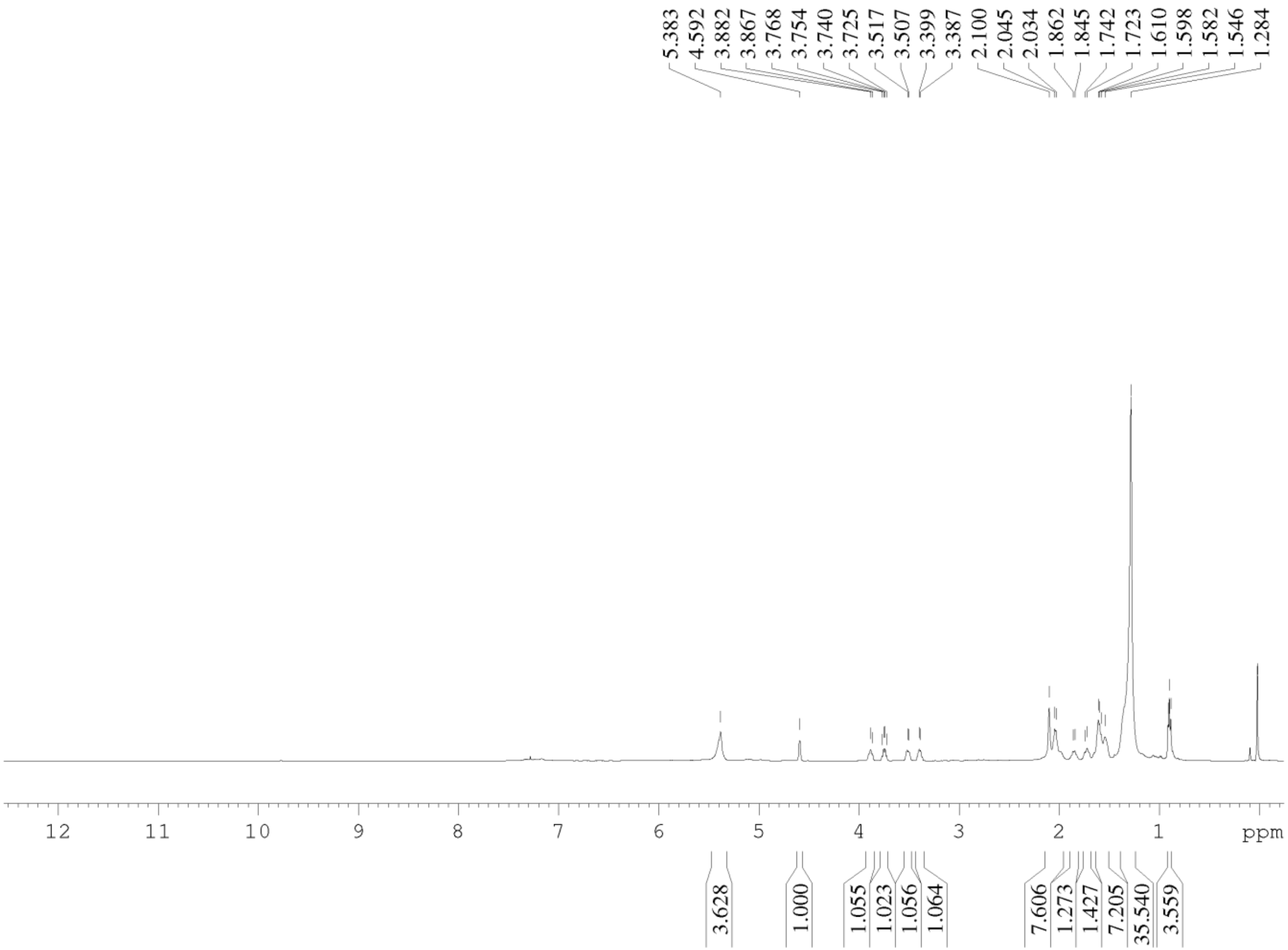

Figure S7. ^{13}C NMR Spectrum of compound **29** (100 MHz, $CDCl_3$)

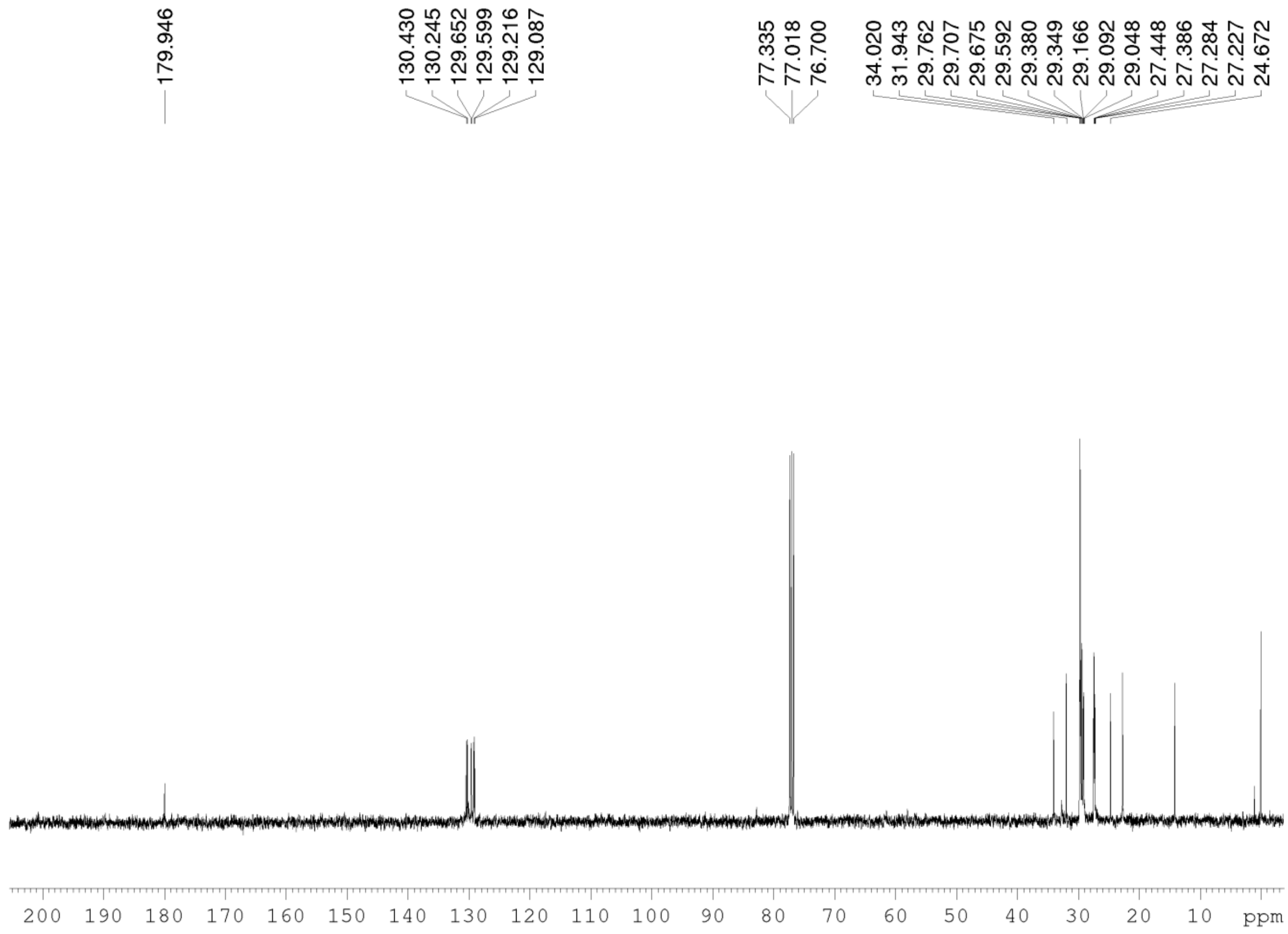

Figure S8. ^{1}H NMR Spectrum of compound **29** (400 MHz, $CDCl_3$)

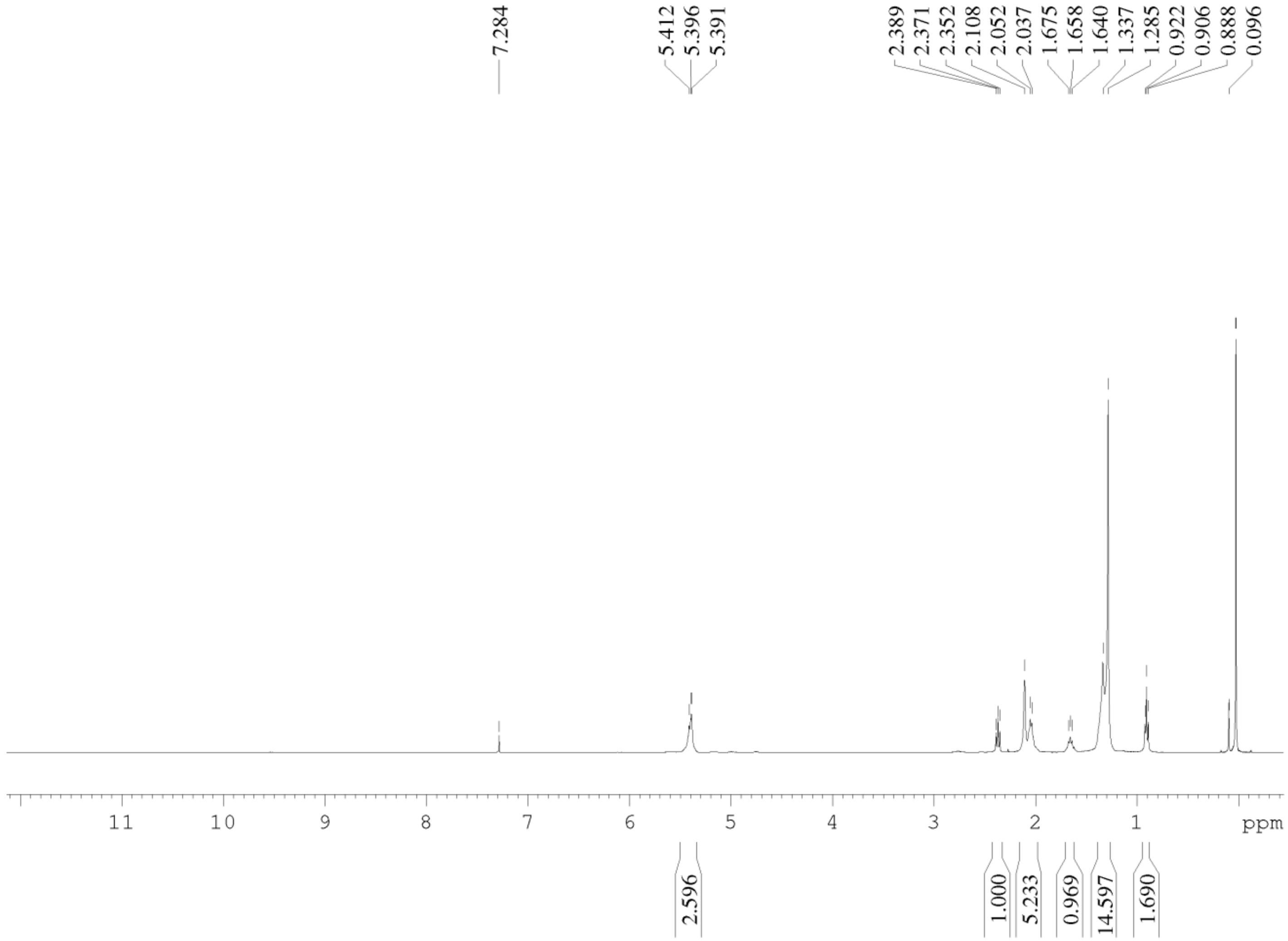

Figure S9. ^{13}C NMR Spectrum of compound **30** (100 MHz, $CDCl_3$)

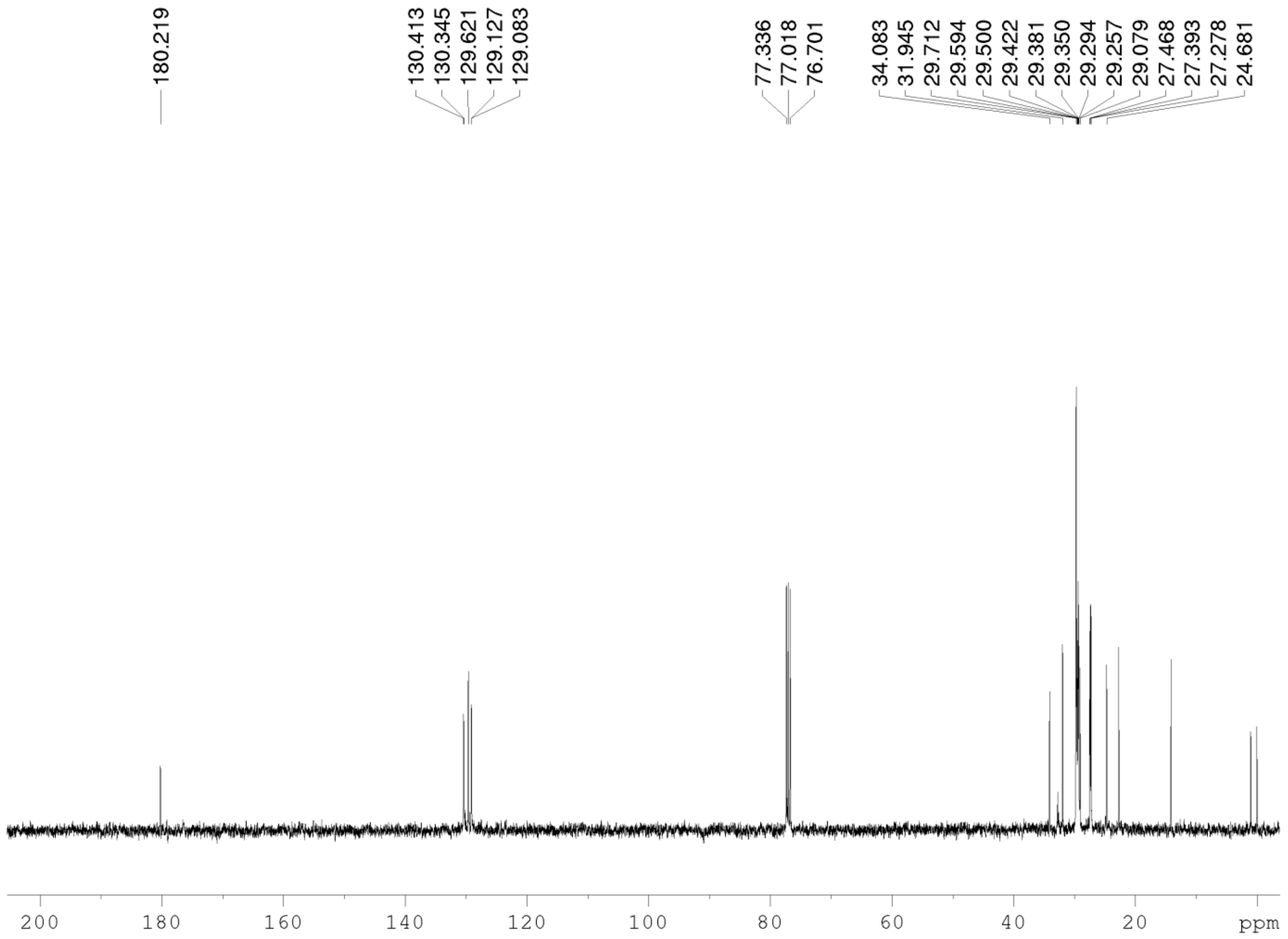

Figure S10. ^{1}H NMR Spectrum of compound **30** (400 MHz, $CDCl_3$)

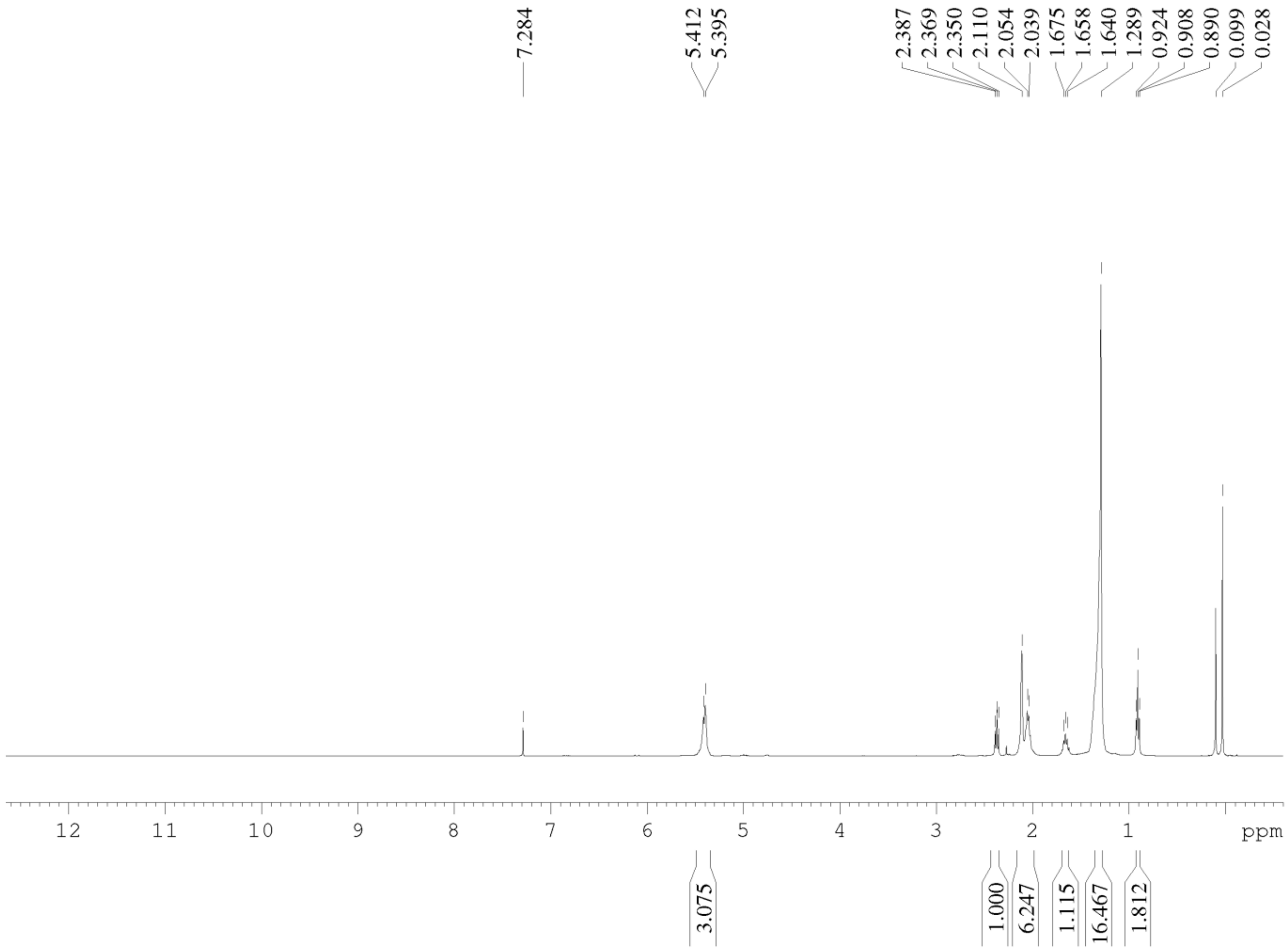

Figure S11. ^{13}C NMR Spectrum of compound **31** (100 MHz, $CDCl_3$)

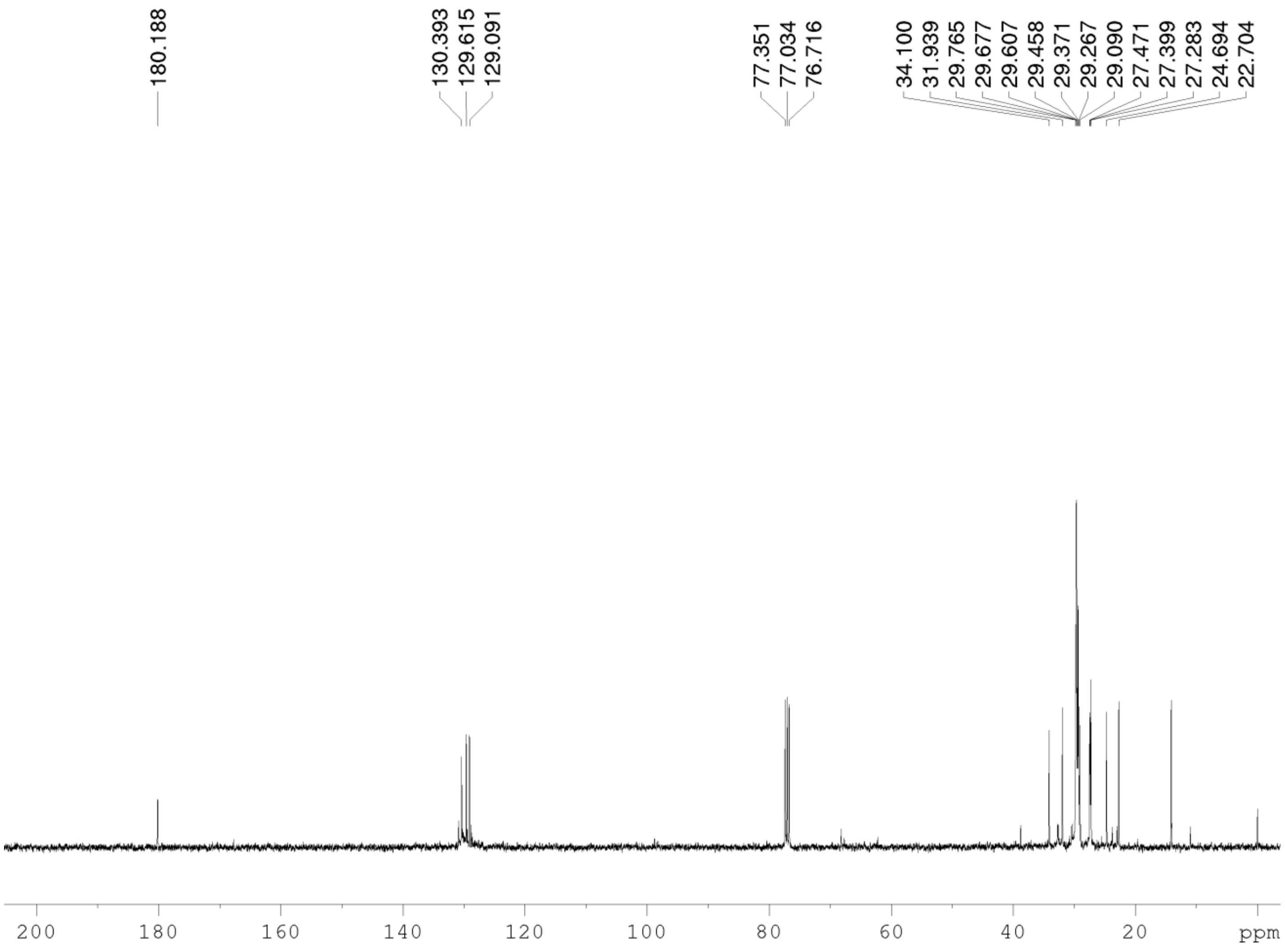

Figure S12. ^{1}H NMR Spectrum of compound **31** (400 MHz, $CDCl_3$)

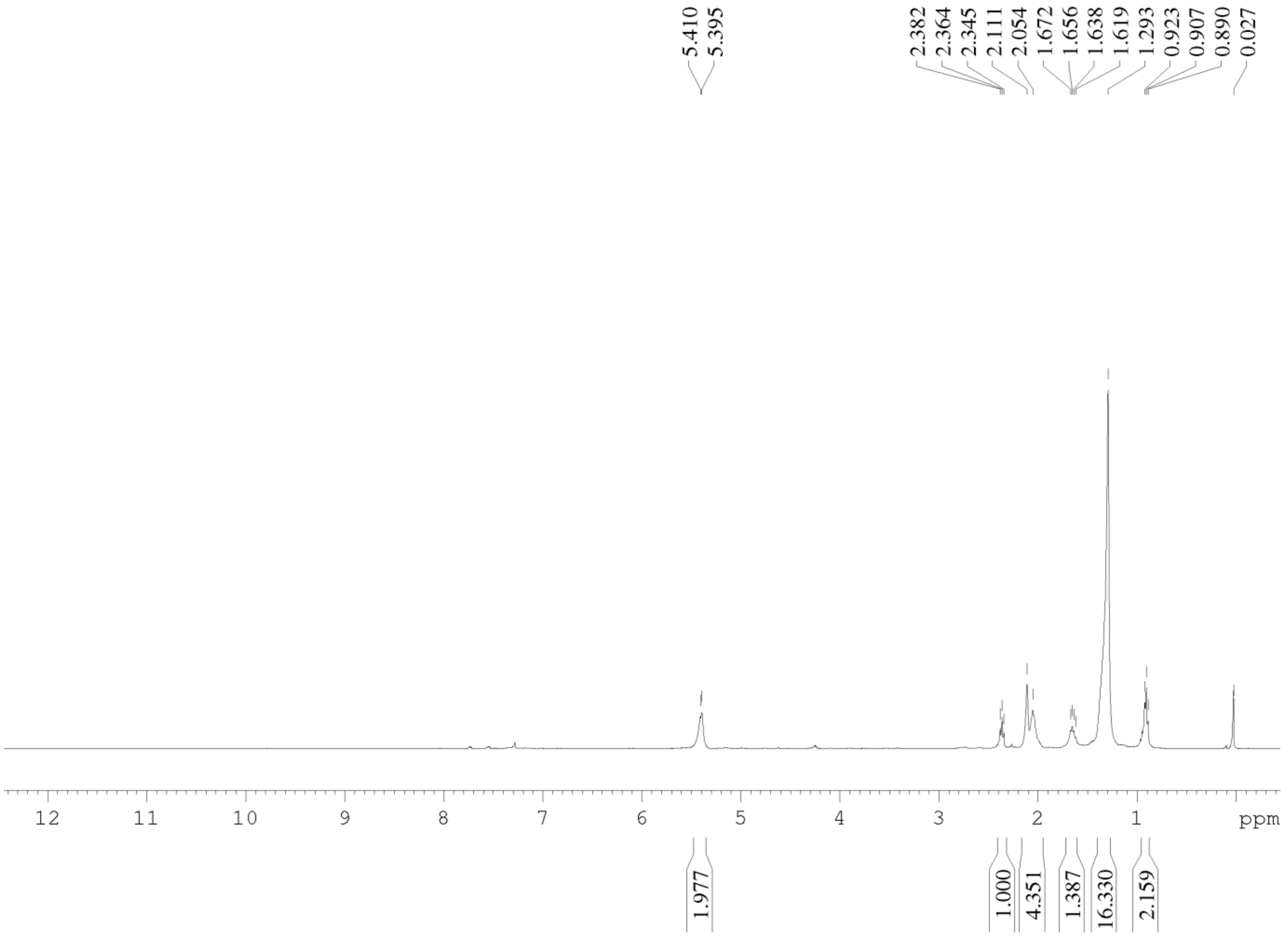

Figure S13. ^{13}C NMR Spectrum of compound **33** (100 MHz, $CDCl_3$)

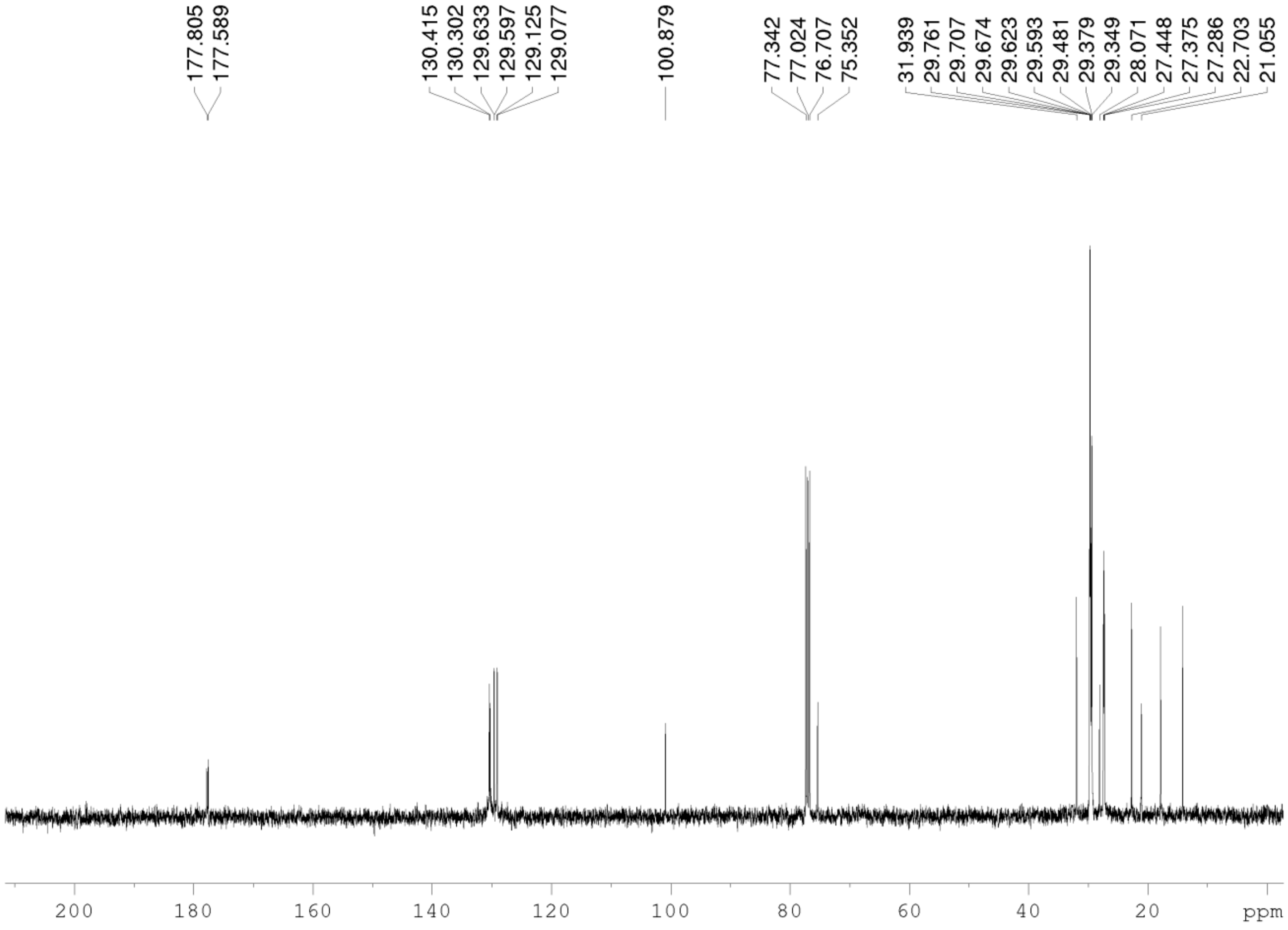

Figure S14. ^{1}H NMR Spectrum of compound **33** (400 MHz, $CDCl_3$)

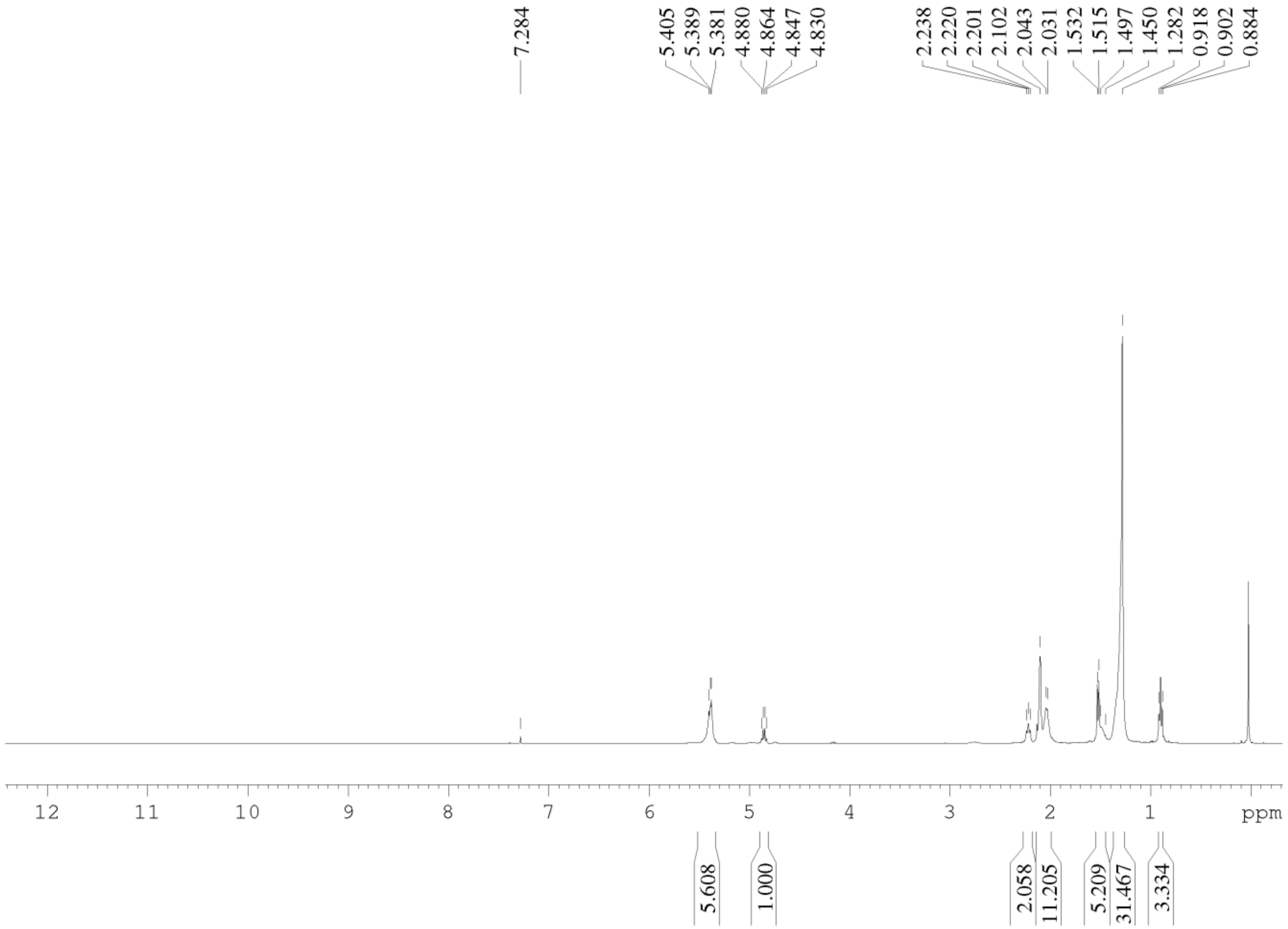

Figure S15. ^{13}C NMR Spectrum of compound **34** (100 MHz, $CDCl_3$)

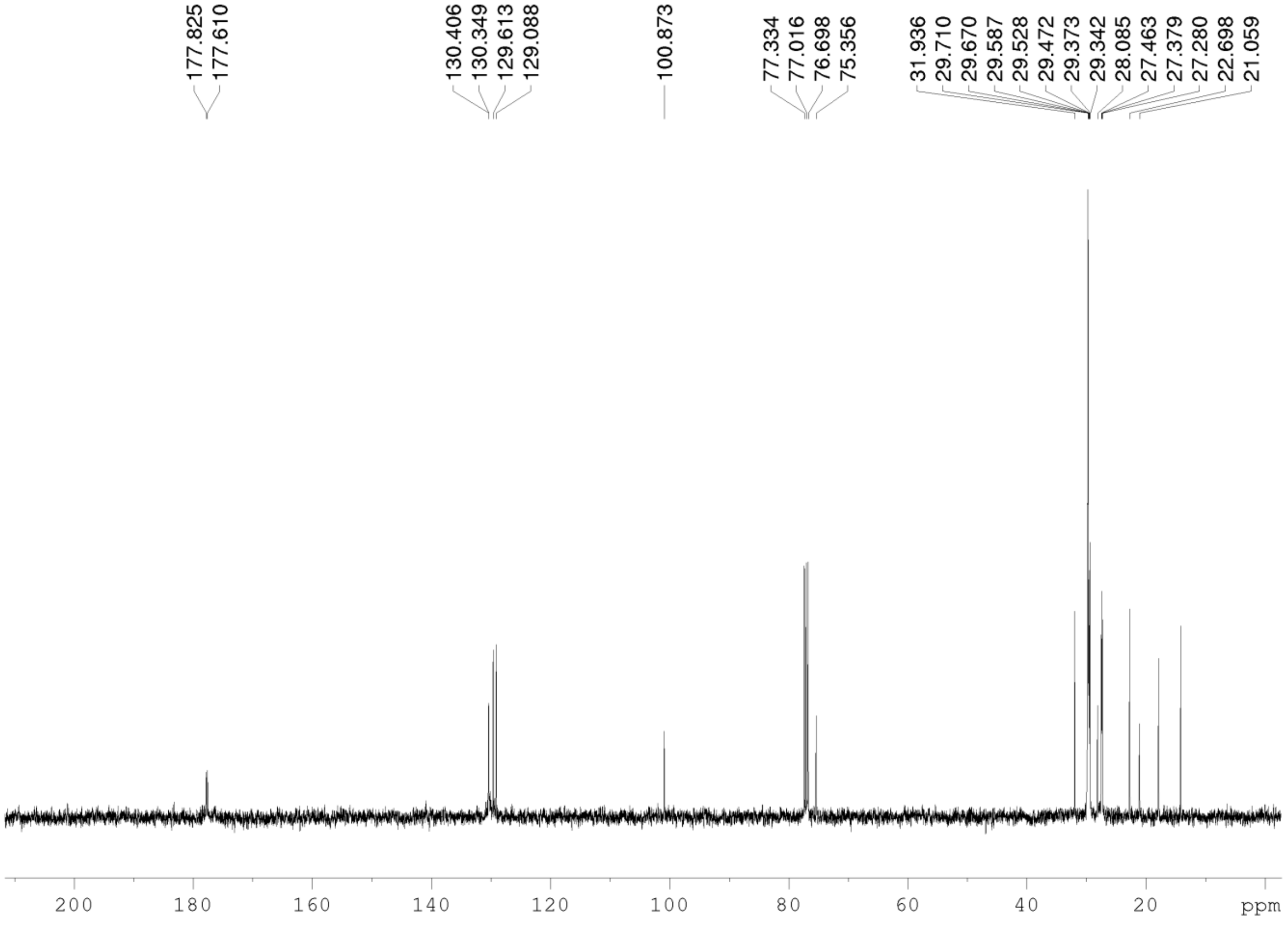

Figure S16. ^{1}H NMR Spectrum of compound **34** (400 MHz, $CDCl_3$)

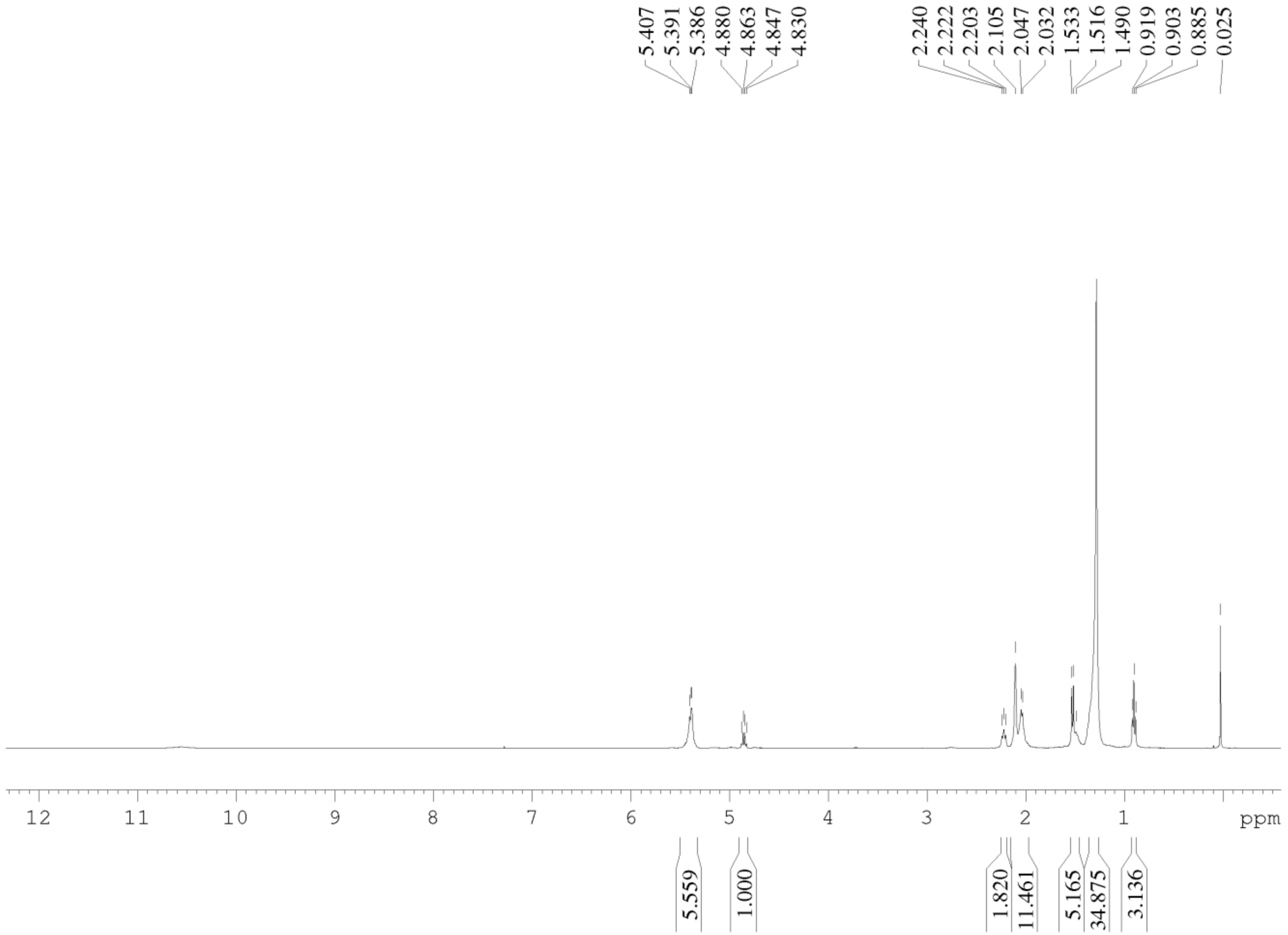

Figure S17. ^{13}C NMR Spectrum of compound **35** (100 MHz, $CDCl_3$)

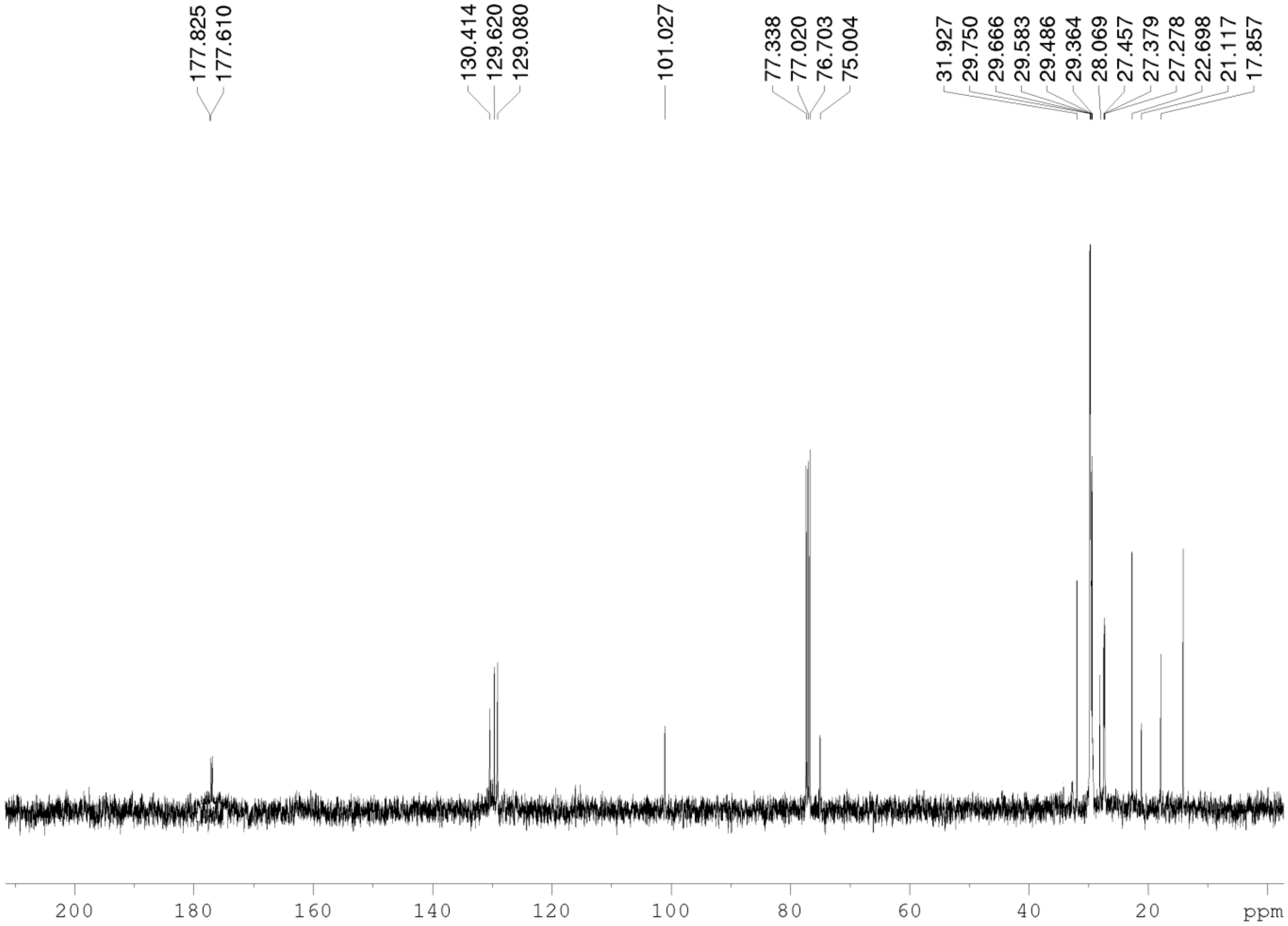

Figure S18. ^{1}H NMR Spectrum of compound **35** (400 MHz, $CDCl_3$)

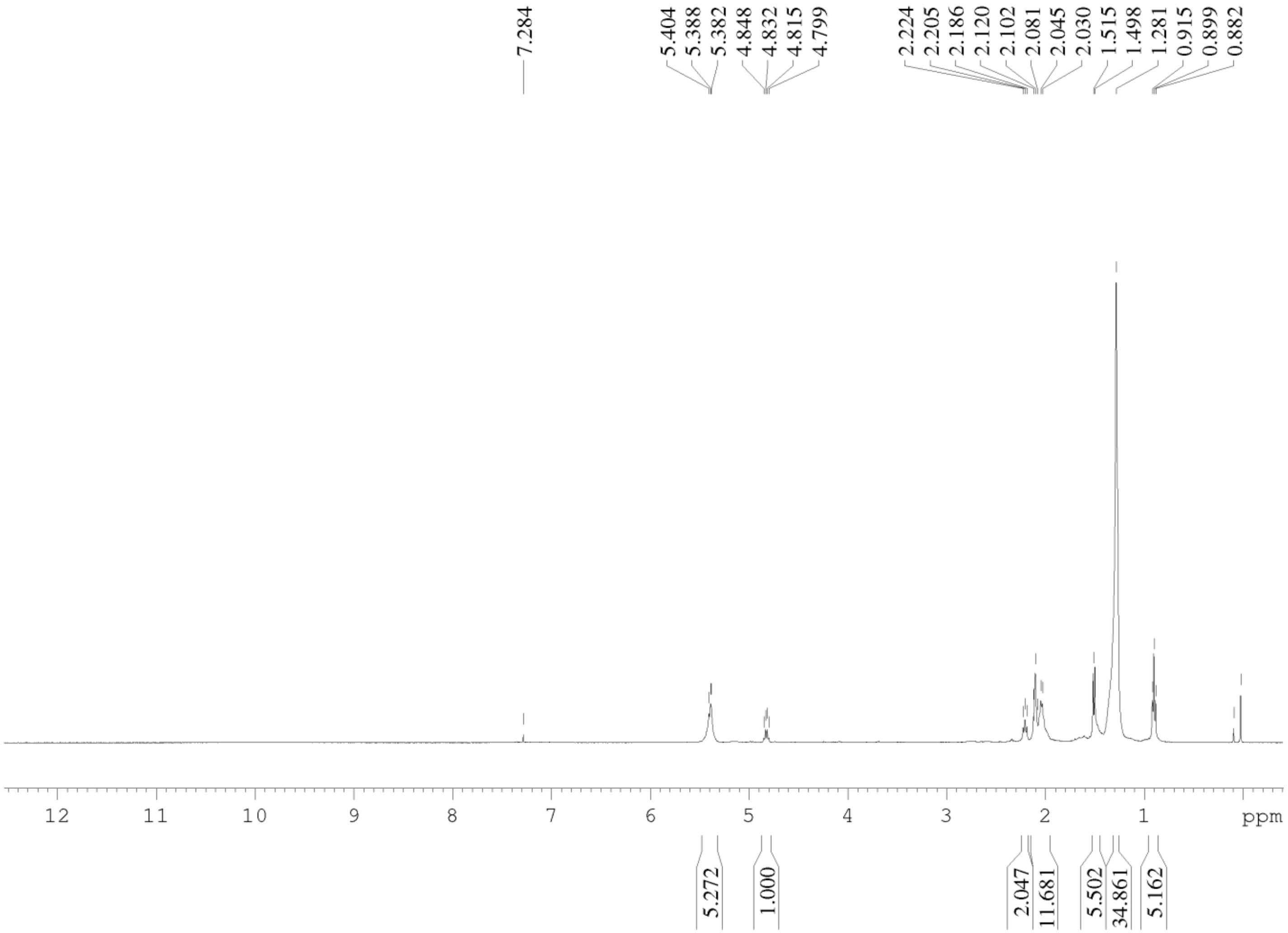

Figure S19. ^{13}C NMR Spectrum of compound **37** (125 MHz, $CDCl_3$)

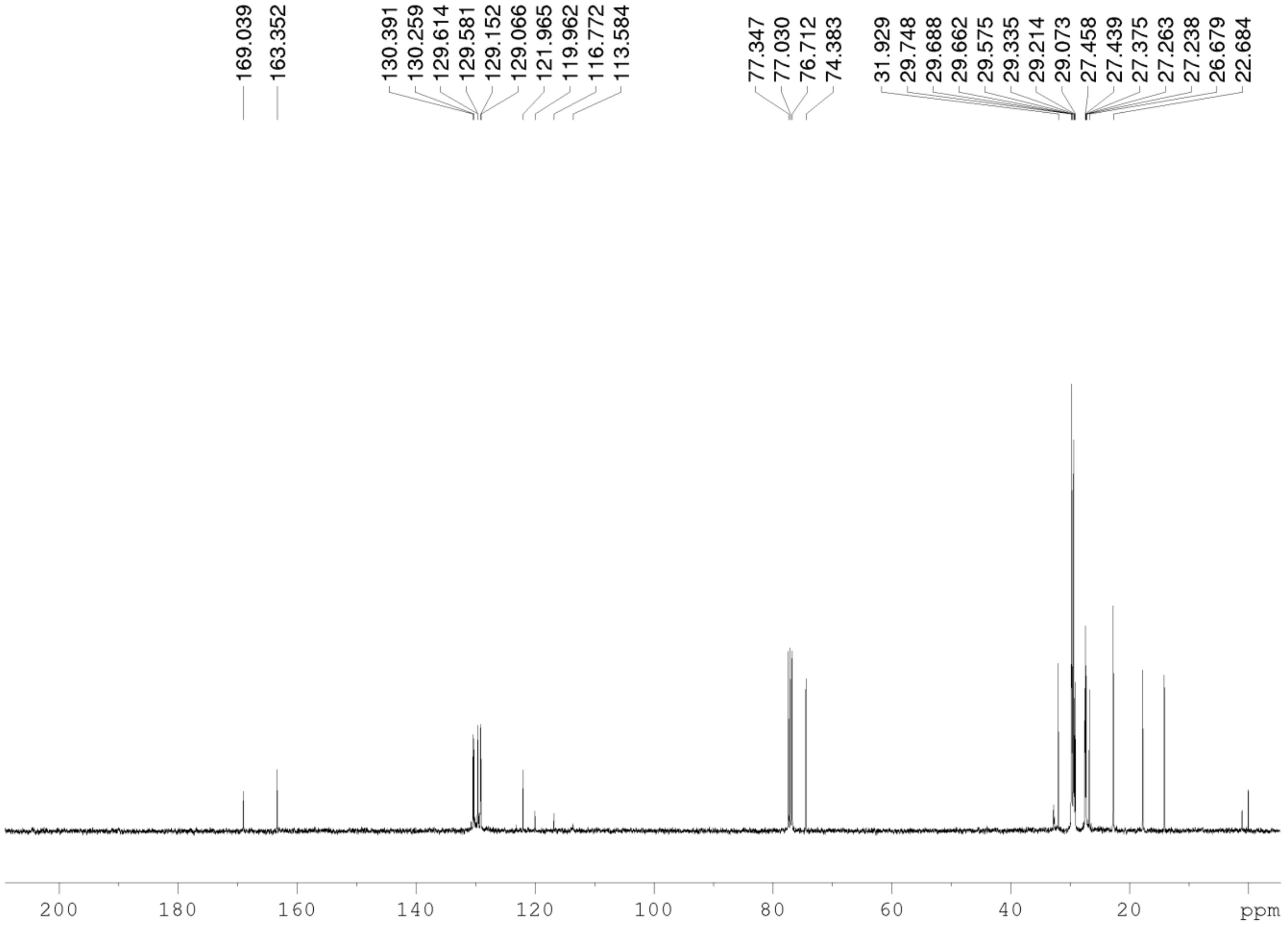

Figure S20. ^{1}H NMR Spectrum of compound **37** (500 MHz, $CDCl_3$)

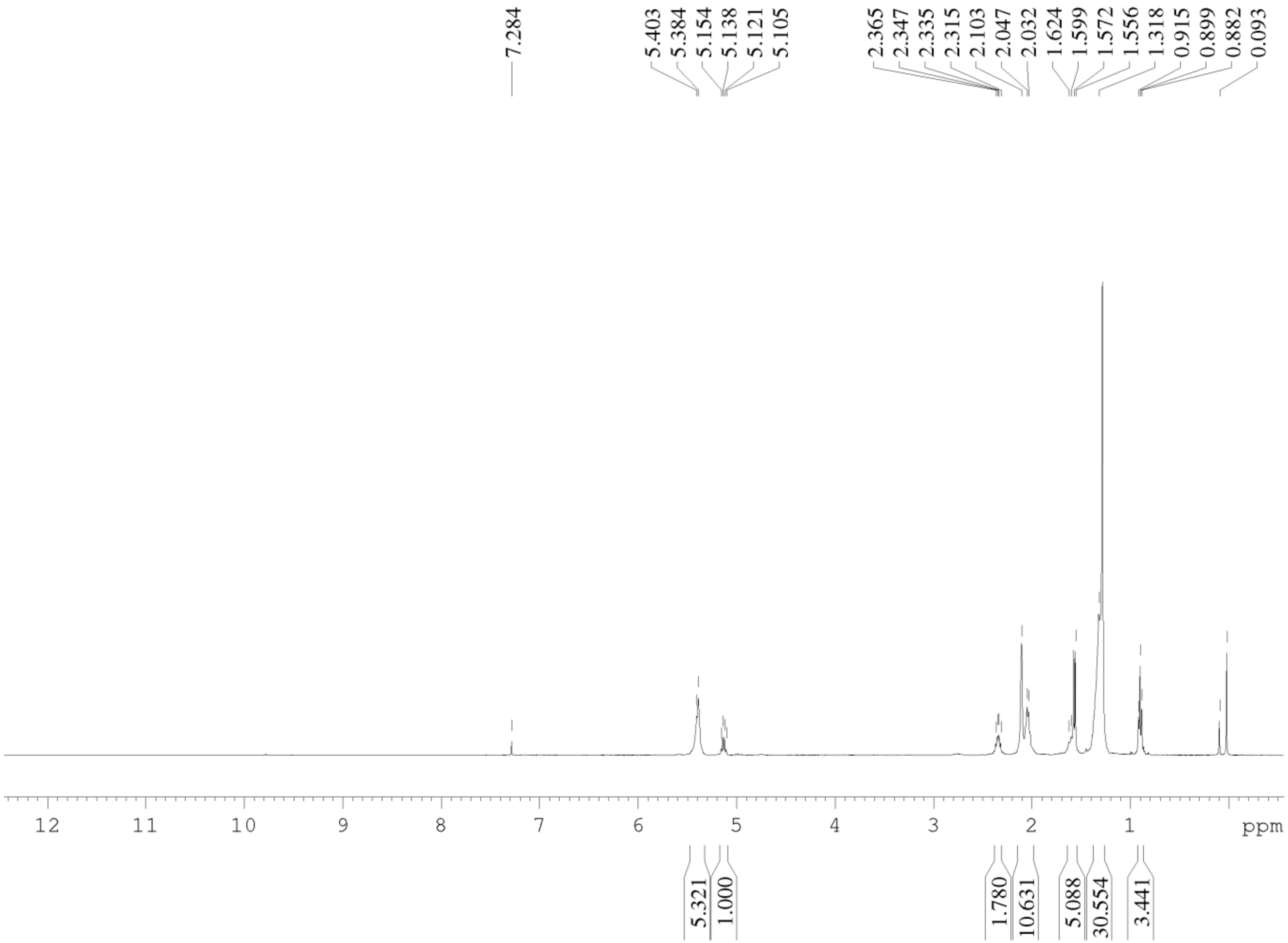

Figure S21. ^{13}C NMR Spectrum of compound **38** (100 MHz, $CDCl_3$)

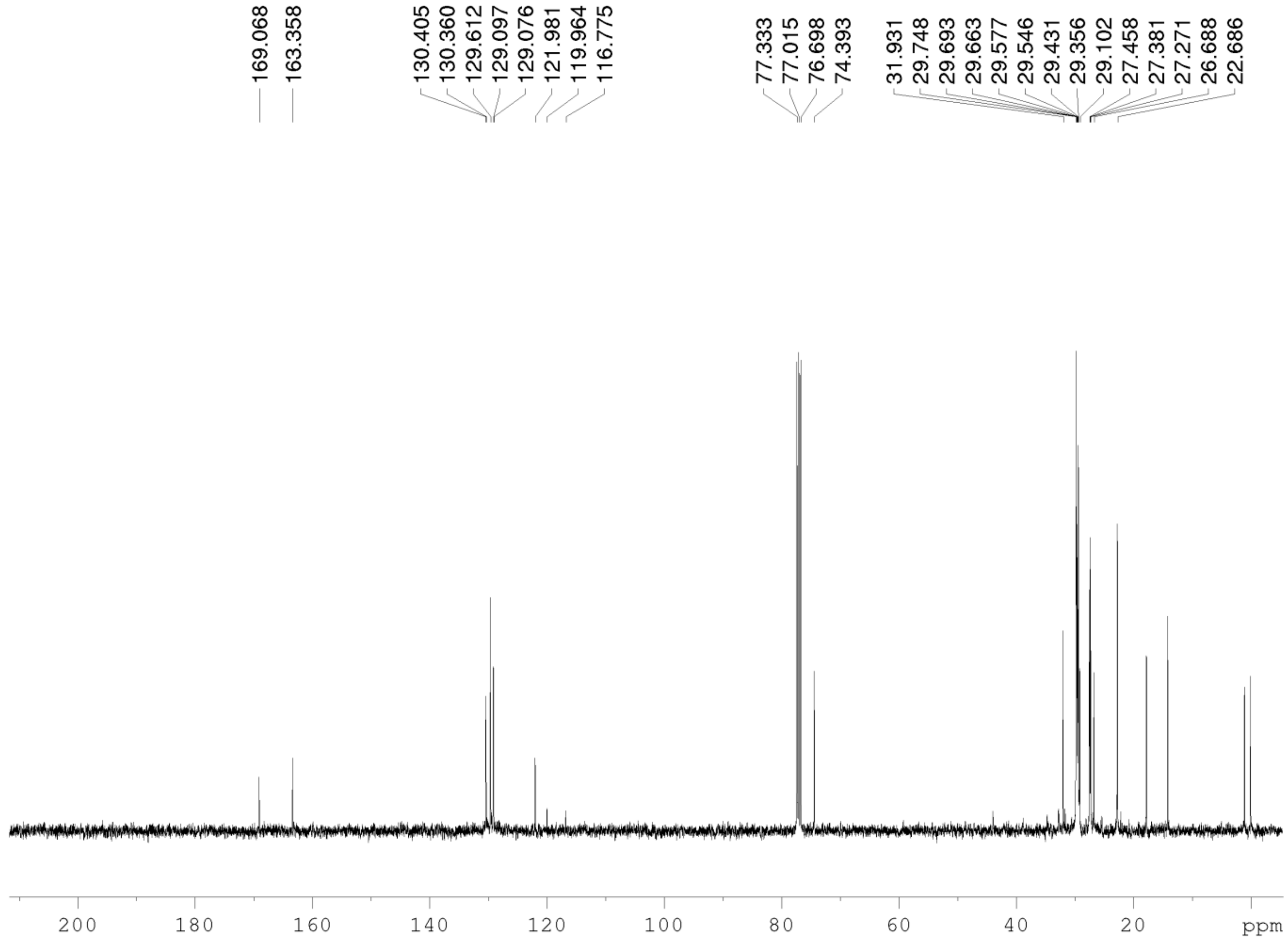

Figure S22. ^{1}H NMR Spectrum of compound **38** (400 MHz, $CDCl_3$)

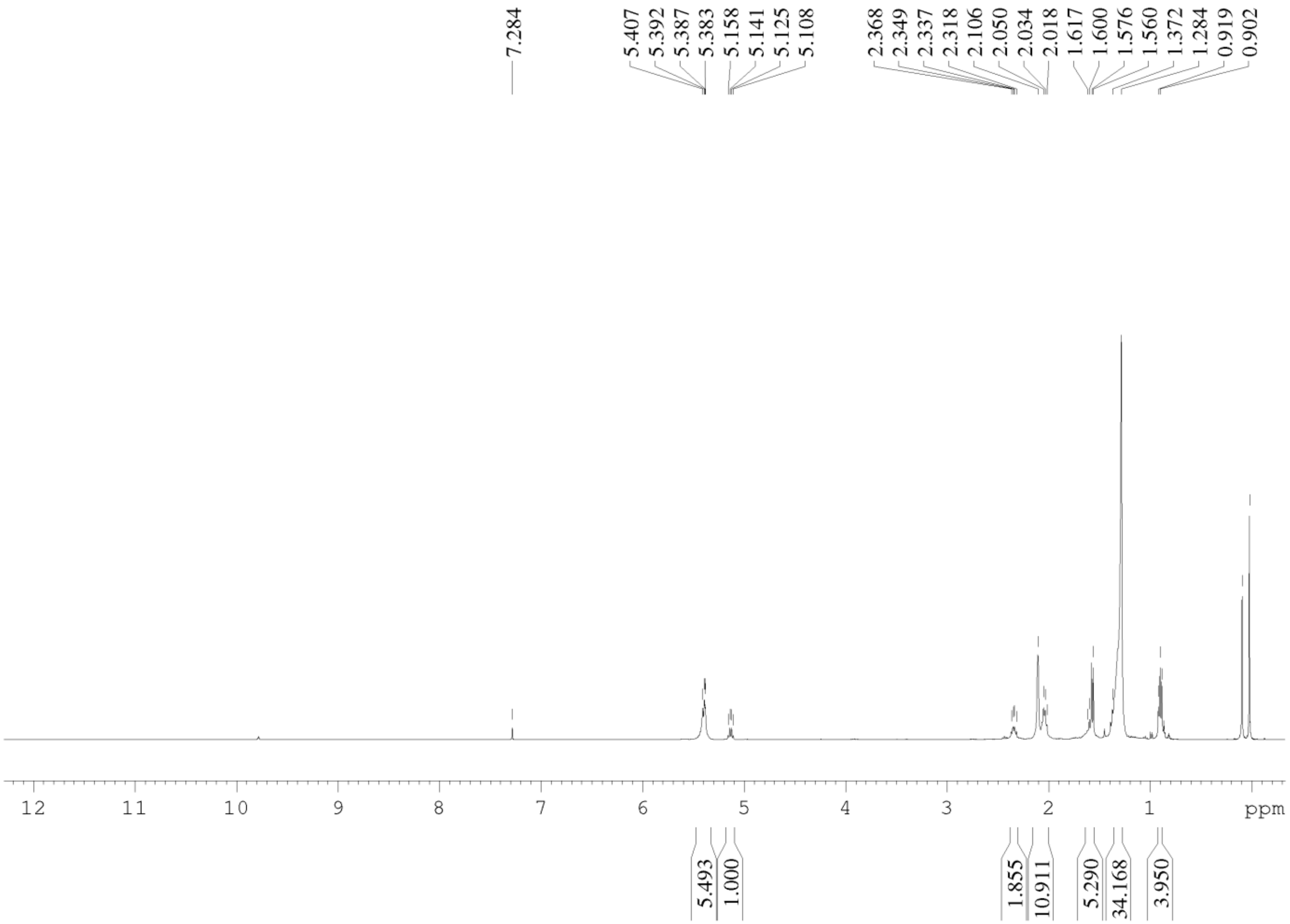

Figure S23. ^{13}C NMR Spectrum of compound **39** (125 MHz, $CDCl_3$)

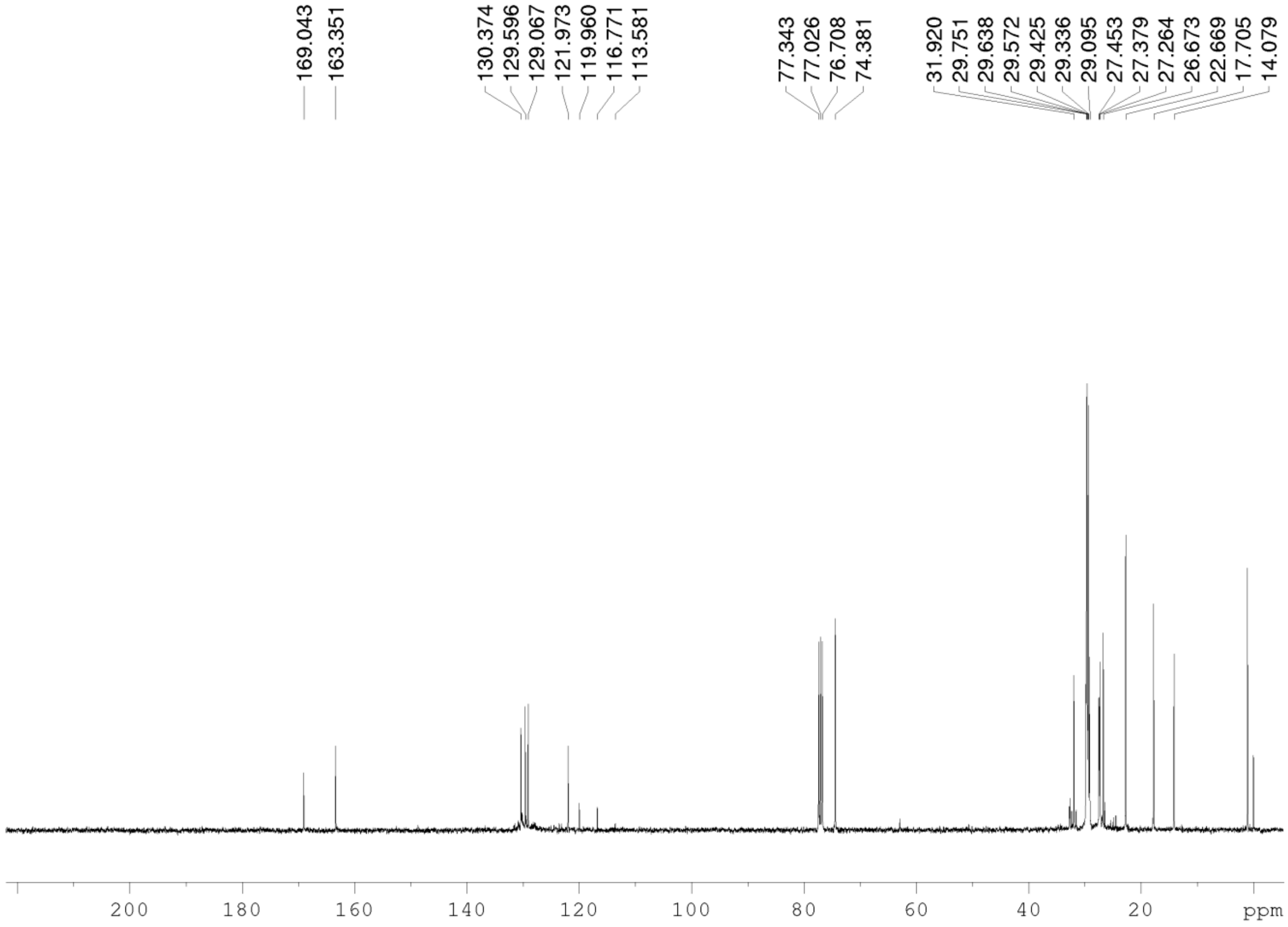

Figure S24. ^{1}H NMR Spectrum of compound **39** (500 MHz, $CDCl_3$)

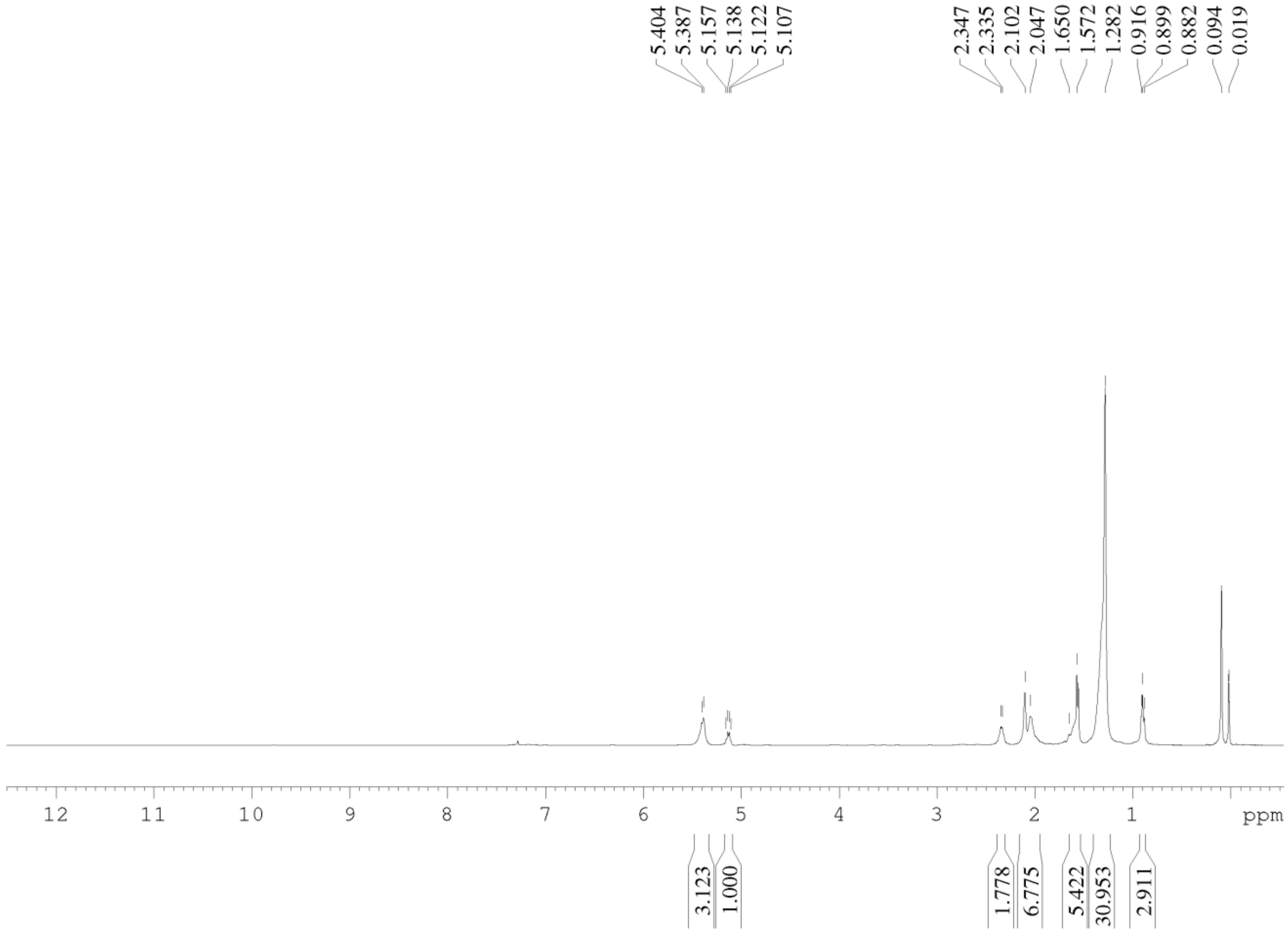

Figure S25. ^{13}C NMR Spectrum of compound **2 (**Chatenaytrienin-2) (100 MHz, $CDCl_3$)

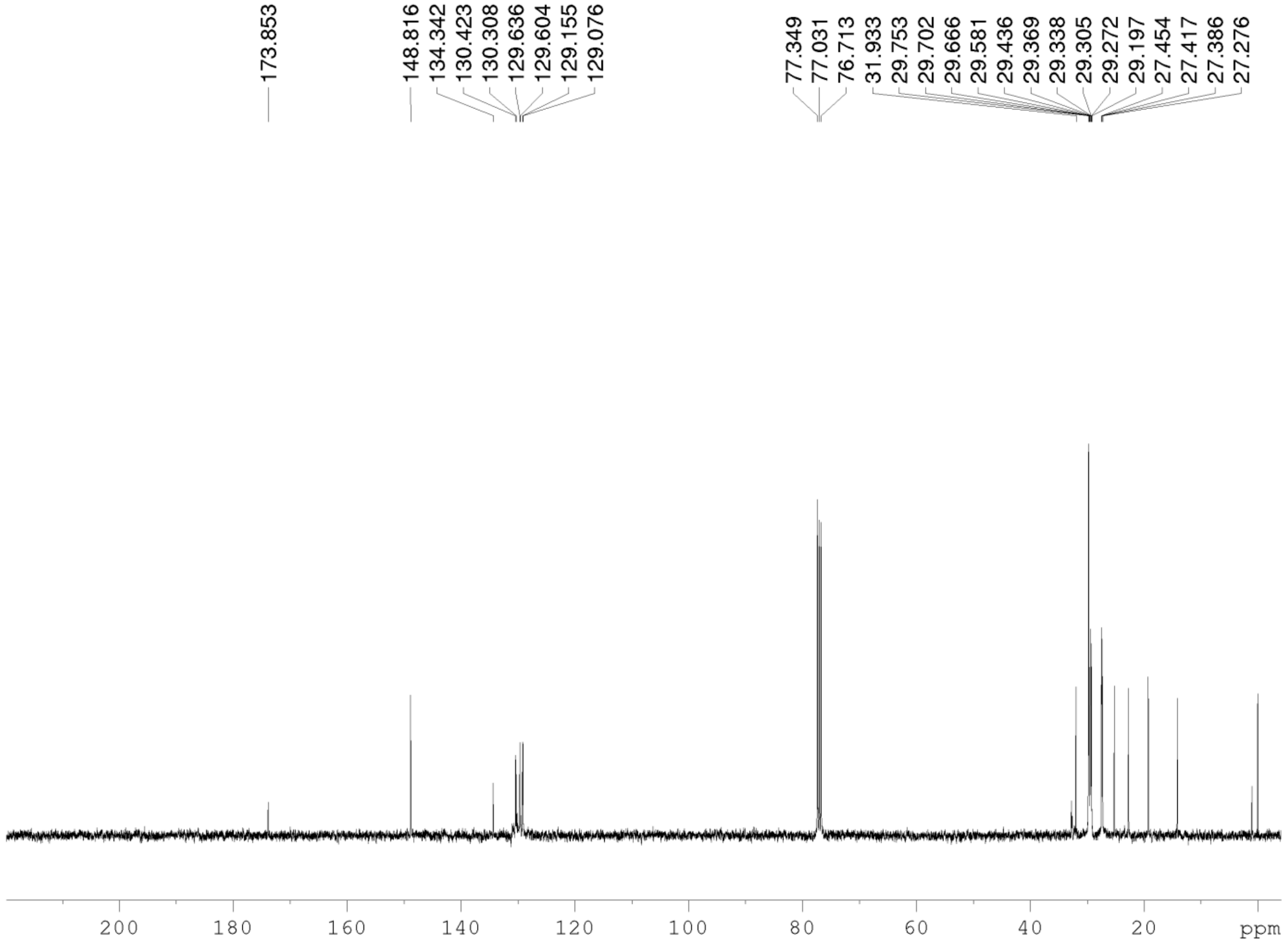

Figure S26. ^{1}H NMR Spectrum of compound **2** (Chatenaytrienin-2) (400 MHz, $CDCl_3$)

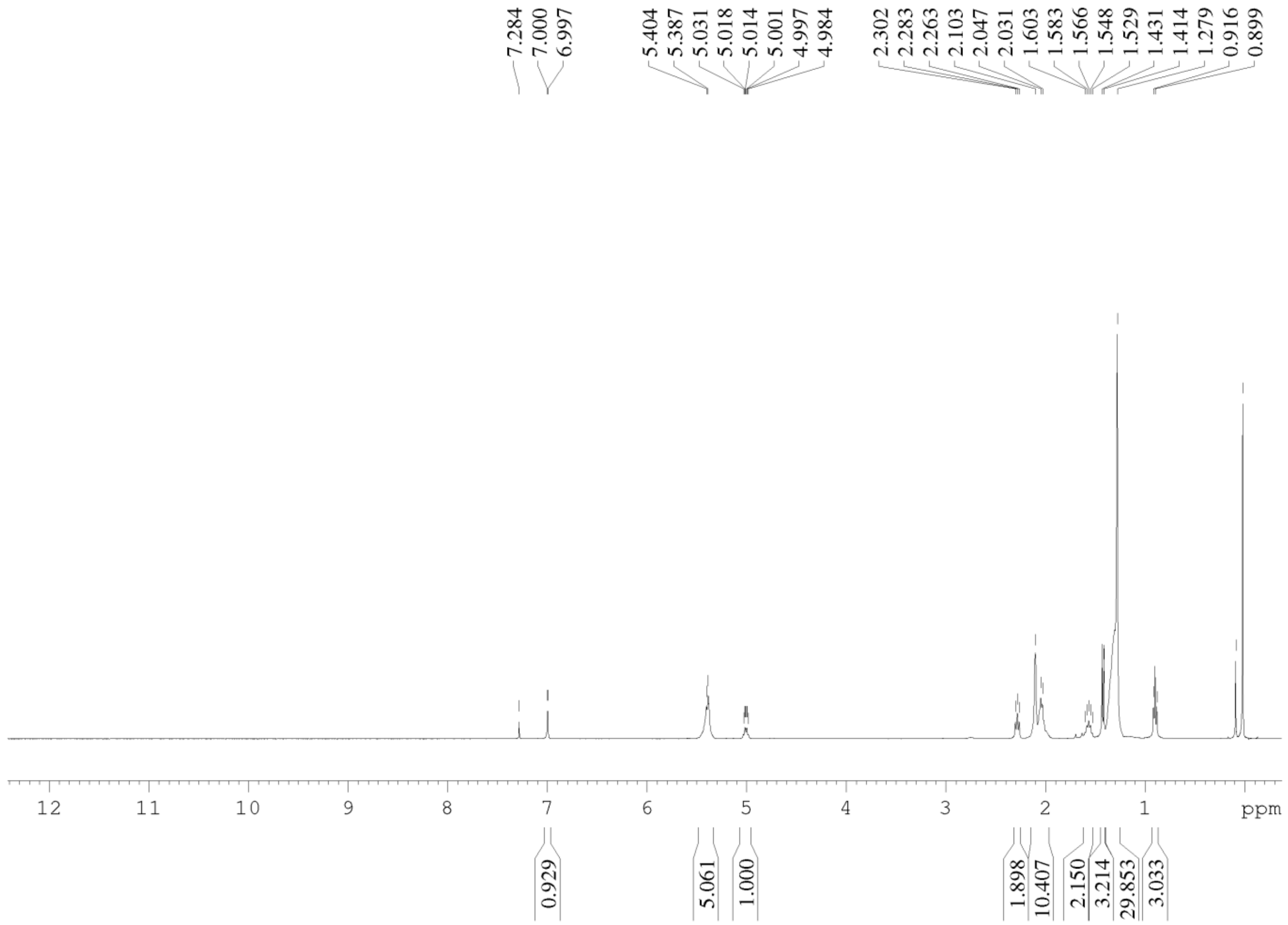

Figure S27. ^{13}C NMR Spectrum of compound **3 (**Chatenaytrienin-3**)** (100 MHz, $CDCl_3$)

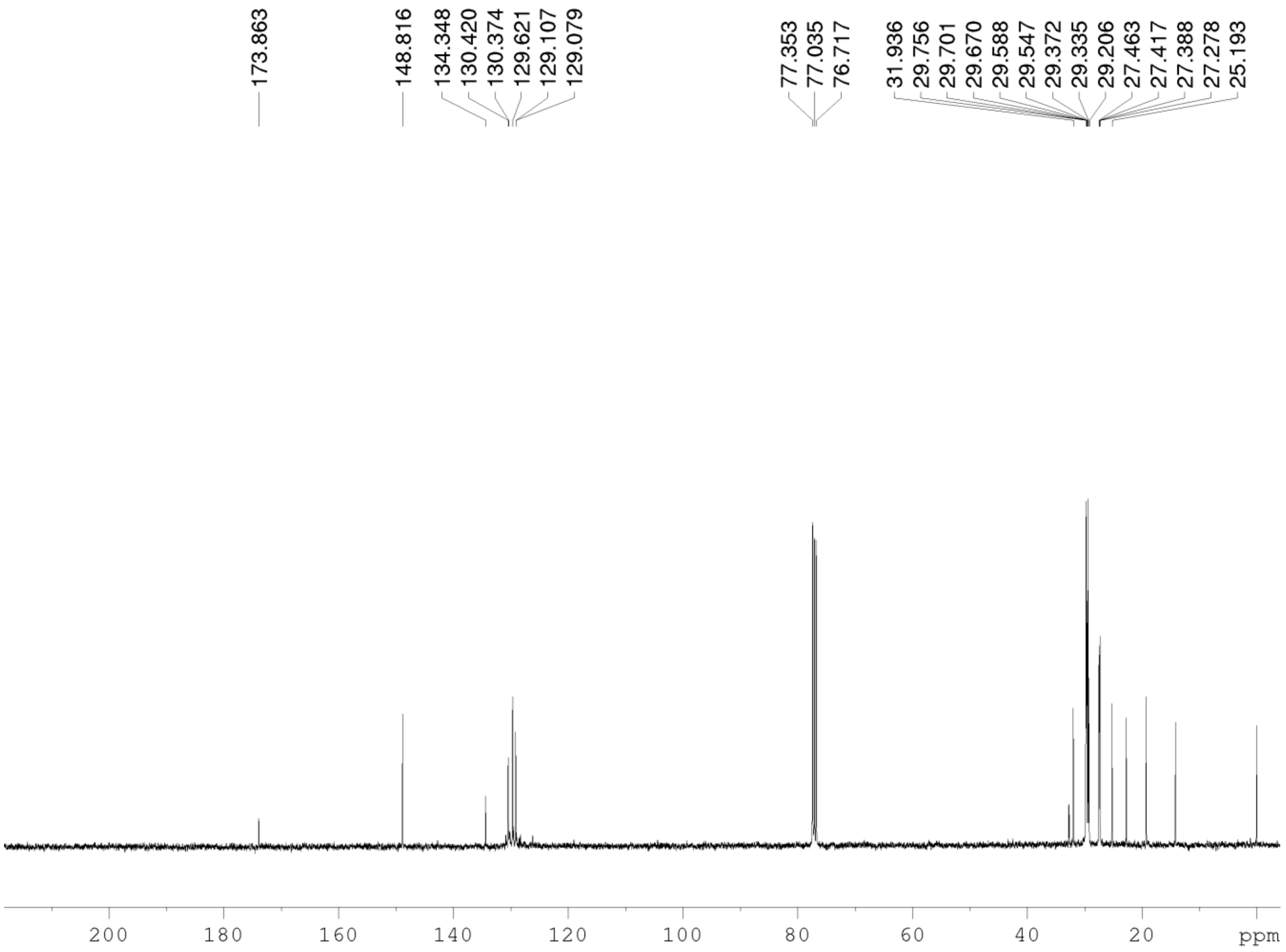

Figure S28. ^{1}H NMR Spectrum of compound **3** (Chatenaytrienin-3) (400 MHz, $CDCl_3$)

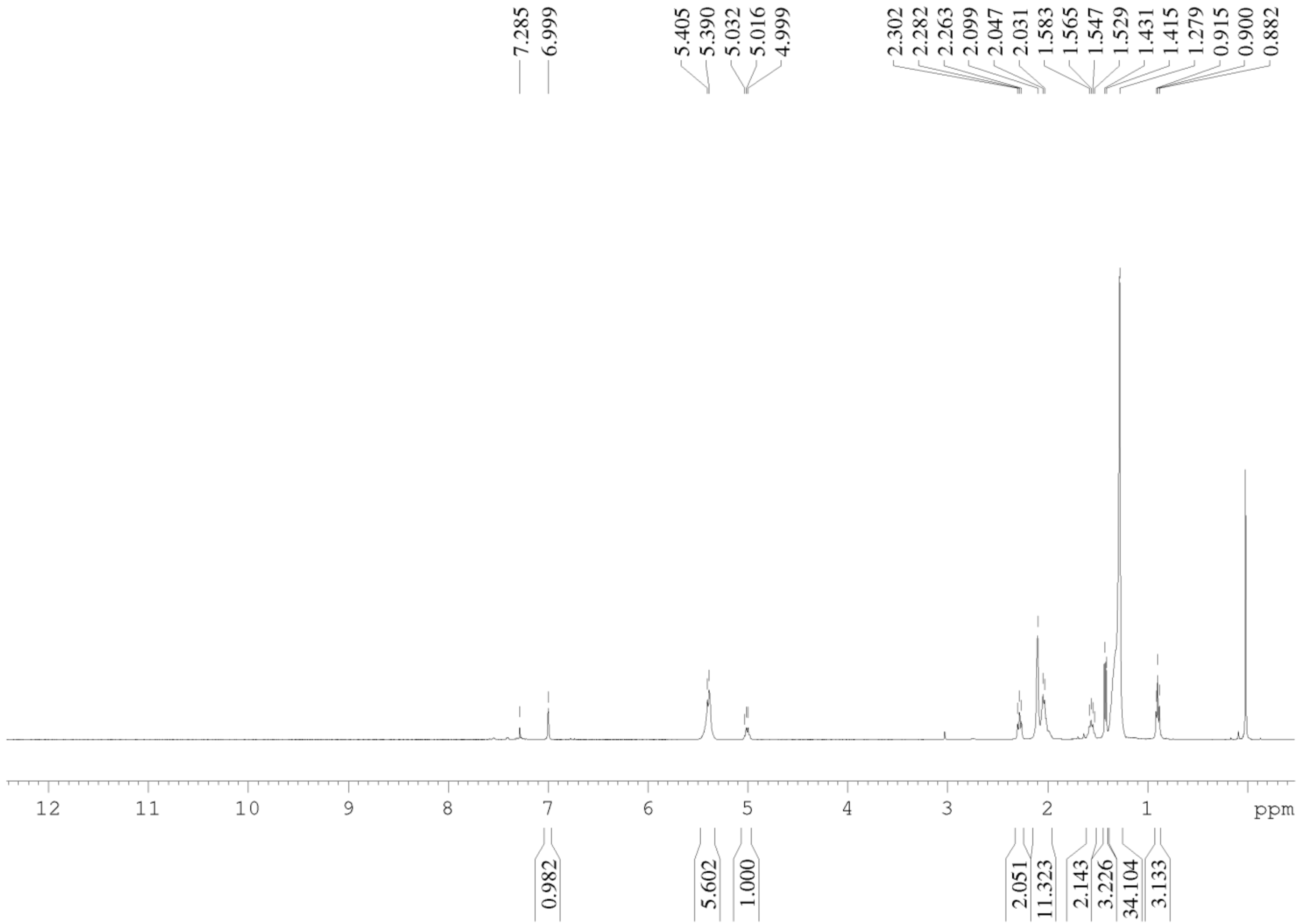

Figure S29. ^{13}C NMR Spectrum of compound **4** (Chatenaytrienin-4) (100 MHz, $CDCl_3$)

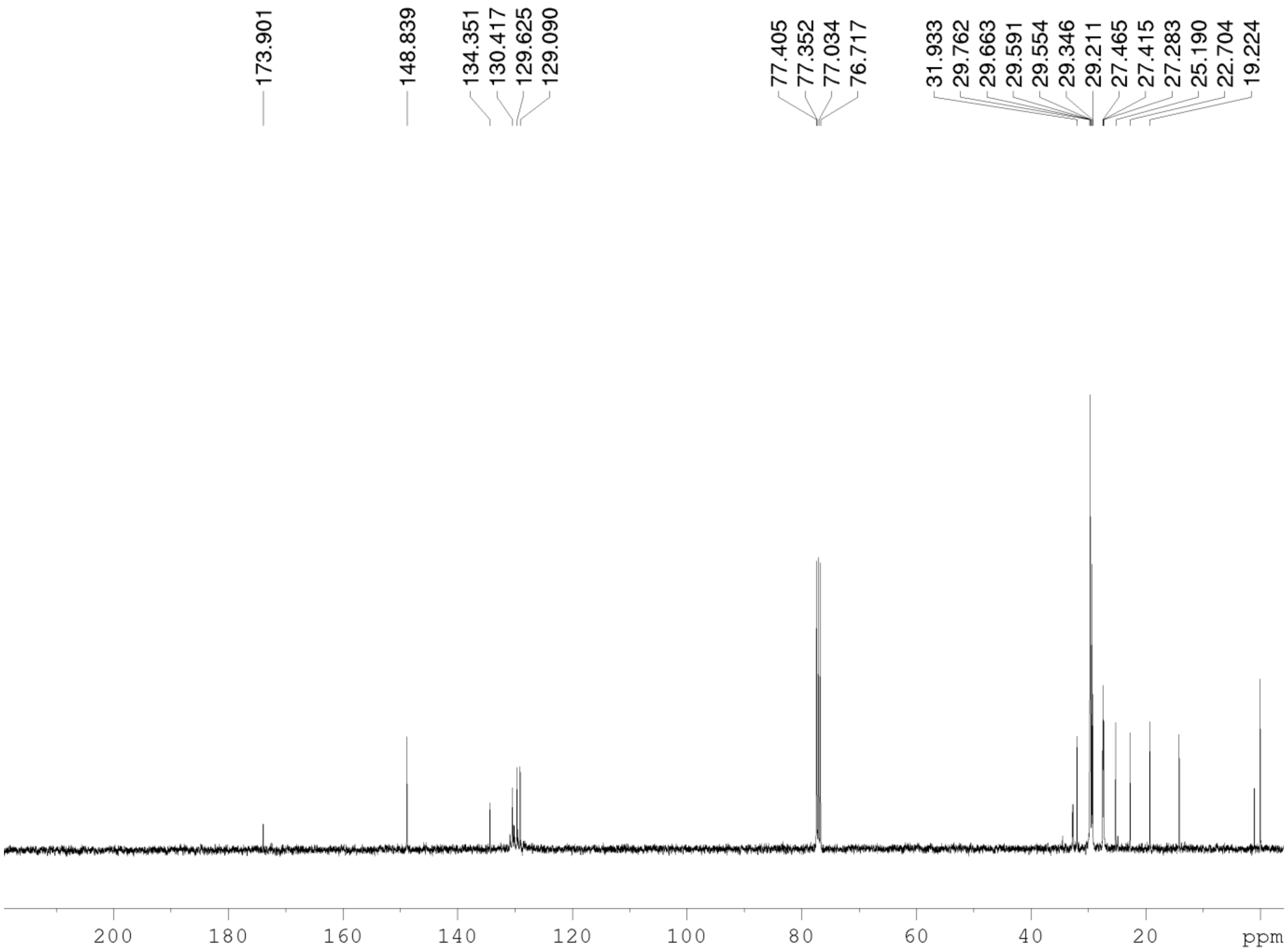

Figure S30. ^{1}H NMR Spectrum of compound **4** (Chatenaytrienin-4) (400 MHz, $CDCl_3$)

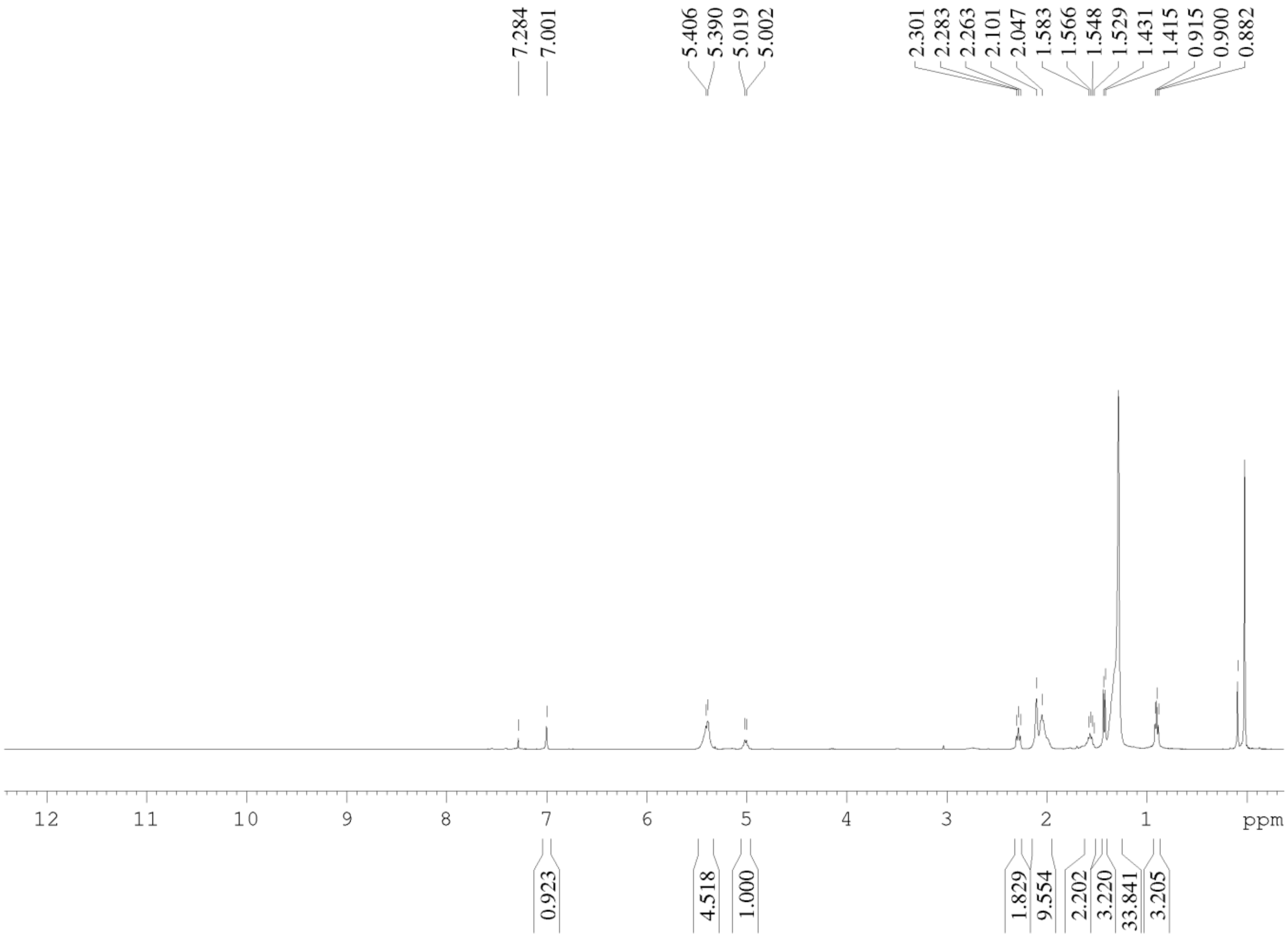
7.284
7.001
5.406
5.390
5.019
5.002
2.301
2.283
2.263
2.101
2.047
1.583
1.566
1.548
1.529
1.431
1.415
0.915
0.900
0.882
12
11
10
9
8
7
6
5
4
3
2
1
ppm
0.923
4.518
1.000
1.829
9.554
2.202
3.220
33.841
3.205

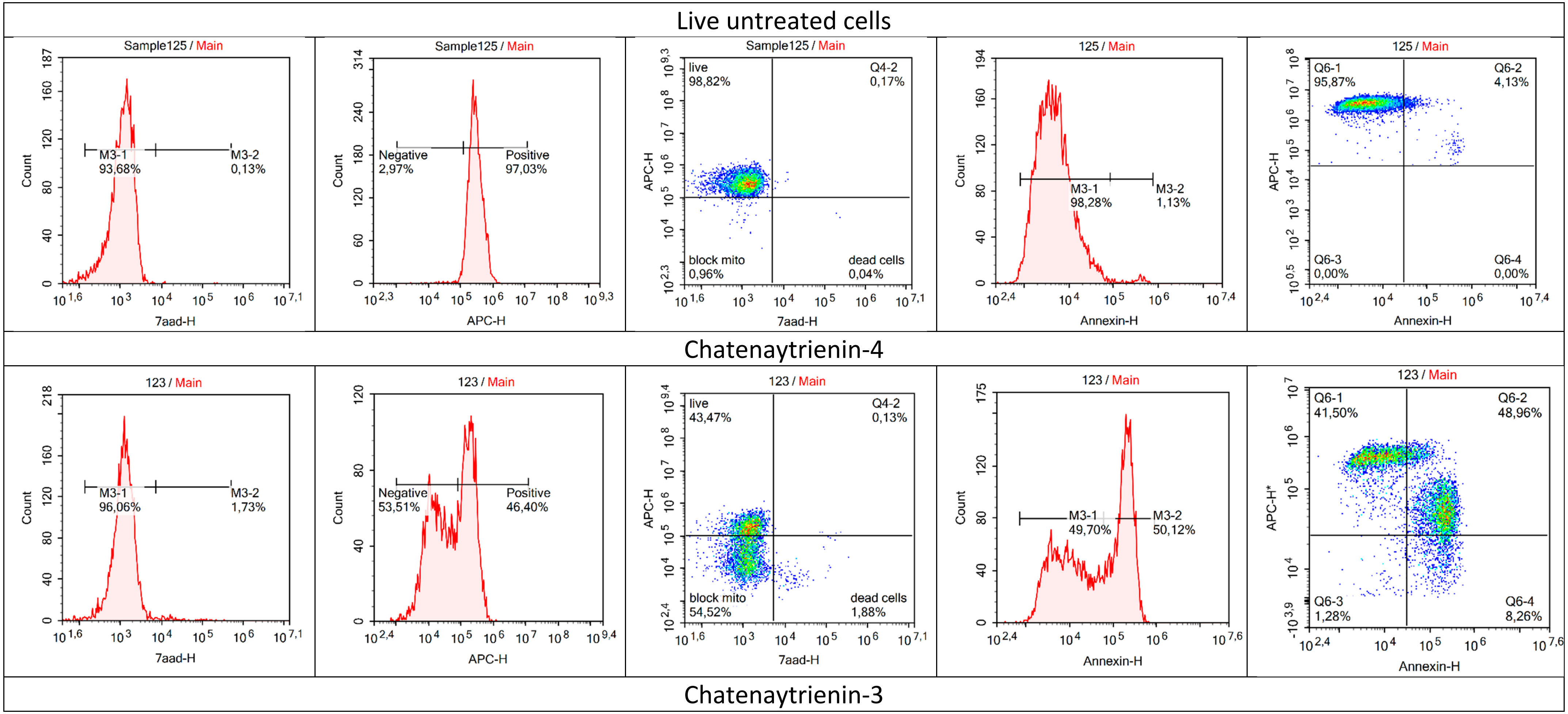

Live untreated cells
Sample125 / Main
M3-1
93,68%
M3-2
0,13%
Count
7aad-H
Negative
2,97%
Positive
97,03%
APC-H
live
98,82%
Q4-2
0,17%
block mito
0,96%
dead cells
0,04%
125 / Main
M3-1
98,28%
M3-2
1,13%
Annexin-H
Q6-1
95,87%
Q6-2
4,13%
Q6-3
0,00%
Q6-4
0,00%
Chatenaytrienin-4
123 / Main
M3-1
96,06%
M3-2
1,73%
Negative
53,51%
Positive
46,40%
live
43,47%
Q4-2
0,13%
block mito
54,52%
dead cells
1,88%
M3-1
49,70%
M3-2
50,12%
Q6-1
41,50%
Q6-2
48,96%
Q6-3
1,28%
Q6-4
8,26%
APC-H*
Chatenaytrienin-3

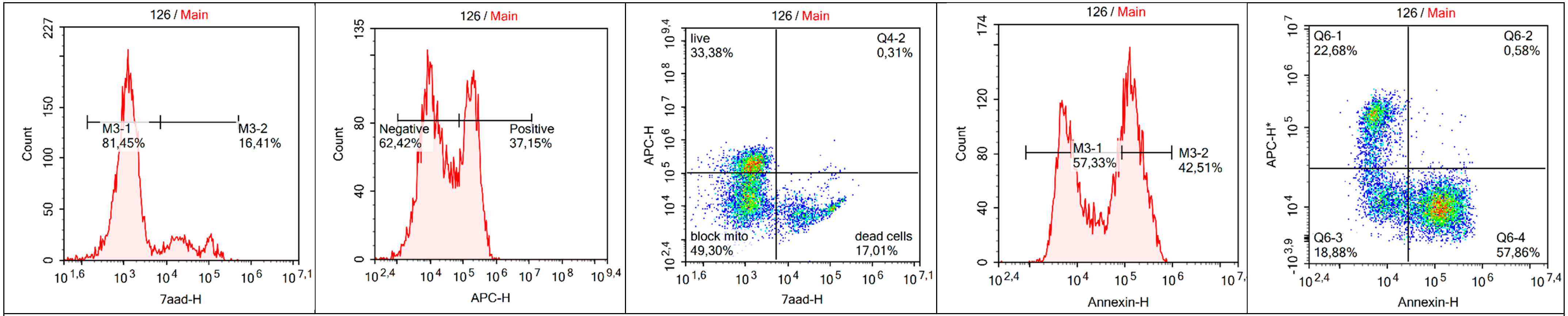

Chatenaytrienin-2

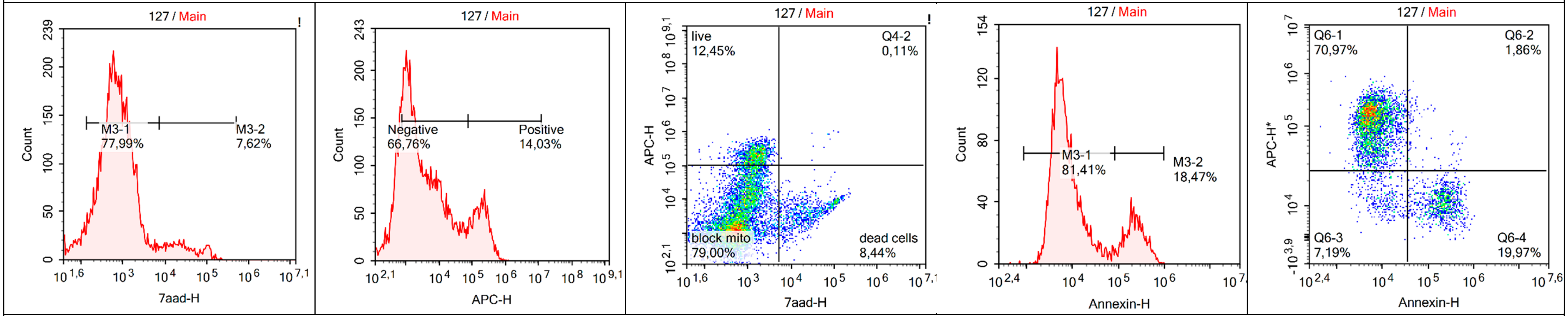

Chatenaytrienin-1

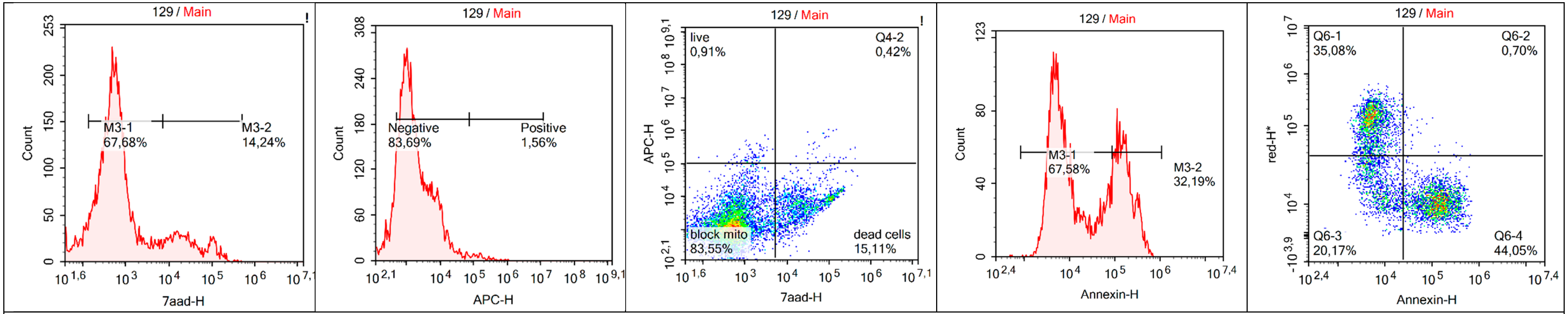

Staurosporine

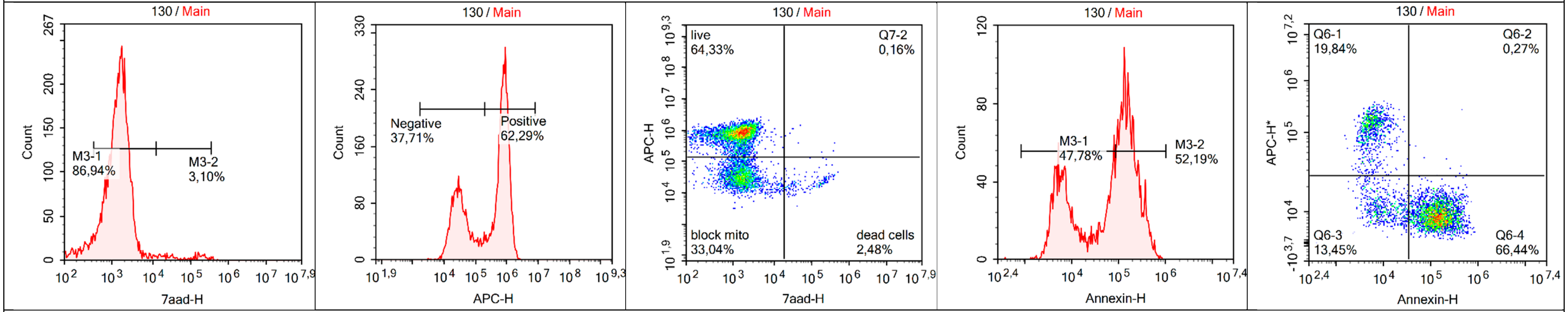

Table S1 with 95% confidence intervals are provided in parentheses

	Control cells	1	2	3	4	Staurosporin
Mitohondria live	98.82 (97.77 – 99.11)	0.91 (0.85 – 0.99)	12.45 (11.99 – 13.95)	33.38 (31.44 – 34.96)	43.47 (42.26 – 44.15)	64.33 (62.75 -65.55)
Mitohondria dead	0.96 (0.90 – 0.98)	83.55 (80.12 – 84.44)	79.00 (76.57 – 80.01)	49.30 (48.09 – 50.98)	54.52 (52.64 – 55.16)	33.04 (32.35 – 35.35)
Necrosis	0 (0.0 – 0.01)	15.11 (14.13 – 16.15)	8.44 (7.99 -9.04)	17.01 (16.11 – 18.12)	1.88 (0.99 – 2.01)	2.48 (1.15 – 2.99)
Live	95.87	35.08	70.97	22.68	41.50	19.84

	(93.46 – 97.33)	(34.07 – 36.87)	(69.84 – 71.14)	(20.17 – 23.23)	(39.74 – 42.02)	(18.17 – 20.06)
Early apoptosis	0<br>(0.0 – 0.02)	20.17<br>(19.19 – 21.20)	1.86<br>(0.99 – 2.06)	18.88<br>(17.05 – 19.96)	48.96<br>(47.47 – 49.11)	0.27<br>(0.25 – 0.29)
Late apoptosis	4.13<br>(3.99 – 4.38)	44.05<br>(42.32 – 45.16)	19.97<br>(18.88 – 20.08)	57.86<br>(55.77 – 58.85)	8.26<br>(7.16 – 9.02)	66.44<br>(64.12 – 67.85)

Figure S31. Detection of changes in the mitochondrial membrane potential (ΔΨ) in Jurkat cells treated with chatenaytrienins-1, -2, -3 and -4 (**1–4**), taken at a concentration of IC50: (A) control sample containing living cells; (B) chatenaytrienin-4 (**4**); (C) chatenaytrienin-3 (**3**); (D) chatenaytrienin-2 (**2**); (E) chatenaytrienin-1 (**1**); (G) staurosporine. The cells were stained with MitoSense Red, Annexin V – with CF488A and 7-AAD (FlowCellect® MitoDamage Kit). Incubation time was 4 hours. Cytofluorometric analysis covered the data of three independent experiments. 95% confidence intervals are provided in parentheses in Table S1.

Untreated cells

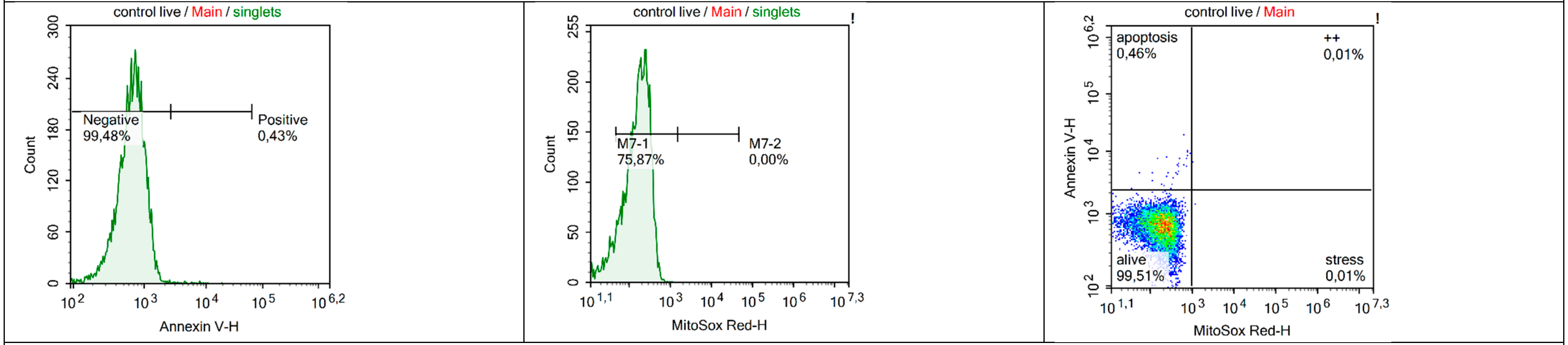

Chatenaytrienin-1

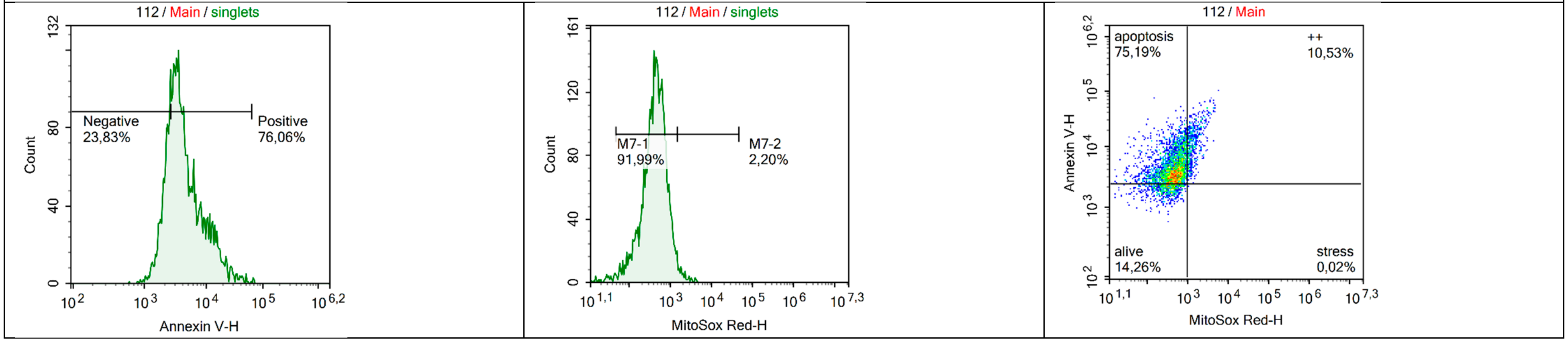

Chatenaytrienin-2

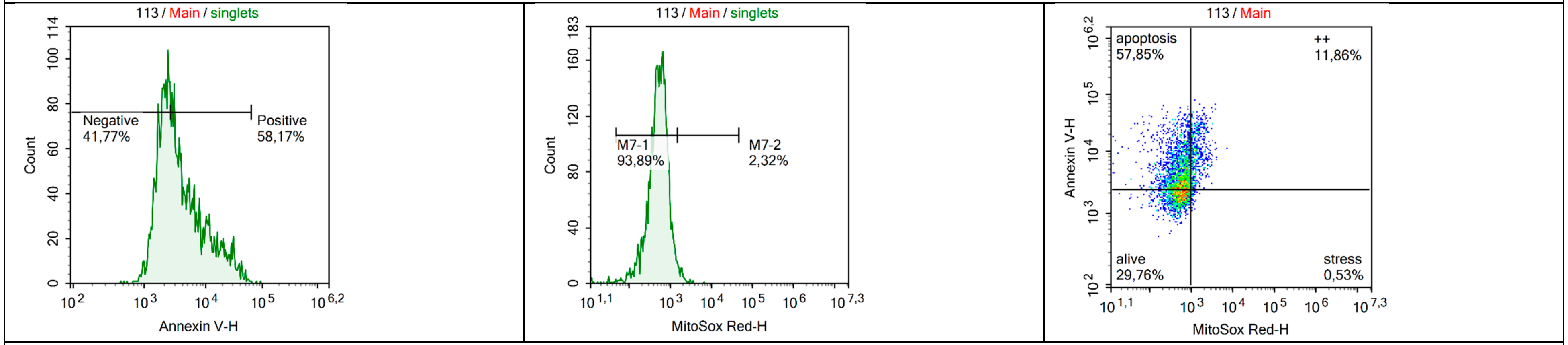

Chatenaytrienin-3

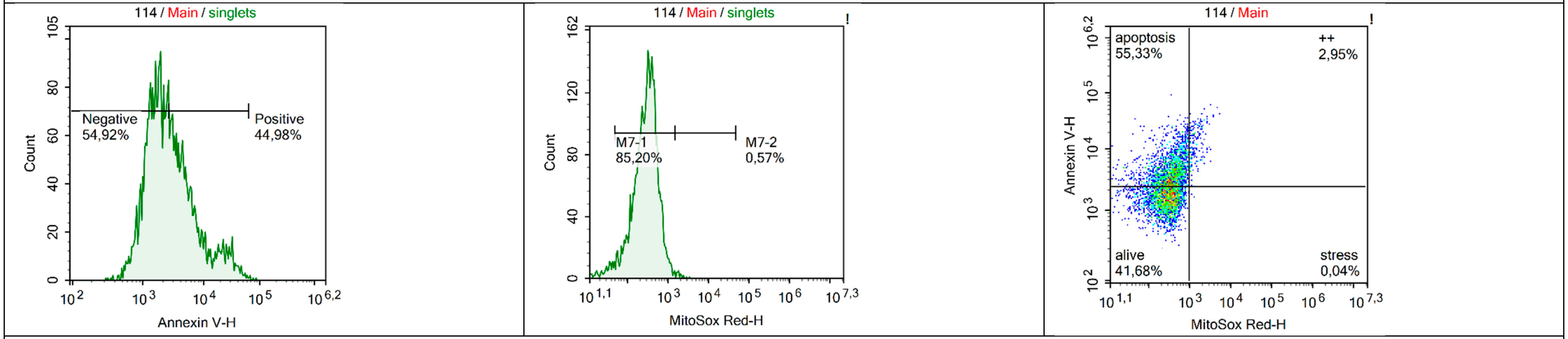

Chatenaytrienin-4

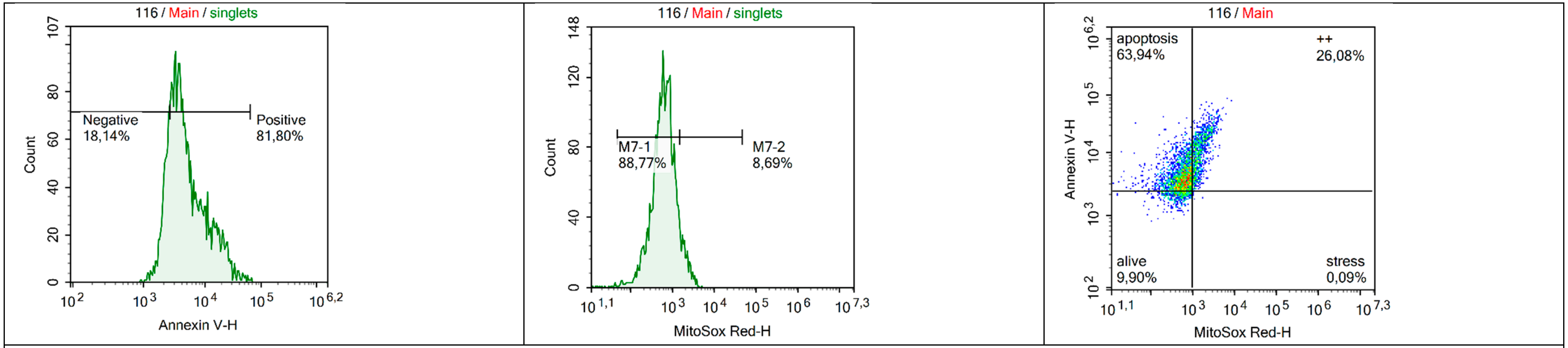

Antimycin

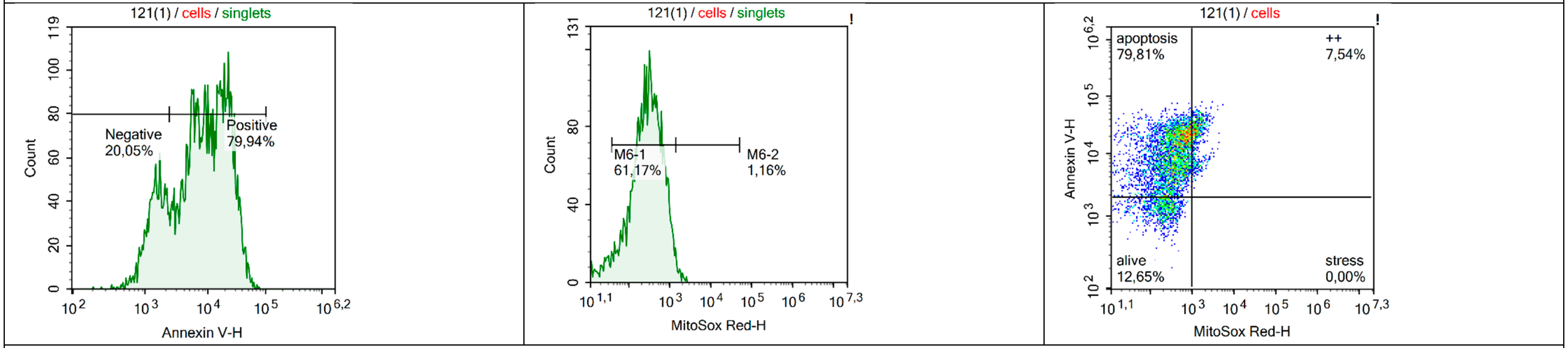

Table S2 with 95% confidence intervals are provided in parentheses

	Control	1	2	3	4	Antimycin
Live cells	99.51 (97.45 -99.99)	14.26 (13.66 – 16.17)	29.76 (26.11 -30.01)	41.68 (40.15 – 43.06)	9.9 (8.16 – 10.17)	12.65 (11.15 – 13.36)
Apoptosis cells	0.46 (0.56-0.51)	75.19 (73. 78 – 76.67)	57.85 (56.65 – 58.11)	55.33 (54.54 -56.84)	63.94 (60.14 – 65.12)	79.81 (77.11 – 80.01)
Stress cells	0 (0 – 0.01)	0.02 (0.0 – 0.03)	0.53 (0.46 – 0.570	0.04 (0.02 – 0.05)	0.09 (0.07 – 0.10)	0 (0.0 -0.01)

Apoptosis+Stress cells	0<br>(0.0 – 0.02)	10.53<br>(9.88 -10.87)	11.86<br>(10.11 – 13.06)	2.95<br>(1.99 – 3.07)	26.08<br>(25.12 – 27.43)	7.54<br>(7.01 – 7.98)

Figure S32. Detection of oxidative stress and early apoptosis events in Jurkat cells treated with chatenaytrienins-1, -2, -3 and -4 (**1**–**4**) taken at a concentration IC50: (A) control sample; (B) chatenaytrienin-1 (**1**); (C) chatenaytrienin-2 (**2**); (D) chatenaytrienin-3 (**3**); (E) chatenaytrienin-4 (**4**); (F) antimycin (100µM). MitoSOX Red and Annexin V stain, CF647 (FlowCellect™ MitoStress Kit). Incubation time was 4 hours. Cytofluorometric analysis involved the data of three independent experiments. 95% confidence intervals are provided in parentheses in Table S2.

1	2	3
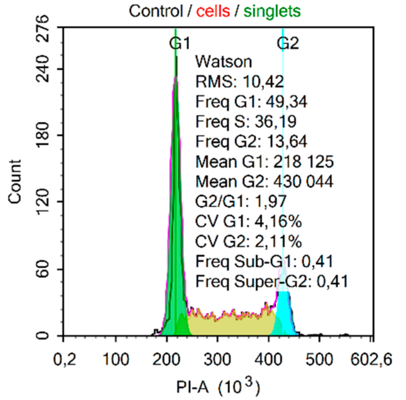	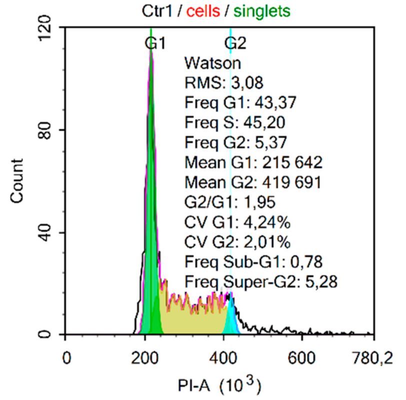	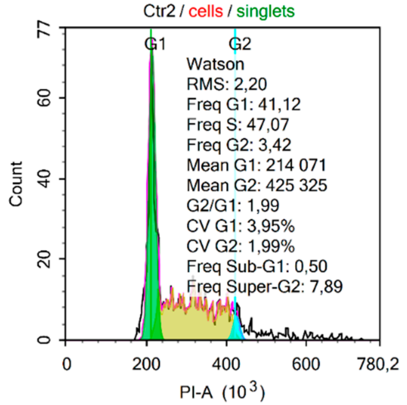
4	5	

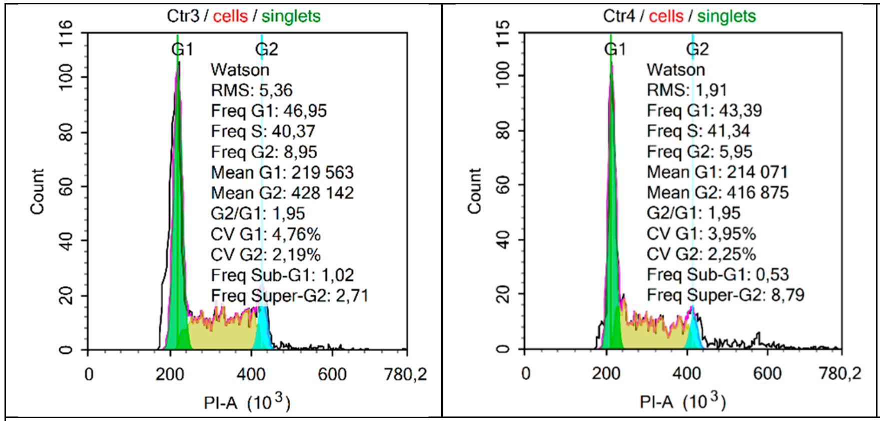

Table S3 with 95% confidence intervals are provided in parentheses

	Control	1	2	3	4
G1	49.34 (44.15- 50.11)	43.37 (41.55 – 44.43)	41.12 (39.39 -42.11	46.95 (44.54 – 47.77	43.39 (42.34 – 45.15)
S	36.9 (35.16- 37.18)	42.2 (39.18 – 43.43)	47.07 (45.65 -48.19)	40.37 (39.90 – 41.45)	41.34 (39.45 – 43.01)
G2/M	13.64 (11.43 – 14.23)	5.37 (4.32 – 6.74)	3.42 (2.23 – 3.98)	8.95 (7.15 – 9.20)	5.95 (4.15 – 6.18)
subG0	0.41 (0.46 – 0.49)	0.78 (0.73 – 0.79)	0.5 (0.35 – 0.56)	1.02 (0.99 – 1.15)	0.53 (0.45 – 0.65)

Figure S33. Histograms of cytofluorometric analysis of cell cycle phases in Jurkat cells under the influence of the studied acetogenins. The cells treated with chatenaytrienins-1. -2. -3 and -4 (**1**–**4**) at IC50: (1) control; (2) chatenaytrienin-1 (**1**); (3) chatenaytrienin-2 (**2**); (4) chatenaytrienin-3 (**3**); (5) chatenaytrienin-4 (**4**). Guava® Cell Cycle Reagent stain. The time of incubation of compounds with cells was 48 hours. All results of cytofluorometric analysis were obtained from at least three independent experiments. 95% confidence intervals are provided in parentheses in Table S3.

Data from MILLIPLEX® 7-plex Early Apoptosis Magnetic Bead Kit and MILLIPLEX® MAP 7-plex DNA Damage/Genotoxicity Magnetic Bead Kit used in Jurkat cells upon 6-h and 12-h exposure to test substances.

	**Chk2**		**Chk1**		**MDM2**		**H2A.X**		**p21**		**ATR**	
	**6 h**	**12 h**	**6h**	**12h**	**6h**	**12h**	**6h**	**12h**	**6h**	**12h**	**6h**	**12h**
**Chate-naytrienin-1**	19.54 (18.30 -21.23)	85.52 (83.88-87.93)	9.53 (8.15-10.25)	89.44 (88.95-90.11)	15.55 (13.28-18.75)	231.88 (230.15-233.18)	1724.65 (1699.94-1768.87)	2004.58 (1998.13-2015.44)	15.58 (13.45-17.80)	79.15 (76.16-80.33)	51.96 (49.99-54.86)	145.20 (142.34 147.18)
**Murica-dienin**	4.85 (5.11-6.18)	79.45 (78.54-79.37)	4.53 (3.58 -5.86)	80.55 (79.14-82.13)	31.76 (29.87-34.77)	217.56 (216.12-222.16)	2397.45 (2254.43 – 2678.32)	2561.54 (2558.16-2570.99)	1.5 (1.05-2.56)	71.59 (68.45-73.22)	58.33 (55.43 – 60.23)	140.99 (138.46-142.20)
**Control**	735.11 (720.33-740.28)	703.48 (701.53-705.37)	1316.35 (1333.76-1340.29)	1206.57 (1193.66-1230.59)	168.61 (163.45-170.38)	166.61 (162.40-168.35)	118.76 (115.89-120.96)	112.35 (110.43-115.87)	65.87 (59.99-68.55)	59.44 (57.32-61.30)	63.86 (60.25-66.75)	61.58 (59.32-62.00)

	**BAD**		**Cas8**		**Bcl-2**		**Cas9**		**JNK**		**p53**	
	**6h**	**12h**	**6h**	**12h**	**6h**	**12h**	**6h**	**12h**	**6h**	**12h**	**6h**	**12h**
**Chate-naytrienin-1**	18.94 (16.55-19.99)	151.49 (149.14-153.28)	8.65 (6.87-9.96)	83.35 (81.12-85.74)	4.51 (3.33-5.55)	56.78 (53.76-58.12)	2.75 (1.05–3.45)	80.33 (77.65-82.54)	218.66 (201.43-225.76)	575.76 (570.43-578-44)	670.11 (666.23–678.30)	80.94 (78.25–83.94)
**Murica-dienin**	13.56 (11.64-15.65)	152.28 (149.34-155.18)	10.5 (9.99-12.11)	87.16 (84.97-89.65)	1.5 (1.02-2.18)	53.99 (49.99-54.89)	4.25	79.85 (75.43-81.15)	586.90 (580.99-589.98)	328.98 (323.35-330.65)	586.31 (580.77–590.35)	697.99 (690.32-699.86)
**Control**	801.45 (798.68-840.25)	864.76 (860.88-866.45)	80.67 (79.16-85.15)	86.24 (83.77-88.25)	65.18 (62.12-68.87)	54.18 (52.11-55.98)	80.99 (79.01-72.38)	75.76 (72.78-77.34)	520.18 (505.37-538.59)	530.18 (527.67-533.57)	1030.18 (1006.88-1044.36)	1099.50 (1078.24 – 1112.65)

Data from MILLIPLEX Multi-Pathway Magnetic Bead 9-Plex - Cell Signaling Multiplex Assayused in Jurkat cells upon 4-h exposure to test substances.

	CREB	JNK	NFkB	p38	ERK1/2	Akt	p70S6K	STAT3	STAT5
**Chate-naytrienin-1**	244.87 (230.13-250.67)	225.15 (220.14 – 228.96)	73.54 (70.87 – 76.22)	763.84 (758.20 – 773.12)	183.59 (179.25 – 187.54)	749.88 (745.55 – 756.16)	149.85 (145.15 – 152.65)	123.65 (120.14 – 126.88)	93.59 (89.99 – 95.14)
**Murica-dienin**	281.15 (278.46- 285.97)	301.97 (299.14 – 305.45)	62.96 (60.15 – 67.89)	1083.01 (1006.33 – 115.12)	158.23 (150.11 - 161.20)	965.83 (950.34 – 970.57)	122.85 (119.74 – 126.74)	100.15 (98.15 – 105.88)	85.85 (80.20 - 88.15)
**Control**	544.74 (537.19 – 559.63)	104.34 (100.54 – 108.82)	70.10 (68.64 – 74.11)	2511.11 (2499.45 – 2567.17)	294.10 (290.76 – 298.16)	4269.13 (4250.45 - 4286.66)	128.38 (120.66 – 130.11)	102.44 (100.35 – 106.47)	85.14 (80.01 - 89.00)
